# m^6^A-modified circCacna1c regulates necroptosis and ischemic myocardial injury by inhibiting Hnrnpf entry into the nucleus

**DOI:** 10.1186/s11658-024-00649-8

**Published:** 2024-11-12

**Authors:** Yi Jia, Xiaosu Yuan, Luxin Feng, Qingling Xu, Xinyu Fang, Dandan Xiao, Qi Li, Yu Wang, Lin Ye, Peiyan Wang, Xiang Ao, Jianxun Wang

**Affiliations:** 1 School of Basic Medicine, Qingdao University, Qingdao, 266071 China; 2 Department of Cardiology, The Affiliated Hospital of Qingdao University, Qingdao University, Qingdao, 266003 China; 3 School of Nursing, Qingdao University, Qingdao, 266071 China

**Keywords:** circCacna1c, Cardiomyocyte necroptosis, Myocardial infarction, N^6^-methyladenosine, Hnrnpf

## Abstract

**Background:**

Circular RNAs (circRNAs) are differentially expressed in various cardiovascular diseases, including myocardial infarction (MI) injury. However, their functional role in necroptosis-induced loss of cardiomyocytes remains unclear. We identified a cardiac necroptosis-associated circRNA transcribed from the *Cacna1c* gene (circCacna1c) to investigate the involvement of circRNAs in cardiomyocyte necroptosis.

**Methods:**

To investigate the role of circCacna1c during oxidative stress, H9c2 cells and neonatal rat cardiomyocytes were treated with hydrogen peroxide (H_2_O_2_) to induce reactive oxygen species (ROS)-induced cardiomyocyte death. The *N*^6^-methyladenosine (m^6^A) modification level of circCacna1c was determined by methylated RNA immunoprecipitation quantitative polymerase chain reaction (MeRIP–qPCR) analysis. Additionally, an RNA pull-down assay was performed to identify interacting proteins of circCacna1c in cardiomyocytes, and the regulatory role of circCacna1c in target protein expression was tested using a western blotting assay. Furthermore, the MI mouse model was constructed to analyze the effect of circCacna1c on heart function and cardiomyocyte necroptosis.

**Results:**

The expression of circCacna1c was found to be reduced in cardiomyocytes exposed to oxidative stress and in mouse hearts injured by MI. Overexpression of circCacna1c inhibited necroptosis of cardiomyocytes induced by hydrogen peroxide and MI injury, resulting in a significant reduction in myocardial infarction size and improved cardiac function. Mechanistically, circCacna1c directly interacts with heterogeneous nuclear ribonucleoprotein F (Hnrnpf) in the cytoplasm, preventing its nuclear translocation and leading to reduced Hnrnpf levels within the nucleus. This subsequently suppresses Hnrnpf-dependent receptor-interacting protein kinase 1 (RIPK1) expression. Furthermore, fat mass and obesity-associated protein (FTO) mediates demethylation of m^6^A modification on circCacna1c during necrosis and facilitates degradation of circCacna1c.

**Conclusion:**

Our study demonstrates that circCacna1c can improve cardiac function following MI-induced heart injury by inhibiting the Hnrnpf/RIPK1-mediated cardiomyocyte necroptosis. Therefore, the FTO/circCacna1c/Hnrnpf/RIPK1 axis holds great potential as an effective target for attenuating cardiac injury caused by necroptosis in ischemic heart disease.

**Graphical Abstract:**

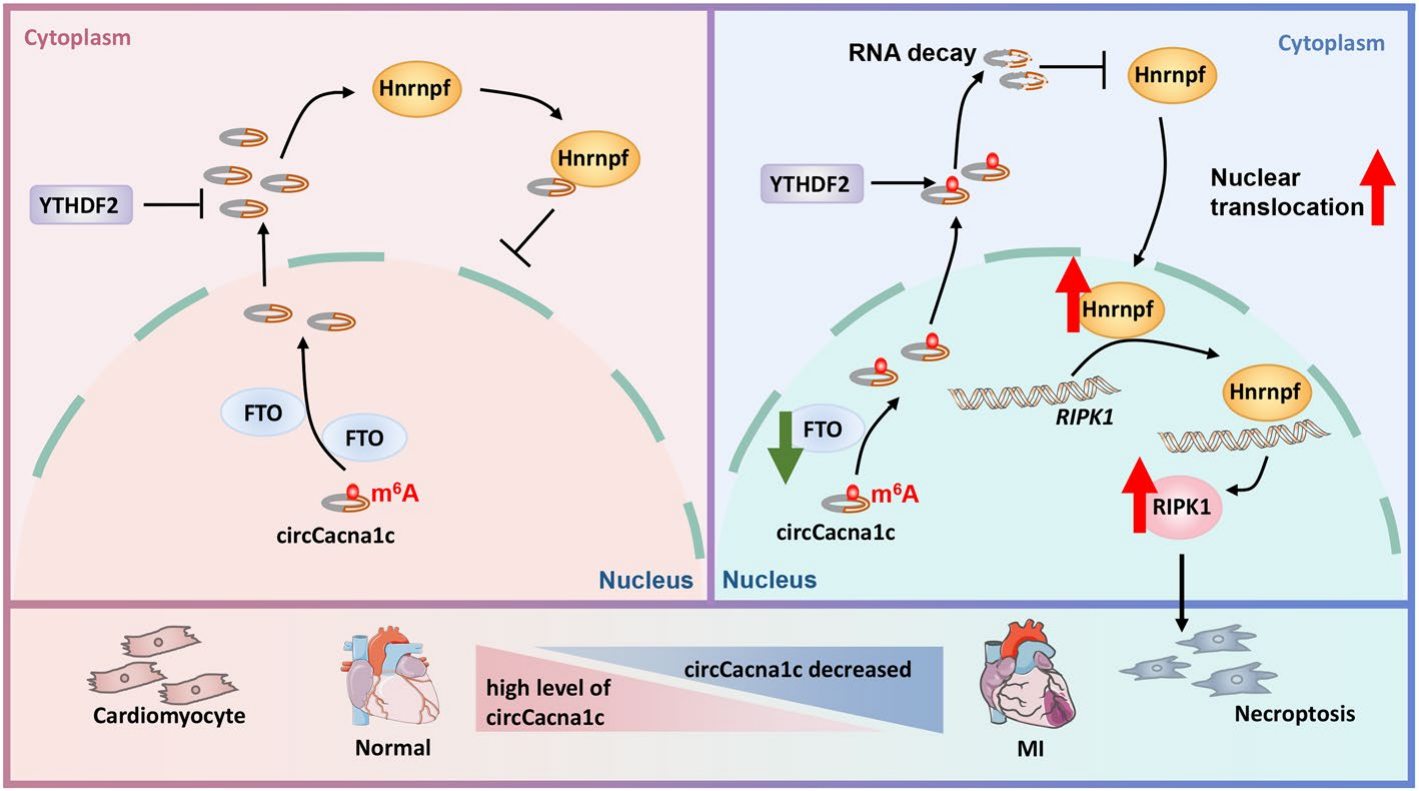

**Supplementary Information:**

The online version contains supplementary material available at 10.1186/s11658-024-00649-8.

## Background

Cardiomyocytes are post-mitotic cells that undergo terminal differentiation and cease to proliferate upon reaching maturity [1, 2]. Pathological insults such as ischemia–reperfusion (I/R) can result in cardiomyocyte death, leading to a significant loss of these cells. The adult heart has limited regenerative capacity, particularly following myocardial infarction (MI), where the ability of cardiomyocytes to regenerate is insufficient to counteract cell death or adequately restore the injured heart [3, 4]. Cardiomyocyte death has been shown to be the pathological basis of multiple cardiovascular diseases (CVDs) [5]. Therefore, exploring the mechanisms underlying cardiomyocyte death holds great potential in terms of preventing and treating MI.

Various mechanisms, such as apoptosis, ferroptosis, and necroptosis, contribute to the death of cardiomyocytes [6, 7]. Among these mechanisms, necroptosis represents a newly discovered form of programmed cell death that is extensively implicated in diverse biological processes ranging from organogenesis and aging to inflammation and CVDs [8]. The incidence of cardiomyocyte necroptosis following myocardial I/R injury has been reported to be significantly higher than that of apoptosis, with a nearly tenfold difference, suggesting the crucial role of necroptosis in the progression of MI [9]. The process of necroptosis is characterized by the distinctive signaling cascade involving receptor-interacting serine/threonine protein kinase (RIPK) 1 and 3 [10–12]. These kinases form complexes to phosphorylate the pseudokinase mixed lineage kinase domain-like protein (MLKL), thereby inducing necroptosis in cells. Elevated levels of RIPK1, RIPK3, and phosphorylated MLKL have been observed in various CVDs, including MI, atherosclerosis, stroke, abdominal aortic aneurysm, and thrombosis [13]. However, the precise mechanisms underlying the regulation of RIPK1 or RIPK3 remain poorly understood.

Circular RNA (circRNA) is a distinct class of single-stranded non-coding RNA (ncRNA) molecules characterized by a covalently closed-loop structure [14]. They play pivotal roles in almost all biological processes through diverse mechanisms, including sequestering microRNAs (miRNAs), interacting with RNA-binding proteins (RBPs), and serving as scaffolds for complex formation [15, 16]. Accumulating evidence demonstrates that dysregulation of circRNAs is closely associated with multiple CVDs, including MI, cardiac toxicity, and cardiac fibrosis [17–19]. Our previous study uncovered that circNCX1 was significantly upregulated in cardiomyocytes upon exposure to reactive oxygen species (ROS). Its upregulation increased the levels of pro-apoptotic gene cell death-inducing protein (CDIP1) by sequestrating miR-133a-3p, thereby facilitating cardiomyocyte apoptosis [20]. Aberrant expression patterns of circRNAs also exhibit great potential as biomarkers for the diagnosis and prognosis of CVDs [21]. However, studies on whether circRNA can regulate the necroptosis of cardiomyocytes in CVD progression are still limited.

The *N*^6^-methyladenosine (m^6^A) is a reversible epigenetic modification in messenger RNA (mRNA) and ncRNA, initiated by m^6^A methyltransferases such as METTL3, METTL14, and RMB15, and dynamically removed by m^6^A demethylases like FTO and ALKBH5 [22]. Previous studies have indicated that circRNAs are direct targets of m^6^A modification [23]. The m^6^A modification is found to decrease the stability of circRNAs in an YTH m^6^A RNA binding protein 2 (YTHDF2)-dependent manner. Functioning as an m^6^A reader protein, YTHDF2 recognizes and binds to m^6^A-modified circRNAs, thereby facilitating their degradation through endonuclease-mediated cleavage [24]. Additionally, m^6^A modification can also facilitate cap-independent translation of circRNAs, augment their function as miRNA sponges, and modulate the interaction between circRNAs and RBPs [23, 25]. Current studies have identified that m^6^A modification play a crucial role in the pathogenesis of numerous CVDs, particularly MI [26]. These findings strongly suggest that m^6^A modification may participate in MI progression by targeting circRNAs.

In this study, we identified a novel m^6^A-modified circRNA (mmu_circ_0001511), referred to as circCacna1c, which is transcribed from the second and third exons of the *Cacna1c* gene. We observed a downregulation of circCacna1c in the hearts of mouse with MI and hydrogen peroxide (H_2_O_2_)-treated cardiomyocytes. The stability of circCacna1c was found to be regulated by FTO-mediated m^6^A demethylation in a YTHDF2-dependent manner. We demonstrated that circCacna1c inhibited the binding of heterogeneous nuclear ribonucleoprotein F (Hnrnpf) to *RIPK1* mRNA by preventing the translocation of Hnrnpf into the nucleus, thereby protecting cardiomyocytes against necroptosis. Overall, our findings provide new insights into m^6^A-guided circRNA regulation and emphasize the crucial role of circCacna1c in modulating cardiomyocyte necroptosis.

## Methods

### Cell culture and treatment

The H9c2 cell line, derived from rat hearts, was obtained from the Shanghai Institutes for Biological Sciences. The H9c2 cells were then cultured in Dulbecco’s modified Eagle medium (DMEM, Servicebio) supplemented with 10% fetal bovine serum (FBS, Servicebio) and 1% penicillin–streptomycin solution (Solarbio). To induce oxidative stress, varying concentrations of hydrogen peroxide (H_2_O_2_, 500/100 μM) were applied to the H9c2 cells and neonatal rat cardiomyocytes [27]. Neonatal rat cardiomyocytes and cardiac fibroblasts were obtained from Wistar rat neonates under sterile conditions following established procedures [28]. The hearts of neonatal rats were washed with phosphate-buffered saline (PBS). The cardiac tissue was cut with ophthalmic scissors, then the tissue was repeatedly dispersed using a digestion solution containing trypsin (Sigma) and collagenase II (Sigma) at 37 °C. The collected tissues were subjected to cell digestion, and the resulting cells were resuspended in Dulbecco’s modified Eagle medium/F-12 (DMEM/F-12, Servicebio) augmented with 10% FBS and 1% penicillin–streptomycin solution. The cells underwent a 90-min incubation at a temperature of 37 °C, after which cardiomyocytes were isolated and purified using differential adherence separation technique with the inclusion of bromodeoxyuridine (BrdU, Sigma). Subsequently, the cells were cultured following the same protocol as H9c2 cells.

### Divergent PCR

The connection between the head and tail of circCacna1c was verified by performing PCR amplification with divergent primers and convergent primers. The specificity of PCR amplification was confirmed through agarose gel electrophoresis. The sequences of rat circCacna1c divergent primers and convergent primers can be found in supplementary table 1 (Table S1). Nucleic acid electrophoresis was carried out according to the established protocol [29], with a voltage of 100 V for 20–30 min to separate cDNA and gDNA molecules. Subsequently, the DNA bands were visualized using ultraviolet illumination.

### Quantitative real-time PCR (qRT-PCR)

The H9c2 cells, primary cardiomyocytes, primary fibroblasts, and cardiac tissues underwent Trizol (Vazyme) extraction to isolate total RNA after PBS washing. Subsequently, the obtained total RNA was reverse transcribed using the RT SuperMix for qPCR (+gDNA wiper) kit (Vazyme) following the manufacturer’s instructions. The qRT-PCR was performed using the SYBR qPCR Master Mix kit (Vazyme) and the CFX96 real-time system provided by Bio-Rad. The 2^−ΔΔCt^ method was utilized to quantify the test values, with the internal control GAPDH being employed for comparison purposes. The primer sequences for the circRNAs and mRNAs can be found in Table S1.

### RNase R treatment

The total RNA extracted from H9c2 cells was incubated with RNase R (Epicenter) at 37 °C for 20 min. Each 10 μg of RNA underwent digestion using RNase R at a concentration of 20 U/μL, followed by incubation at 70 °C for 10 min. Subsequently, reverse transcription was performed, followed by qRT-PCR analysis.

### Western blotting

After washing the H9c2 cells and primary cardiomyocytes with a PBS solution, they were lysed on ice using radioimmunoprecipitation assay (RIPA) lysis buffer (Solarbio) containing 0.1 mM phenylmethylsulfonyl fluoride (PMSF, Solarbio) and a cocktail of protease inhibitors (Roche) for 30 min. The protein samples were quantified using the bicinchoninic acid (BCA; Beyotime Biotechnology) concentration measurement and subsequently transferred onto a polyvinylidene fluoride (PVDF) membrane (Millipore) after electrophoresis with 12% sodium dodecyl sulfate polyacrylamide gel electrophoresis (SDS–PAGE). Following this, the PVDF membrane was incubated with 5% skim milk at room temperature for an hour, and then overnight at 4 °C with the primary antibody. After washing the PVDF membrane the next day, secondary antibodies were incubated and protein levels were evaluated using ECL (Solarbio), followed by quantification through ImageJ software. Details of specific antibodies used and their corresponding ratios can be found in Table S3.

### Subcellular fractionation

The H9c2 cells proteins were harvested and subsequently subjected to differential centrifugation using a Nucleocapsid and Cytoplasmic Protein Extraction Kit (Beyotime Biotechnology) to obtain separate fractions of cytoplasmic and nuclear proteins. β-Tubulin was utilized as a reference for evaluating the cytoplasmic protein’s quality, whereas lamin B functioned as an indicator to assess the nuclear protein’s integrity.

### RNA fluorescence in situ hybridization (RNA–FISH)

Upon attaining 70–80% confluence, the H9c2 cells underwent cell fusion. Before the fusion process, PBS was used to cleanse and fix the cells, followed by hybridization as per the guidelines provided by Gene Pharma Company [30]. The sequence of the circCacna1c probe for FISH was 5′-CCCATAGTTGGAACCAGGTTGGAGT-3′.

### Plasmids, siRNAs assays, and cell transfection

The overexpression plasmids for circCacna1c, FTO, and Hnrnpf were synthesized by Gene Pharma Company, and verified by sequencing by Beijing Genomics institution (BGI Genomics Co. Ltd). siRNAs for circCacna1c, FTO, YTHDF2, and Hnrnpf were synthesized by Gene Pharma Company. Additionally, negative controls (NC) were also obtained from the same source. The siRNA sequences are listed in Table S2. The plasmids, siRNA, and NC were transfected into H9c2 cells and primary cardiomyocytes using Lipofectamine 3000 (Invitrogen).

### Cell viability

After washing H9c2 cells and primary cardiomyocytes with PBS, the quantification of cell necroptosis was carried out using a PI kit (Solarbio) in accordance with the manufacturer’s guidelines. The cells were stained with PI and DAPI for 30 min in a light-free environment. Subsequently, the cells underwent a brief rinse before being examined under Leica fluorescence microscope. The percentage of necroptosis cells was determined by dividing the total number of PI-positive nuclei by the total number of DAPI-stained nuclei. An alternative method for identifying cell necroptosis is to measure LDH activity. A total of 20 μL of the cell culture supernatant was collected and LDH activity was measured using a spectrophotometric method kit (Nanjing Jiancheng) according to the manufacturer’s instructions. Cell viability was determined by colorimetric assay using Cell Counting Kit-8 assay (CCK-8; Solarbio). H9c2 cells and primary cardiomyocytes were inoculated onto 96-well plates at a density of 1 × 10^4^ cells per well. Upon treatment, 10 μL of CCK-8 reagent was added and incubated at 37 °C for 1 h. Eventually, the absorbance was measured at 450 nm using an enzyme-labeled instrument (Thermo Fisher Scientific, Inc.). Apoptosis was determined by terminal deoxynucleotidyl transferase dUTP nick end labeling (TUNEL) using the TUNEL Apoptosis Detection kit (Yeasen, Shanghai, China). The testing procedure was conducted in accordance with the kit instructions.

### Methylated RNA immunoprecipitation qPCR (MeRIP–qPCR)

To evaluate the m^6^A levels of circCacna1c, we conducted MeRIP–qPCR following the established protocol with certain modifications (Fig. S3E) [31]. We initially extracted total RNA from H9c2 cells subjected to different treatments and quantified the samples with a NanoDrop ND-1000 instrument (> 500 μg of total RNA). A portion of the RNA sample was retained as an input control. The remaining RNA samples were subjected to overnight incubation with m^6^A-antibody or IgG-antibody-conjugated beads (protein A/G microspheres) in an immunoprecipitation buffer supplemented with RNase inhibitors at 4 °C. RNA samples harboring m^6^A were immuneprecipitated and eluted from the beads. Gene-specific primers were employed for qRT-PCR in both the input control and m^6^A immunoprecipitation samples. The primer sequences for specific genes are provided in Table S1.

### RNA immunoprecipitation (RIP)

The RIP assay was utilized to evaluate the binding between RNA and proteins in H9c2 cells and primary cardiomyocytes [32]. A portion of the RNA sample was retained for input control. Protein A/G microspheres were utilized in combination with different target antibodies and their corresponding IgG antibodies, followed by incubation. The precipitated complexes were then eluted, and total RNA was collected for subsequent analysis using qRT-PCR. To determine the relative enrichment, normalization of the input was performed.

### RNA stability assay

To evaluate the degradation rates of circCacna1c, H9c2 cells were exposed to actinomycin D (10 μg/mL). After specific time intervals (0/2/4/6/12 h), the cells were collected and total RNA was isolated for subsequent qRT-PCR analysis. Subsequently, the relative decay rates of circCacna1c compared with the initial 0 h timepoint were determined.

### RNA pulldown and liquid chromatography/mass spectroscopy (LC–MS/MS) analysis

To detect and analyze the proteins bound to circCacna1c, a biotin-labeled circCacna1c and NC binding site probe was synthesized via BGI Genomics. The sequence of the circCacna1c probe was as follows: 5′-TCCCATAGTTGGAACCAGGTTGGAGTTGGT-3′. The sequence of the NC probe was as follows: 5′-ACCAACTCCAACCTGGTTCCAACTATGGGA-3′. To begin with, a probe, streptavidin magnetic beads, and cell lysates were co-incubated to perform an RNA pulldown experiment [33]. After performing RNA pulldown, the protein samples were collected and resolved by SDS–PAGE with a concentration of 12.5%, and the gel was processed by Shanghai Applied Protein Technology Company for MS. The protein-peptide samples in the gel were digested with endonucleases (enzyme = trypsin; missed cleavage = 2) and analyzed with liquid chromatography tandem mass spectrometry (LC–MS/MS; nanoLC-QE), MASCOT, and other mass-spectral matching software to obtain qualitative identification of the target protein-peptide molecules. The detection of fixed modifications was also performed [34]. The process of enzyme digestion, mass spectrometry analysis, and database searches was conducted by Shanghai Applied Protein Technology Company. The process of mass spectrometry and all the data are presented in Fig. S5A and Table S4.

### Enrichment analysis of the Gene Ontology (GO)

We performed a GO biological process (BP) analysis on the proteins enriched in MS to uncover the potential functions of circCacna1c binding proteins. Hypergeometric tests were utilized to identify significantly enriched GO terms among differentially expressed transcripts, and *P* values were adjusted for multiple testing using false discovery rate (FDR) correction methods. The *Q* value threshold of 0.05 was applied to determine noteworthy enrichment, and the outcomes were visualized using https://www.bioinformatics.com.cn [35, 36].

### Mouse model of MI injury

To investigate the impact of circCacna1c on necroptosis in cardiomyocytes in vivo, the MI injury model was established in an 8-week-old male adult C57BL/6 mouse by permanent ligation of the left anterior descending artery (LAD). The overexpression vector for circCacna1c was constructed using adeno-associated virus (serotype: AAV9), which was synthesized by Obio. Cardiomyocytes of the mouse were infected with AAV9-circCana1c (4 × 10^12^ v.g./mL) through tail vein injection (approximately 100 μL per mouse) 3 weeks prior to LAD surgery (Fig. 7D). After the LAD artery, mouse received an intravenous injection of PI (Sigma) at a dosage of 10 mg/kg to mark necroptosis cells in the heart. The hearts were frozen sectioned in 5 μm thickness and the necroptosis cells were quantified after DAPI re-staining. The actinin (Sigma) was used to identify myocardial cells, and fluorescence microscopy (Olympus) was employed for observing and determining the rate of myocardial cell necroptosis.

To quantify the extent of myocardial infarction, the heart was surgically removed under anesthesia 24 h after occlusion of the LAD and rapidly frozen at a temperature of −20 °C for a duration of 20 min. Subsequently, transverse sections were made from the heart and immersed in a solution containing 1.5% concentration of 2,3,5-triphenyltetrachloride (TTC; Sigma) for a period of 20 min to visualize the area affected by infarction. The ratio between the infarcted region and total area was determined using ImageJ software.

### Cardiac functional assays

After a 2-week period of maintenance following MI, the impact of circCacna1c on cardiac dysfunction in MI was evaluated. The contraction ability of the left ventricle (LVFS), the efficiency of blood pumping from the left ventricle (LVEF), the dimensions of the left ventricular inner chamber (LVIDs), and the left ventricular internal dimension in diastole (LVIDd) were measured using a small animal ultrasound device manufactured by Thermo Scientific while the mouse was under anesthesia.

### Statistics and reproducibility

In vitro findings were reported as the mean ± standard error of the mean (SEM) deviation derived from a minimum of three independent experiments. In vivo results were presented as the mean ± SEM deviation obtained from at least six independent experiments. All data were analyzed using GraphPad Prism software (GraphPad). Specifically, *t*-tests were employed to determine statistical significance between two groups, while one-way analysis of variance (ANOVA) and Tukey’s multiple comparison test were utilized for comparisons among multiple groups. A *P* value less than 0.05 was considered statistically significant (*P* < 0.05).

## Results

### circCacna1c is abundant in cardiomyocytes

**Fig. 1:**
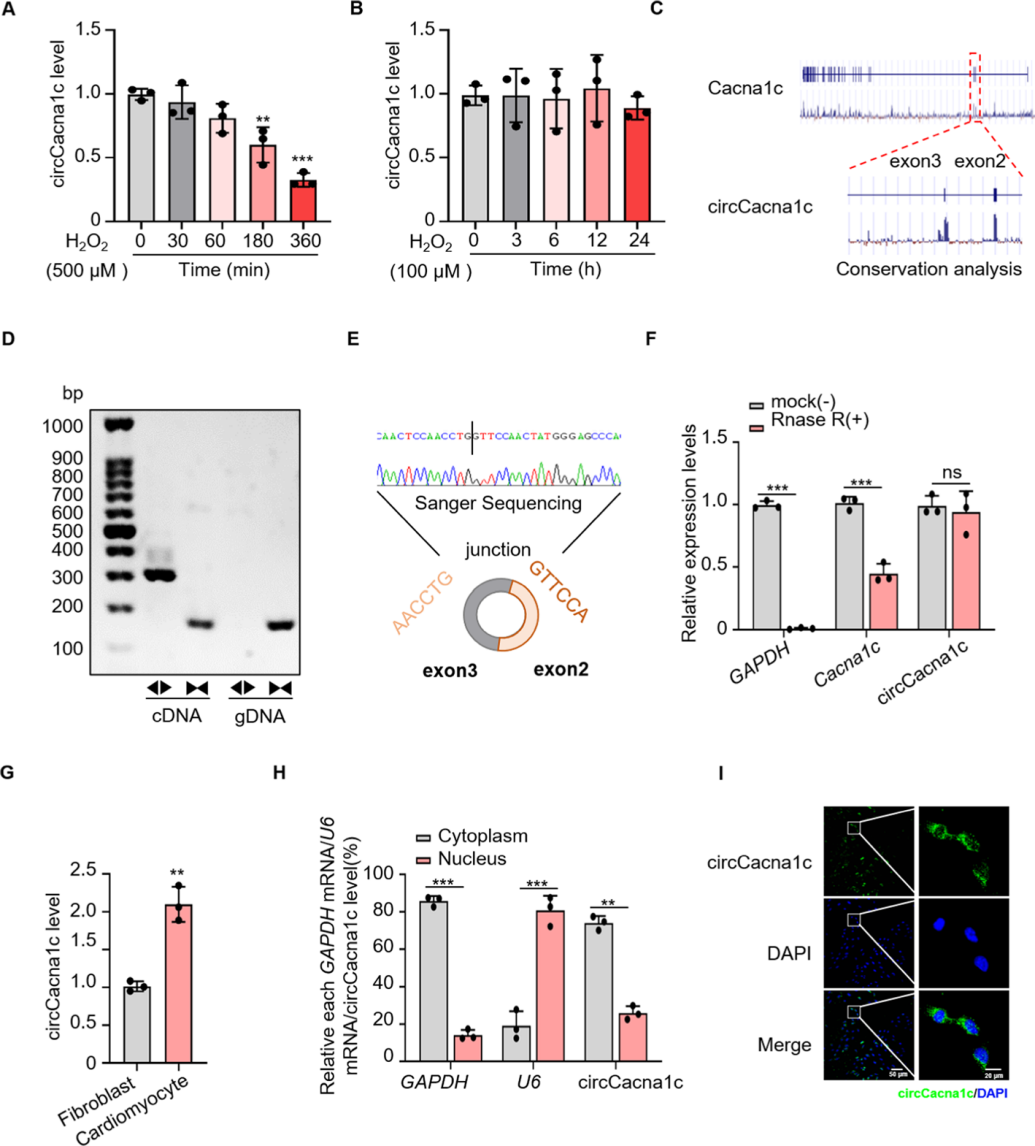
Verification of circCacna1c. **A**, **B** H9c2 cells were treated with H_2_O_2_ (500 μΜ or 100 μΜ), and the expression level of circCacna1c was determined using qRT-PCR with GAPDH mRNA as the internal reference gene for normalization. **P < 0.01, ***P < 0.001 versus 0 h, ns > 0.05. *n* = 3. **C** PhyloP sequence analysis was conducted on the conserved circCacna1c derived from exons 2 and 3 of the Cacna1c gene. **D** Divergent primers (◄►) and convergent primers (►◄) were utilized to amplify cDNA and gDNA samples from H9c2 cells for examining circCacna1c. **E** Sanger sequencing was employed to confirm the junction between the head and tail of circCacna1c. **F** RNAs from H9c2 cells were incubated with either RNase R (+) or mock buffer alone (−). Following digestion, the purified RNAs were analyzed for the expression levels of *GAPDH* mRNA, *Cacna1c* mRNA, and circCacna1c using qRT-PCR. ***P < 0.001 versus mock (−). *n* = 3. **G** The qRT-PCR technique was used to analyze the expression levels of circCacna1c in both cardiomyocytes and cardiac fibroblasts derived from neonatal rats. **P < 0.01 versus cardiomyocytes. *n* = 3. **H** Cellular components of H9c2 cells, including cytoplasmic and nuclear fractions, were separated for subsequent qRT-PCR analysis to determine the relative proportions of GAPDH mRNA, U6, and circCacna1c. **P < 0.01, ***P < 0.001. *n* = 3. **I** The intracellular distribution of circCacna1c in H9c2 cells was examined using RNA-FISH. The presence of circCacna1c was visualized with specific probes indicated by the green signal. Nuclei stained with DAPI are represented in blue. Scale bars, 50 μm and 20 μm

circRNAs play crucial roles in regulating various forms of programmed cell death during the pathogenesis of different CVDs, including MI [37]. To identify and characterize functional circRNAs associated with necroptosis in cardiomyocytes, we identified five circRNAs with high expression levels and evolutionary conservation in cardiac tissue by utilizing an online database in conjunction with published datasets [38–40]. In our previous study, we have demonstrated that high concentrations of H_2_O_2_ (500 μM) predominantly induce necroptosis in cardiomyocytes, whereas lower concentrations (100 μM) elicit apoptosis [27]. Subsequently, the qRT-PCR analysis was conducted to validate the expression levels of selected circRNAs in H9c2 cells treated with varying concentrations of H_2_O_2_. Among them, the expression level of circCacna1c was significantly downregulated in H9c2 cells after treatment with 500 μM H_2_O_2_ (Supplementary Fig. 1), whereas no significant difference was observed following treatment with 100 μM H_2_O_2_ at different time points (Fig. 1A, B). Additionally, circCacna1c exhibited a high degree of conservation (Fig. 1C and Fig. S1B). Based on these findings, circCacna1c was selected for further investigation. The circular feature of circCacna1c was confirmed through PCR analysis using convergent and divergent primers. Specifically, the utilization of divergent primers successfully amplified circCacna1c from cDNA while no amplification was observed from genomic DNA (Fig. 1D). Moreover, Sanger sequencing technology validated a specific back splice junction in circCacna1c (Fig. 1E). After treatment with RNase R, the stability of circCacna1c was assessed. We observed that the linear RNA transcripts of *Cacna1c* and *GAPDH* mRNA showed significant degradation, whereas circCacna1c remained stable (Fig. 1F). This finding provides compelling evidence for the circular feature of circCacna1c. The expression levels of circCacna1c were compared between neonatal rat cardiomyocytes and cardiac fibroblasts. As shown in Fig. 1G, under physiological conditions, circCacna1c exhibited significantly higher expression in cardiomyocytes than in cardiac fibroblasts. Moreover, circCacna1c was mainly found in the cytoplasm with a considerable level also detected in the nucleus (Fig. 1H, I). Taken together, these findings suggest that circCacna1c is abundantly expressed in cardiomyocytes under physiological conditions, but its expression is downregulated during necroptosis.

### circCacna1c inhibits H9c2 cell necroptosis

**Fig. 2:**
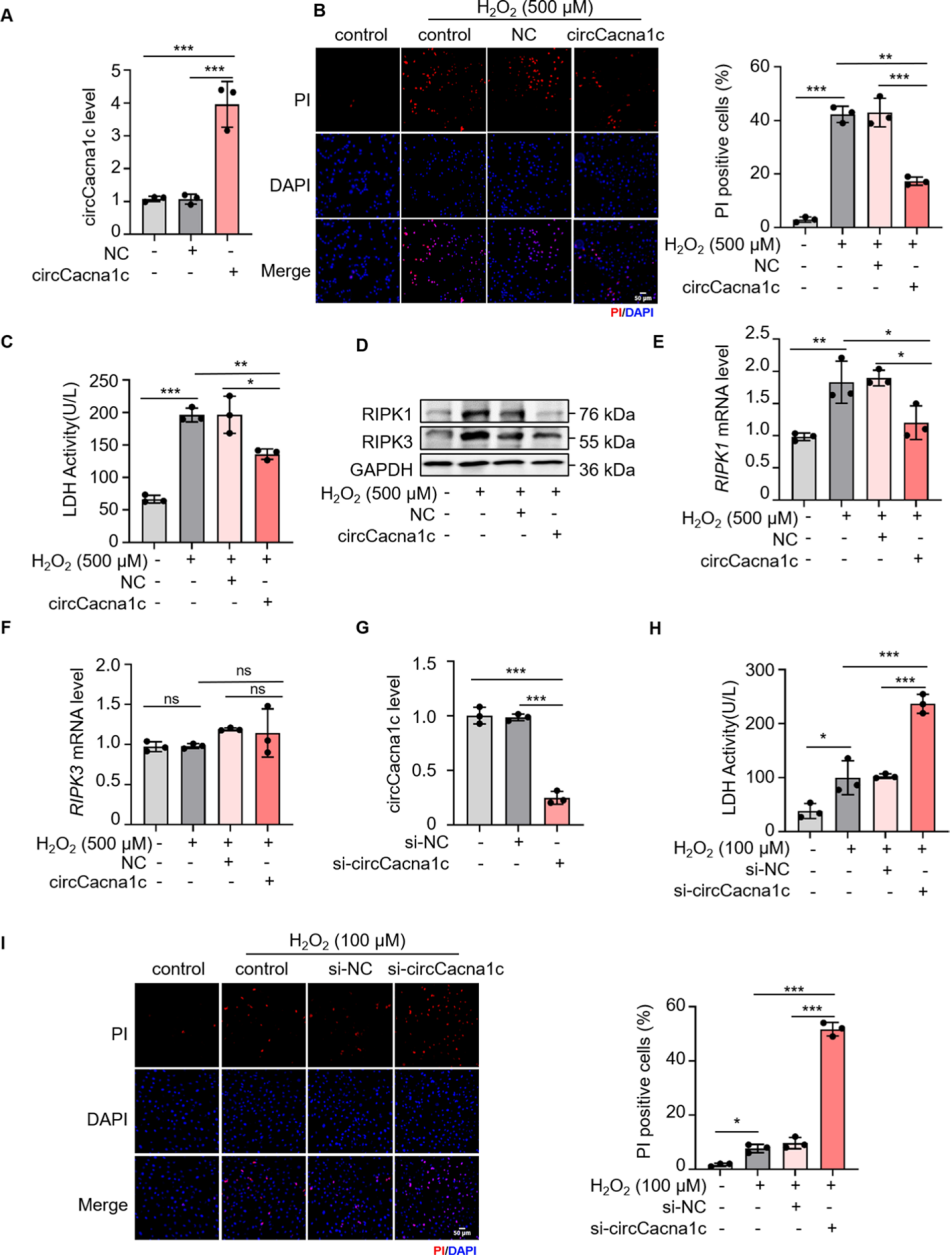
circCacna1c inhibits H_2_O_2_-induced necroptosis. **A** H9c2 cells were transfected with the circCacna1c expression vector, and the expression level of circCacna1c was analyzed by qRT-PCR. The empty vector was used as a NC (empty vector). ***P < 0.001. *n* = 3. **B**, **C** The impact of circCacna1c on necroptosis in H9c2 cells was assessed through experiments detecting the rate of PI-positive cells and the activity of LDH. **B** A representative image is displayed on the left side, while the calculated rates of necroptosis from three independent experiments are shown on the right side. Red indicates PI-positive nuclei, while blue represents DAPI stained nuclei. Scale bars, 50 μm. **P < 0.01, ***P < 0.001. *n* = 3. **C** The level of LDH in the cell supernatant was measured. *P < 0.05, **P < 0.01, ***P < 0.001. *n* = 3. **D** The protein levels of RIPK1 and RIPK3 were quantified in H9c2 cells, with GAPDH selected as a reference. *n* = 3. **E**, **F** The mRNA levels of *RIPK1* and *RIPK3* were also quantified in H9c2 cells. *P < 0.05, **P < 0.01, ns > 0.05. *n* = 3. **G** Following transfection of circCacna1c siRNA (si-circCacna1c) into H9c2 cells, the expression level of circCacna1c was assessed using qRT-PCR. si-NC: negative control siRNA. ***P < 0.001 versus si-NC. *n* = 3. **H**, **I** Subsequent experiments were conducted to evaluate the impact of si-circCacna1con necroptosis in H9c2 cells through detection of the rate of PI positive cells and LDH activity. **H** The level of LDH in the cell supernatant was measured. *P < 0.05, ***P < 0.001. *n* = 3. **I** A representative image is displayed on the left side, while the calculated rates of necroptosis from three independent experiments are shown on the right side. Red indicates PI-positive nuclei, while blue represents DAPI stained nuclei. Scale bars, 50 μm. *P < 0.05, ***P < 0.001. *n* = 3

The extensive loss of cardiomyocytes resulting from necroptosis is a fundamental pathological mechanism underlying MI [37]. The observed downregulation of circCacna1c in H9c2 cells treated with 500 μM H_2_O_2_ suggests the potential involvement of circCacna1c in the regulation of cardiomyocyte necroptosis. To investigate this hypothesis, a series of validation assays were conducted using gain-of-function and loss-of-function experiments (Table S2). Overexpression of circCacna1c markedly reduced the proportion of PI positive cells, suppressed LDH activity, and prevented the decline in cell viability in H9c2 cells treated with 500 μM H_2_O_2_ (Fig. 2A–C; Fig. S2A, B). Corresponding functional tests were carried out in primary cardiomyocytes. During the necroptosis of primary cardiomyocytes, circCacna1c decreases (Fig. S2C), and overexpression of circCacna1c can inhibit the increase of PI-positive cells and LDH induced by H_2_O_2_, and restore certain cell viability (Fig. S2D–G). Subsequently, we examined the impact of circCacna1c on the expression levels of RIPK1 and RIPK3 in H9c2 cells treated with 500 μM H_2_O_2_. As shown in Fig. 2D–F (Fig. S2H, I), overexpression of cirCacna1c significantly inhibited PIPK1 expression at both protein and mRNA levels, while only attenuating RIPK3 protein level without affecting its mRNA level. TUNEL staining indicated that the reduction of circCacna1c was not significantly correlated with cardiomyocyte apoptosis (Fig. S2J). These findings strongly support that circCacna1c effectively inhibits necroptosis in cardiomyocytes. Next, we assessed the effect of circCacna1c silencing on the sensitivity of H9c2 cells to H_2_O_2_. Knockdown of cirCacna1c significant enhanced the activity of LDH, increased the number of PI positive cells, and decreased cell viability (Fig. 2G–I; Fig. S2K, L). These results further confirm that cirCacna1c can block cardiomyocyte necroptosis.

### FTO-mediated dmethylation of m^6^A modification contributes to the degradation of circCacna1c

**Fig. 3:**
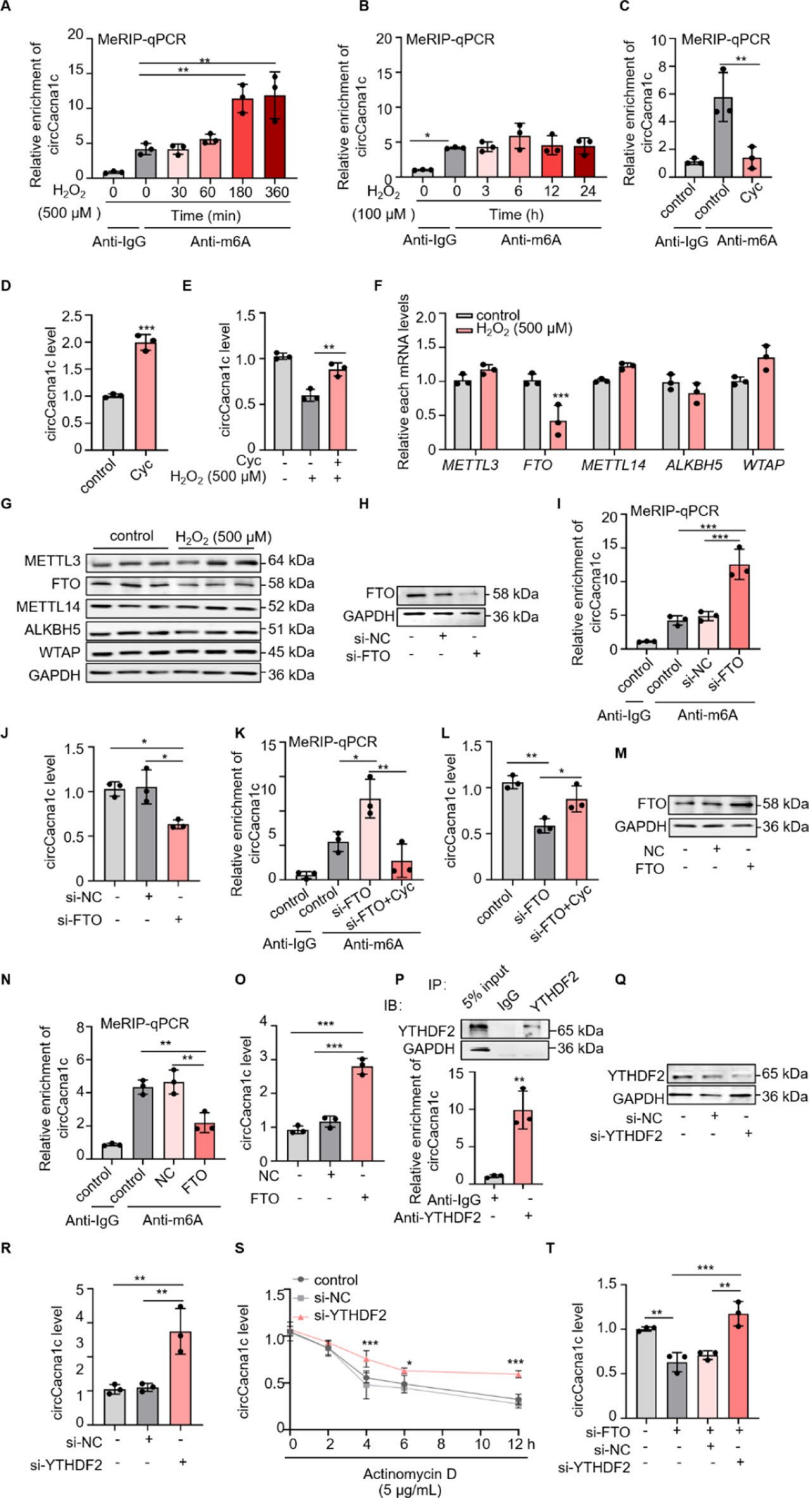
circCacna1c is regulated by FTO-mediated m^6^A methylation. **A**, **B** H9c2 cells were treated with H_2_O_2_ (500 μΜ or 100 μΜ). MeRIP–qPCR analysis was conducted to assess the m^6^A modification level of circCacna1c. **P < 0.01 versus control anti-m^6^A. *P < 0.05, **P < 0.01. *n* = 3. **C** The cycloleucine (Cyc) is a specific inhibitor of *S*-adenosylmethionine-mediated methylation (40 mM; 24 h). H9c2 cells were treated with Cyc for 24 h. MeRIP–qPCR analysis of m^6^A modification level of circCacna1c. **P < 0.01. *n* = 3. **D** H9c2 cells were treated with Cyc. The expression level of circCacna1c was analyzed by qRT-PCR. ***P < 0.001. *n* = 3. **E** The H9c2 cells were subjected to Cyc treatment followed by exposure to 500 μM H_2_O_2_ for a duration of 6 h. Subsequently, the expression level of circCacna1c was assessed using qRT-PCR analysis. **P < 0.01. *n* = 3. **F**, **G** After exposing H9c2 cells to a concentration of 500 μM H_2_O_2_ for a duration of 6 h, the cells were collected and subsequently analyzed for mRNA levels of **F** *METTL3*, *FTO*, *METTL14*, *ALKBH5*, and *WTAP*, as well as protein levels of **G** METTL3, FTO, METTL14, ALKBH5, and WTAP. GAPDH was selected as a reference. ***P < 0.001. *n* = 3. **H** The FTO siRNA (si-FTO) was transfected into H9c2 cells, followed by the determination of FTO protein levels. GAPDH was selected as a reference. *n* = 3. **I** MeRIP–qPCR analysis of m^6^A modification level of circCacna1c. ***P < 0.001. *n* = 3. **J** The expression level of circCacna1c was analyzed by qRT-PCR. *P < 0.05. *n* = 3. **K** MeRIP–qPCR analysis of m^6^A modification level of circCacna1c. *P < 0.05, **P < 0.01. *n* = 3. **L** The expression level of circCacna1c was analyzed by qRT-PCR. *P < 0.05, **P < 0.01. *n* = 3. **M** The H9c2 cells were transfected with a plasmid that overexpressed FTO, followed by the determination of FTO protein levels. GAPDH was selected as a reference. *n* = 3. **N** MeRIP–qPCR analysis of m^6^A modification level of circCacna1c. **P < 0.01. *n* = 3. **O** The expression level of circCacna1c was analyzed by qRT-PCR. ***P < 0.001. *n* = 3. **P** The YTHDF2 antibody was utilized in the RIP assay to determine its affinity for circCacna1c. Subsequently, western blotting analysis was performed to confirm the binding specificity of the YTHDF2 antibody, and qRT-PCR was employed to assess the level of circCacna1c enrichment facilitated by the YTHDF2 antibody in H9c2 cells. **P < 0.01. *n* = 3. **Q** The YTHDF2 siRNA (si-YTHDF2) was transfected into H9c2 cells, followed by the determination of YTHDF2 protein levels. *n* = 3. **R** The expression level of circCacna1c was analyzed by qRT-PCR. **P < 0.01. *n* = 3. **S** The circCacna1c expression levels in H9c2 cells were analyzed using qRT-PCR after subjecting them to actinomycin D treatment for varying durations. *P < 0.05, ***P < 0.001. *n* = 3. **T** The expression level of circCacna1c was analyzed by qRT-PCR. **P < 0.01, ***P < 0.001. *n* = 3

Given the downregulation of circCacna1c in H9c2 cells treated with 500 μM H_2_O_2_, it can be inferred that circCacna1c undergoes degradation during necroptosis. The m^6^A modification has been demonstrated to decrease the stability of circRNAs [24]. Subsequently, we investigated whether m^6^A modification is involved in regulating the degradation of circCacna1c. Bioinformatics analysis revealed the presence of multiple m^6^A modification sites within the sequence of circCacna1c (Fig. S3A–D). This finding was further validated in H9c2 cells using MeRIP–qPCR (Fig. S3E). Next, we examined the m^6^A modification in circCacna1c was examined in H9c2 cells exposed to varying concentrations of H_2_O_2_. As shown in Fig. 3A, B, treatment of 500 μM H_2_O_2_ significantly induced a time-dependent increase in the levels of m^6^A modification within circCacna1c, whereas no significant difference was observed in cells treated with 100 μM H_2_O_2_. When Cycloleucine (Cyc), an m^6^A inhibitor, was administered to H9c2 cells, it led to a significant decrease in levels of m^6^A modification on circCacna1c and a remarkable upregulation of circCacna1c expression (Fig. 3C, D). Interestingly, Cyc administration effectively restored decreased expression levels of circCacna1c induced by exposure to 500 μM H₂O₂ (Fig. 3E). These findings strongly suggest that m^6^A modification plays a vital role in necroptosis by facilitating the degradation of circCacna1c.

The m^6^A modification is a dynamic process regulated by methyltransferase (writer) and demethylase (eraser) [22]. We subsequently identified the specific enzymes responsible for mediating m^6^A modification in circCacna1c. Western blotting and qRT-PCR analysis demonstrated a significant downregulation of FTO protein and mRNA levels in H9c2 cells treated with 500 μM H_2_O_2_ (Fig. 3F, G; Fig. S3F), suggesting the involvement of FTO in the demethylation of circCacna1c m^6^A modification during necroptosis. Subsequently, FTO was silenced in H9c2 cells using a specific small interfering RNA targeting FTO (si-FTO) (Fig. 3H; Fig. S3G). As shown in Fig. 3I, J, knockdown of FTO significantly augmented the levels of m^6^A modification within circCacna1c while concurrently reducing its expression level. Remarkably, treatment with Cyc effectively counteracted the elevated m^6^A modification levels induced by FTO knockdown and restored circCacna1c expression to normal levels (Fig. 3K, L). Consistent with this, overexpression of FTO obtained the opposite effect (Fig. 3M–O; Fig. S3H). Collectively, these results showed that FTO modulated the stability of circCacna1c by mediating the demethylation process of m^6^A modification during necroptosis.

It has been reported that m^6^A modification reduced the stability of circRNAs in an YTHDF2-dependent manner [24, 41]. Next, we investigated whether YTHDF2 is involved in FTO-mediated demethylation of circCacna1c m^6^A modification. RIP assay revealed the physiological interaction between YTHDF2 and circCacna1c in H9c2 cells (Fig. 3P). This interaction was likewise present in primary cardiomyocytes (Fig. S3I). Then, specific small interfering RNA targeting YTHDF2 (si-YTHDF2) was utilized to knock down the expression of YTHDF2. As shown in Fig. 3Q (Fig. S3J) and R, silencing YTHDF2 significantly upregulated the expression levels of circCacna1c. Consistently, silencing YTHDF2 effectively retards the degradation of circCacna1c induced by actinomycin D, an inhibitor of transcription (Fig. 3S). Furthermore, silencing YTHDF2 also reverse the downregulation of circCacna1c caused by FTO knockdown (Fig. 3T). Taken together, these data reveal that FTO-mediated m^6^A demethylation effectively inhibits the degradation of circCacna1c in a YTHDF2 dependent manner.

### Role of FTO and circCacna1c in necroptosis

**Fig. 4:**
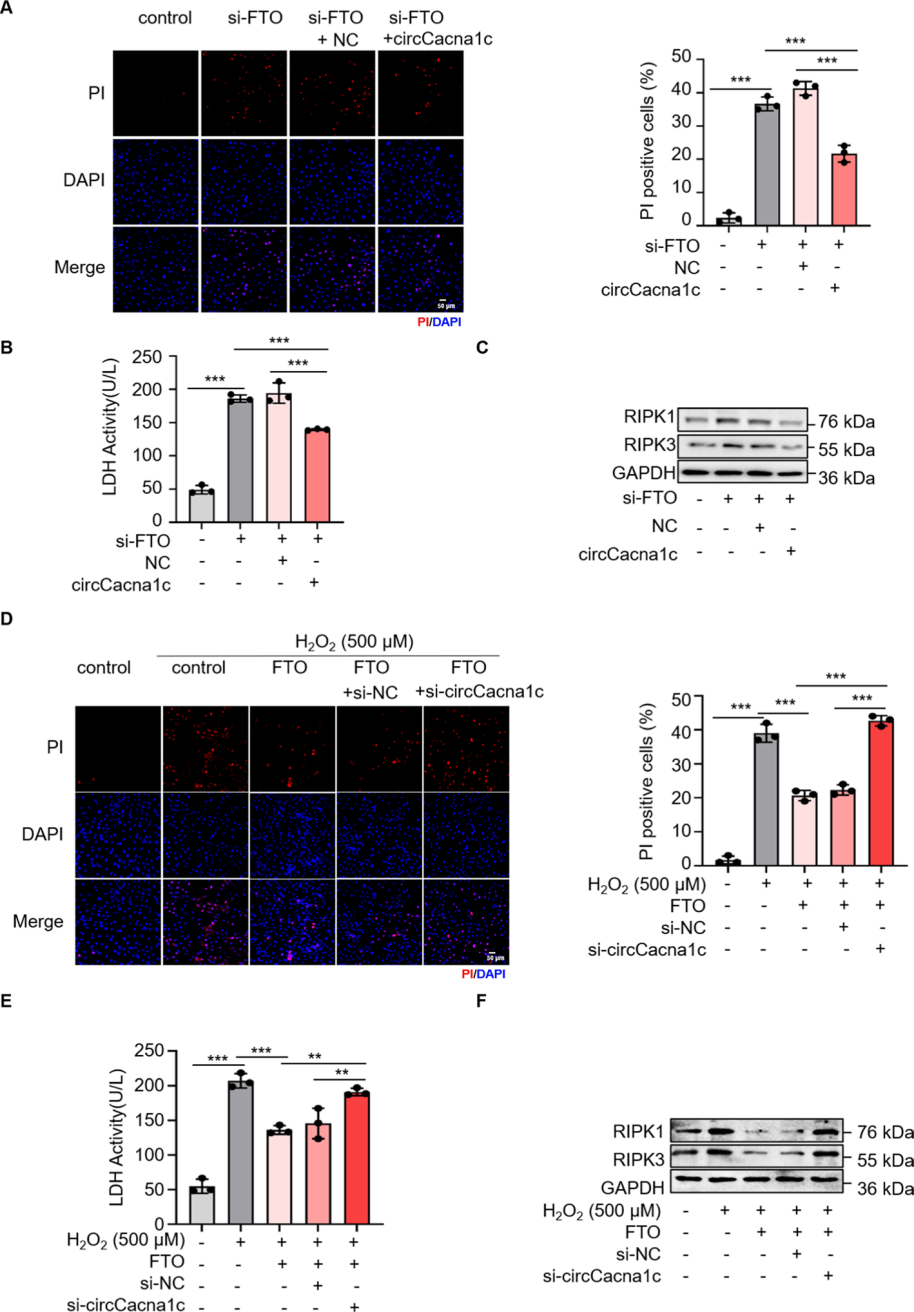
FTO is involved in the regulation of necroptosis by regulating the m^6^A modification of circCacna1c. **A**–**C** H9c2 cells were transfected with si-FTO and circCacna1c expression vectors. The PI assay (**A**) and LDH activity assay (**B**) were employed to assess cell necroptosis in H9c2 cells. ***P < 0.001. *n* = 3. **C** The protein levels of RIPK1 and RIPK3 were determined. GAPDH was selected as a reference. *n* = 3. **D**–**F** H9c2 cells exposed to 500 μM H_2_O_2_ for 6 h were transfected with FTO expression vector and si-circCacna1c. The cell necroptosis in H9c2 cells was evaluated using the PI assay (**D**) and LDH activity assay (**E**). **P < 0.01, ***P < 0.001. *n* = 3. **F** The protein levels of RIPK1 and RIPK3 were analyzed. GAPDH was selected as a reference. *n* = 3

To investigate the functional significance of FTO-mediated m^6^A demethylation of circCacna1c in necroptosis, we utilized H9c2 cells as a model and performed PI staining, LDH activity assay, and cell activity assay. Knockdown of FTO significantly increased the population of PI-positive cells and attenuated LDH activity in H9c2 cells, whereas overexpression of cirCacna1c mitigated these effects induced by FTO silencing (Fig. 4A, B; Fig. S4A). Moreover, cirCacna1c overexpression downregulated the upregulated expression levels of RIPK1 and RIPK3 caused by FTO knockdown (Fig. 4C; Fig. S4B, C). Subsequently, we confirmed the impact of FTO and cirCacna1c on necroptosis using PI staining and LDH activity analysis in H9c2 cells treated with 500 μM H_2_O_2_. Knockdown of circCacna1c intensified the decrease in the number of PI-positive cells and lowered LDH activity induced by FTO overexpression, while cirCacna1c knockdown reinstated the reduced expression levels of RIPK1 and RIPK3 triggered by FTO overexpression (Fig. 4D–F; Fig. S4D–F). In summary, these findings suggest that FTO exerts a suppressive effect on cardiomyocyte necroptosis through its involvement in m^6^A demethylation processing affecting circCacna1c.

### circCacna1c inhibits the translocation of Hnrnpf into the nucleus

**Fig. 5:**
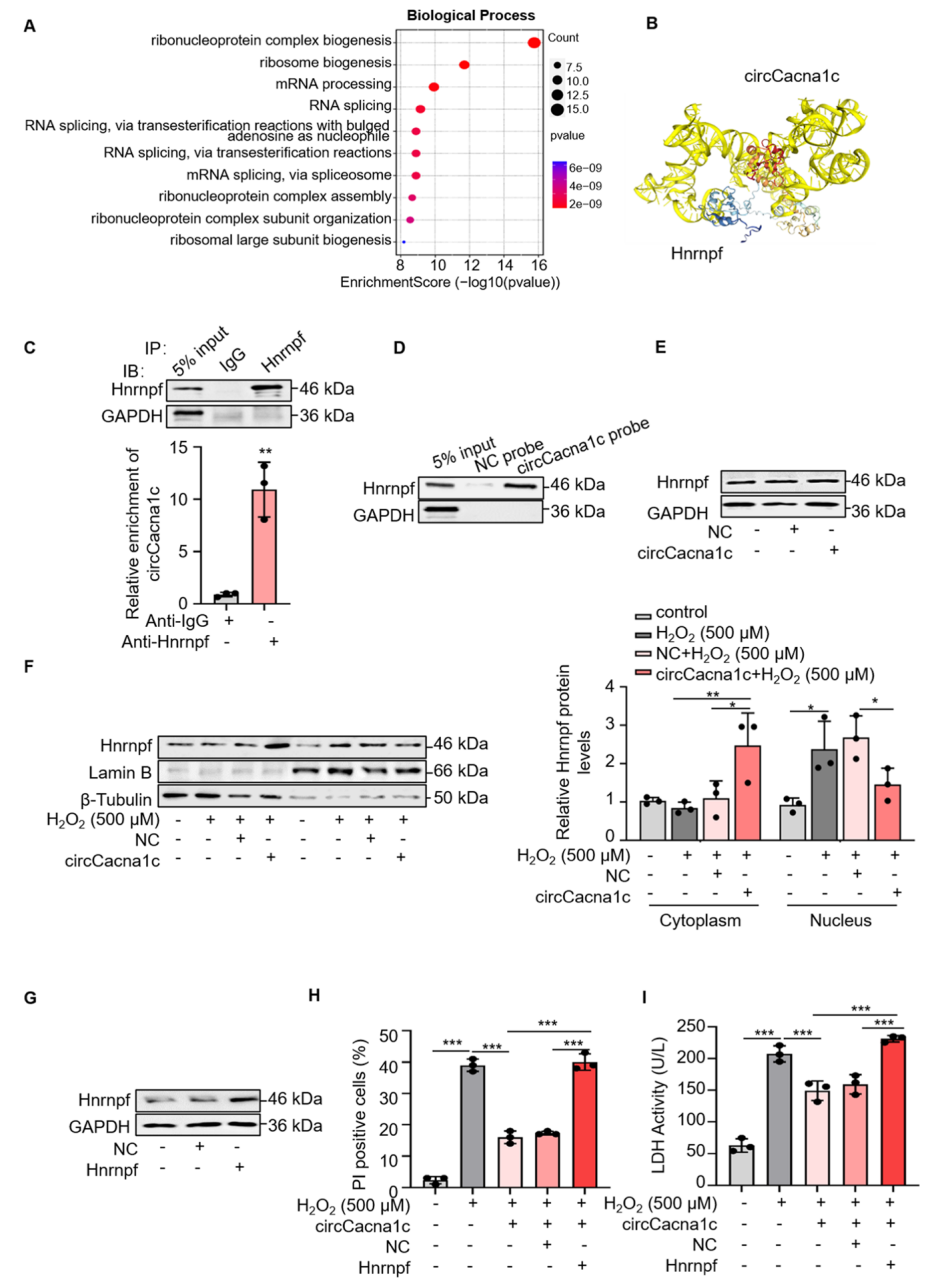
circCacna1c binds to Hnrnpf and inhibits nuclear translocation of Hnrnpf. **A** Gene Ontology (GO) analysis of proteins captured by the circCacna1c probe. **B** Prediction of binding of circCacna1c to Hnrnpf. **C** The Hnrnpf antibody was utilized in the RIP assay to determine its affinity for circCacna1c. Subsequently, western blotting analysis was performed to confirm the binding specificity of the Hnrnpf antibody, and qRT-PCR was employed to assess the level of circCacna1c enrichment facilitated by the Hnrnpf antibody in H9c2 cells. **P < 0.01. *n* = 3. **D** RNA pulldown was used to detect the binding of Hnrnpf to circCacna1c. *n* = 3. **E** H9c2 cells were transfected with the circCacna1c expression vector, followed by the determination of Hnrnpf protein levels. *n* = 3. **F** The circCacna1c expression vector was transfected into H9c2 cells, which were then exposed to 500 μM H_2_O_2_ for a period of 6 h. Following this, the cytoplasmic and nuclear fractions were separated and the protein quantity of Hnrnpf was measured. Lamin B and β-tubulin acted as controls for nuclear and cytoplasmic compartments respectively, with their relative protein levels being determined. *P < 0.05, **P < 0.01. *n* = 3. **G** The H9c2 cells were transfected with a plasmid that overexpressed Hnrnpf, followed by the determination of Hnrnpf protein levels. GAPDH was selected as a reference. *n* = 3. **H**, **I** H9c2 cells were transfected with circCacna1c expression vectors and Hnrnpf expression vectors, then exposed to 500 μM H_2_O_2_ for a period of 6 h. The PI assay (**H**) and LDH activity assay (**I**) were employed to assess cell necroptosis in H9c2 cells. ***P < 0.001. *n* = 3

To elucidate the molecular mechanism underlying circCacna1c-mediated protection against cardiomyocyte necroptosis, we first performed an RNA pull-down assay to screen the interacting proteins of circCacna1c in cardiomyocytes (Fig. S5A), followed by subsequent MS analysis. In total, we identified 40 proteins that potentially interacted with circCacna1c in H9c2 cells (Fig. S5B, Table S4). Gene Ontology (GO) enrichment analysis revealed that the 40 proteins were primarily involved in RNA splicing, mRNA processing, ribosome biogenesis, and ribonucleoprotein complex biogenesis (Fig. 5A). Heterogeneous ribonucleoproteins F (Hnrnpf) plays an important role in the regulation of gene expression, including RNA-specific exon skipping events, selective splicing, and mRNA stability (Fig. S5C) [42, 43]. PTBP1 is capable of post-transcriptional regulation of *RIPK1* mRNA in the necroptosis [44]. Additionally, a protein interaction network analysis using the STRING database (https://cn.string-db.org/) revealed that Hnrnpf can interact with PTBP1. This suggests that Hnrnpf may be an interacting protein of circCacna1c and participate in the role of circCacna1c in the necroptosis. The HDOCK server predicted a potential interaction between circCacna1c and Hnrnpf (Fig. 5B). Subsequently, RIP and RNA pulldown assays were performed to validate the direct binding of circCacna1c to Hnrnpf (Fig. 5C, D). This direct binding is also stably existing in primary cardiomyocytes (Fig. S5D, E). Furthermore, we examined the effect of circCacna1c on the expression of Hnrnpf. As shown in Fig. 5E (Fig. S5F), ectopic expression of circCacna1c in H9c2 cells did not exert any discernible effect on the levels of Hnrnpf. It has been reported that Hnrnpf distributes in both the nucleus and cytoplasm and undergoes nuclear translocation under hypoxic conditions [45]. In line with previous findings, treatment of H9c2 cells with 500 μΜ H_2_O_2_ led to the translocation of Hnrnpf from the cytoplasm to the nucleus (Fig. S5G, H). This effect was counteracted by the overexpression of circCacna1c (Fig. 5F). The subsequent investigation focused on the impact of Hnrnpf and cirCacna1c on necroptosis. Overexpression of circCacna1c in H9c2 cells led to a decrease in the number of PI-positive cells and LDH activity induced by 500 μM H_2_O_2_. Conversely, overexpression of Hnrnpf reversed these effects caused by circCacna1c overexpression (Fig. S5I–O; Fig. 5G–I). These findings indicate that circCacna1c shields against cardiomyocyte necroptosis by inhibiting the nuclear translocation of Hnrnpf.

### circCacna1c inhibits the expression of RIPK1 by competitively binding to Hnrnpf

**Fig. 6:**
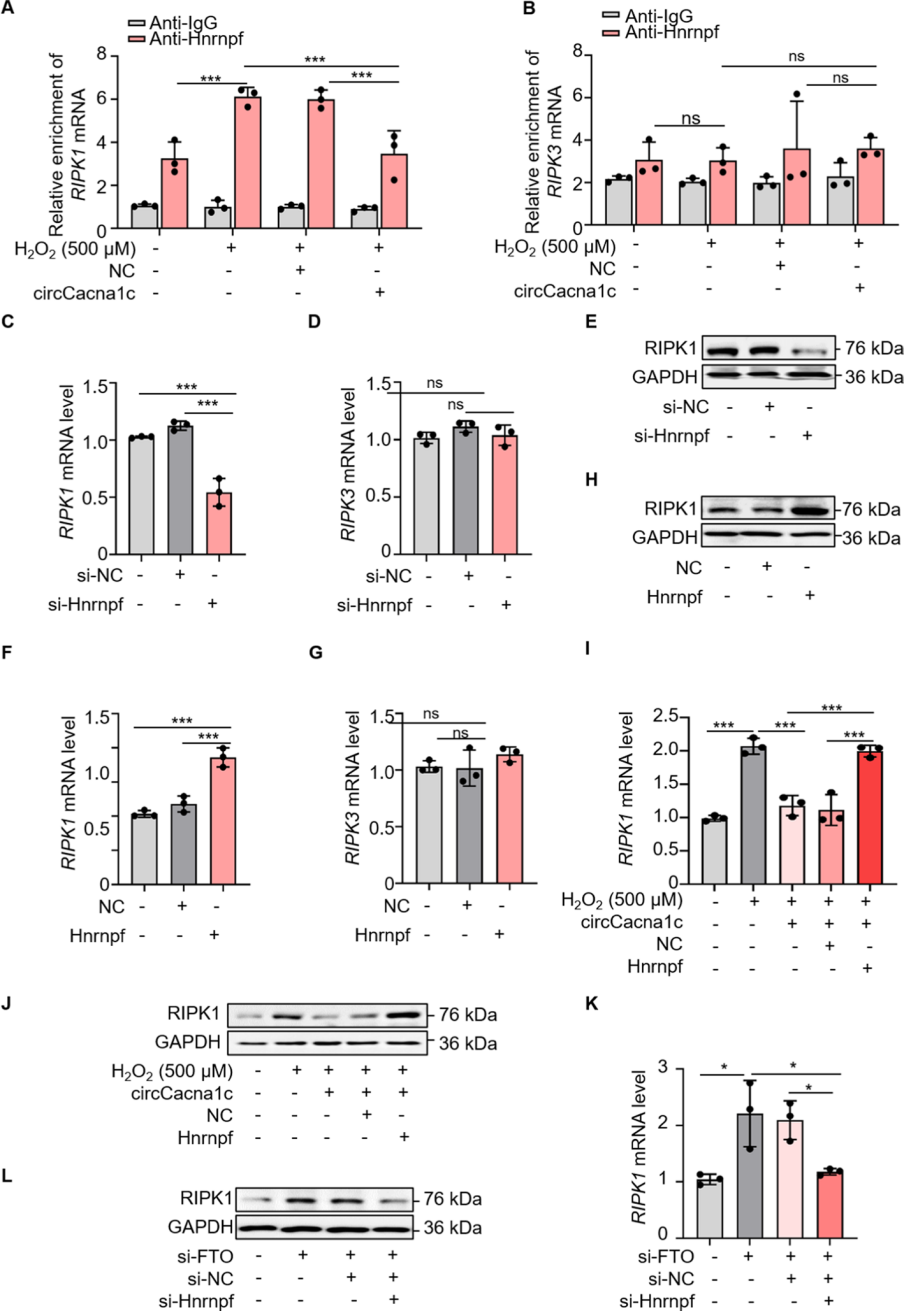
circCacna1c inhibits RIPK1 expression by interacting with Hnrnpf. **A**, **B** The Hnrnpf antibody was utilized in the RIP assay to determine its affinity for *RIPK1* mRNA or *RIPK3* mRNA. The qRT-PCR was employed to assess the level of *RIPK1* mRNA or *RIPK3* mRNA enrichment facilitated by the Hnrnpf antibody in H9c2 cells. ***P < 0.001, not significant (ns) > 0.05. *n* = 3. **C**–**E** The Hnrnpf siRNA (si-Hnrnpf) was transfected into H9c2 cells. The purpose of cell collection was to assess the mRNA expression levels of *RIPK1* (**C**) and *RIPK3* (**D**), along the protein levels of RIPK1 (**E**). GAPDH was selected as a reference. ***P < 0.001, ns > 0.05. *n* = 3. **F**–**H** The H9c2 cells were transfected with a plasmid that overexpressed Hnrnpf. The purpose of cell collection was to assess the mRNA expression levels of *RIPK1* (**F**) and *RIPK3* (**G**), along the protein levels of RIPK1 (**H**). GAPDH was selected as a reference. ***P < 0.001, ns > 0.05. *n* = 3. **I**, **J** H9c2 cells were transfected with circCacna1c expression vectors and Hnrnpf expression vectors, then exposed to 500 μM H_2_O_2_ for a period of 6 h. The purpose of cell collection was to assess the mRNA expression levels of *RIPK1* (**I**), along the protein levels of RIPK1 (**J**). GAPDH was selected as a reference. ***P < 0.001. *n* = 3. **K**, **L** H9c2 cells were transfected with si-FTO and si-Hnrnpf. The purpose of cell collection was to assess the mRNA expression levels of *RIPK1* (**K**), along the protein levels of RIPK1 (**L**). GAPDH was selected as a reference. *P < 0.05. *n* = 3

RIPK1 and RIPK3 are crucial kinases involved in the process of necroptosis [10]. Given Hnrnpf’s demonstrated role in RNA splicing and mRNA processing, we subsequently investigated whether circCacna1c and Hnrnpf could regulate the expression of RIPK1 and RIPK3 in cardiomyocytes. The RIP assay revealed direct binding of Hnrnpf to *RIPK1* mRNA in H9c2 cells treated with 500 μM H_2_O_2_, while this interaction was significantly inhibited by circCacna1c overexpression (Fig. 6A). No significant interaction was observed between Hnrnpf and *RIPK3* mRNA (Fig. 6B). Then, Hnrnpf was silenced in H9c2 cells using si-Hnrnpf. As depicted in Fig. 6C–E (Fig. S6A), the knockdown of Hnrnpf led to a significant decrease in the interaction between Hnrnpf and *RIPK1* mRNA, consequently resulting in a reduction in the protein level of RIPK1. Notably, the expression of *RIPK3* mRNA remained unaffected following Hnrnpf knockdown. Consistent with these findings, the overexpression of Hnrnpf intensified the interaction between Hnrnpf and RIPK1 mRNA while simultaneously upregulating its protein level (Fig. 6F–H; Fig. S6B). Next, the effect of circCacna1c and Hnrnpf on the expression of RIPK1 was investigated in cardiomyocytes undergoing necroptosis. H9c2 cells were exposed to 500 μΜ H_2_O_2_ followed by qRT-PCR and western blotting assays. As shown in Fig. 6I, J (Fig. S6C), overexpression of circCacna1c significantly attenuated the levels of *RIPK1* mRNA and RIPK1 protein, whereas the overexpression of Hnrnpf restored their diminished levels induced by circCacna1c overexpression, indicating that circCacna1c might promote RIPK1 degradation via Hnrnpf during cardiomyocyte necroptosis. Consistent with this finding, knockdown of FTO resulted in a significant upregulation of *RIPK1* mRNA and protein levels, while the downregulation of Hnrnpf attenuated the increased levels induced by FTO knockdown (Fig. 6K, L; Fig. S6D). Taken together, these data demonstrate that circCacna1c exerts a suppressive effect on RIPK1 transcription in necroptosis cardiomyocytes through competitive interaction with Hnrnpf.

### circCacna1c protects cardiomyocytes from ischemia damage induced necroptosis

**Fig. 7:**
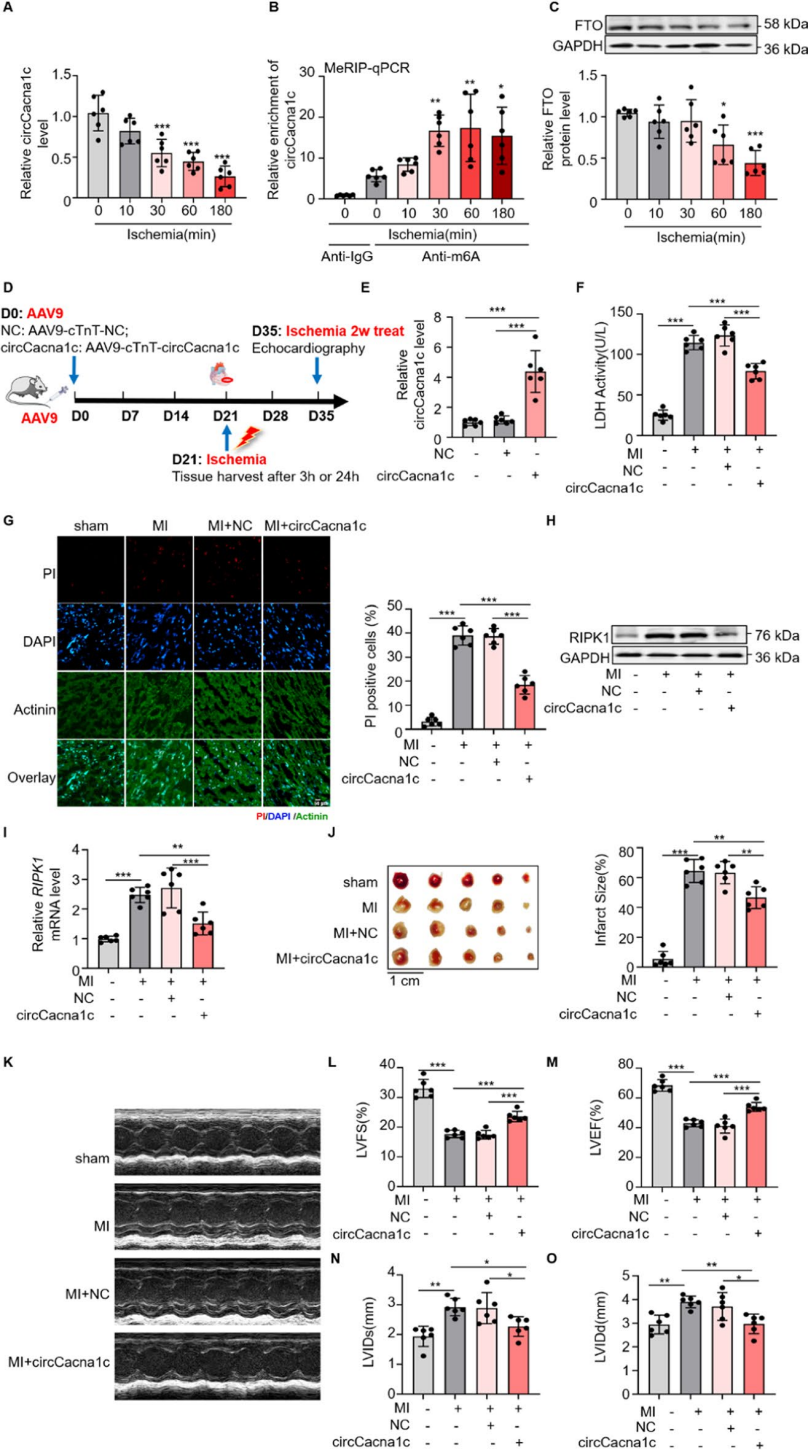
circCacna1c reduced cardiomyocytes necroptosis caused by MI injury and improved the long-term function of the heart after MI injury. **A** qRT-PCR was employed for the assessment of circCacna1c expression in mouse ischemic cardiac tissue. ***P < 0.001. *n* = 6. **B** The m^6^A modification level of circCacna1c in mouse ischemic cardiac tissue was analyzed using MeRIP–qPCR. *P < 0.05, **P < 0.01. *n* = 6. **C** The protein levels of FTO were determined in mouse ischemic cardiac tissue. GAPDH was selected as a reference. *P < 0.05, ***P < 0.001. *n* = 6. **D** Flow chart depicting the circCacna1c study conducted in a murine model of MI. **E** The cardiac tissue of mouse was subjected to AAV9-circCacna1c infection, and the quantification of circCacna1c levels in the hearts was performed using qRT-PCR. ***P < 0.001. *n* = 6. **F**, **G** The impact of circCacna1c on necroptosis in mouse ischemic cardiac tissue was assessed using experiments to detect the rate of PI positive cells and the activity of LDH. **F** The level of LDH in mouse ischemic cardiac tissue serum was measured. ***P < 0.001. *n* = 6. **G** A representative image is displayed above, while the calculated rates of necroptosis from six independent experiments are shown are shown below. Red indicates PI-positive nuclei, while blue represents DAPI stained nuclei. Green, cardiomyocytes labeled with antibody to Actinin. Scale bar, 50 μm. ***P < 0.001. *n* = 6. **H** The level of RIPK1 protein was analyzed by western blotting. *n* = 6. **I** The *RIPK1* mRNA level was analyzed by qRT-PCR. **P < 0.01, ***P < 0.001. *n* = 6. **J** Shown ratio of infarct (INF) area to total cardiac tissue area. **P < 0.01, ***P < 0.001. *n* = 6. **K**–**O** CircCacna1c rescues impaired heart function. **K** Echocardiography of the mouse hearts. **L** LVFS, fractional shortening of the left ventricular diameter. ***P < 0.001. *n* = 6. **M** LVEF, left ventricular ejection fraction. ***P < 0.001. *n* = 6. **N** LVIDs, systolic left ventricular internal diameters. *P < 0.05, **P < 0.01. *n* = 6. **O** LVIDd, left ventricular internal dimension d. *P < 0.05, **P < 0.01. *n* = 6

Necroptosis-mediated cardiomyocyte death is a major contributing factor to the pathogenesis of various CVDs, including MI [46, 47]. Given the significant protective role of circCacna1c against necroptosis in cardiomyocytes, we subsequently investigated its potential to provide protection against myocardial tissue injury using a mouse model of MI. The expression of circCacna1c and its m^6^A modification levels in ischemic myocardial tissues were assessed through qRT-PCR and MeRIP–qPCR. As depicted in Fig. 7A, B, the expression of circCacna1c showed a significant time-dependent decrease in the ischemic cardiac tissue of MI mouse, while its m^6^A modification level exhibited an inverse trend. Furthermore, the expression of FTO also demonstrated a concurrent reduction over time (Fig. 7C). The present results are in line with our previous in vitro findings. Moreover, the expression of circCacnna1 was not only reduced during the early stage of ischemia, but remained inhibition even after a duration of 3 weeks. This observation signifies the promising therapeutic potential of circCacna1c for CVDs (Fig. S7A). To investigate the relationship between circCacna1c depletion and cardiomyocyte injury, as well as its role in necroptosis, we conducted a cardiac-specific overexpression of circCacna1c by introducing AAV9 viral vectors encoding circCacna1c into mouse that underwent MI (Fig. 7D). The overexpression of circCacna1c led to a significant increase in its level in the hearts of mouse with MI compared to control hearts (Fig. 7E). Functional analysis revealed that the overexpression of circCacna1c significantly reduced the number of PI-positive cells in ventricular myocardium of MI mouse and decreased serum LDH activity, indicating a significant protection of myocardial necroptosis by circCacna1c (Fig. 7F, G). Simultaneously, we also discovered an intriguing phenomenon that overexpression of circCacna1c exerted no significant influence on the quantity of TUNEL-positive cells in the ventricular myocardium of MI mouse (Fig. S7B). Consistently, the myocardial tissues of circCacna1c overexpression mouse subjected to MI exhibited a significant reduction in both *RIPK1* mRNA and protein levels compared to control MI mouse (Fig. 7H, I; Fig. S7C). Furthermore, TTC staining revealed a reduction in the myocardial infarct size induced by MI injury in circCacna1c overexpressing hearts (Fig. 7J). Additionally, cardiac function exhibited improvement in MI-injured hearts overexpressing circCacna1c compared to control mouse hearts with MI injury (Fig. 7K–O). Collectively, these findings strongly support the notion that circCacna1c impedes cardiomyocyte necroptosis and mitigates cardiac dysfunction resulting from MI injury.

## Discussion

Cardiomyocyte necroptosis induced by I/R injury represents a fundamental pathological mechanism underlying MI [48, 49]. However, the detailed mechanisms involved in the regulation of necroptosis is still largely unknown, limiting our comprehensive understanding of MI pathogenesis. Therefore, it is imperative to gain further insights into the regulatory mechanisms governing cardiomyocyte necroptosis. In this study, we identified that circCacna1c is decreased in the ischemic cardiac tissue of mouse with MI and its depletion leads to necroptosis of cardiomyocytes. However, the overexpression of circCacna1c in MI injured heart or H_2_O_2_ exposed cardiomyocyte attenuates myocardial damage and improved cardiac function. Mechanistically, circCacna1c suppresses the nuclear translocation of Hnrnpf by direct binding to it, resulting in transcriptional inhibition of RIPK1, a key player involved in necroptosis. Furthermore, FTO-mediated demethylation of m^6^A-modified circCacna1c leads to YTHDF2-dependent degradation of circCacna1c during necroptosis in cardiomyocytes. Overall, our findings provide novel insights into the pathogenesis of MI and offer a new strategy for improving myocardial injury.

The dysregulation of circRNAs has been shown to contribute to cardiomyocyte death during MI [50]. However, the precise mechanism by which they are involved in cardiomyocyte necroptosis remains largely unclear. Zhao et al. discovered that the silencing of circCacna1c inhibits ISO-induced cardiac hypertrophy through the miR-29b-2-5p/NFATc1 axis [51]. This suggests that circCacna1c may play critical roles through different downstream target genes in various myocardial diseases. Further molecular biology research will be instrumental in elucidating the complex role of circCacna1c in myocardial disease. In this study, we demonstrate that circCacna1c is abundantly expressed in cardiomyocytes and its expression is decreased in H_2_O_2_ exposed cardiomyocytes and MI myocardial tissues. Additionally, the m^6^A modification is a prevalent epigenetic alteration observed in ncRNAs, playing a crucial role in regulating circRNA metabolism [23, 24, 52]. It has been reported that ALKBH5 mediates the demethylation of m^6^A-modified circCPSF6, thereby facilitating the degradation of circCPSF6 through a YTHDF2-dependent pathway [53]. Therefore, it is reasonable to hypothesize that m^6^A modification may contribute to the reduction of circCacna1c levels in MI-injured cardiomyocytes and myocardial tissue. Our data demonstrates that FTO plays a role in the demethylation process of m^6^A-modified circCacna1c in cardiomyocytes undergoing necroptosis. Furthermore, YTHDF2 functions as an m^6^A reader protein [24] to specifically recognize and bind to m^6^A-modified circCacna1c, thereby facilitating its subsequent degradation. These findings support the notion that m^6^A modification serves as a common mechanism for reducing the stability of circRNAs.

The current study demonstrates the role of circCacna1c and FTO in modulating cardiomyocyte necroptosis in myocardial tissues following MI. We observed that circCacna1c inhibits necroptosis in H_2_O_2_-exposed cardiomyocytes and MI myocardial tissues, while the FTO facilitates cardiomyocyte necroptosis by enhancing the stability of circCacna1c. The underlying mechanism of necroptosis is extremely complex and largely unknown. While our findings suggest that circCacna1c may be regulated through FTO-mediated degradation, it is important to note that other pathways may also directly or indirectly modulate circCacna1c. Additionally, further research is necessary to identify FTO-independent pathways involved in cardiomyocyte necroptosis and their roles in regulating circCacna1c.

RIPK1 and RIPK3, as members of the Ser/Thr/Tyr kinase family, play vital roles in determining the initiation of necroptosis in cardiomyocytes [44]. They trigger necroptosis by activating the MLKL cascade [11–13]. In this study, we observed that circCacna1c overexpression in cardiomyocytes undergoing necroptosis has an inhibitory effect on RIPK1 expression, while it has no impact on the expression of RIPK3. These data suggest that circCacna1c plays a protective role against cardiomyocyte necroptosis by modulating RIPK1 expression. Hnrnpf is a heterogeneous nuclear RNA protein that plays a crucial role in regulating RNA maturation. Its primary functions include splicing regulation, particularly selective splicing, as well as 5′-cap and 3′-polyadenylation of RNA, along with facilitating RNA export [54, 55]. MS and bioinformatic analysis have revealed a direct interaction between circCacna1c and Hnrnpf, establishing a strong correlation between circCacna1c-mediated downregulation of RIPK1 and Hnrnpf. Our findings demonstrate that circCacna1c directly binds to Hnrnpf, inhibiting its nuclear translocation and subsequently suppressing the expression of RIPK1 in necroptosis cardiomyocytes. This suggests an important role for circCacna1c in regulating the cellular processes associated with necroptosis in cardiomyocytes. However, there are still certain limitations in this study. For example, despite the observation of a direct interaction between circCacna1c and *RIPK1* mRNA, as well as an upregulation of both its mRNA and protein levels, the mechanism by which Hnrnpf regulates the splicing of *RIPK1* mRNA remains unknown. Further investigation is necessary to address this issue.

## Conclusion

This study presents the first evidence that circCacna1c functions as a negative regulator of cardiomyocyte necroptosis by inhibiting the nuclear translocation of Hnrnpf and subsequently suppressing the expression of RIPK1, a critical mediator of necroptosis, during the progression of myocardial infarction. The myocardial MI injury results in decreased levels of circCacna1c and increased nuclear translocation of Hnrnpf, thereby promoting the activation of necroptotic pathways in cardiomyocytes through upregulation of RIPK1 during MI progression. Furthermore, we reveal that the FTO is involved in regulating cardiomyocyte necroptosis by mediating demethylation of m^6^A modification on circCacna1c and facilitating its degradation. Our study not only identified circCacna1c as a master regulator of cardiomyocyte necroptosis but also provides a novel mechanism that connects the dysregulated m^6^A modification of circRNAs (e.g., circCacna1c) and upregulation of necroptosis associated factors (e.g., RIPK1) in MI-injured cardiomyocyte. Furthermore, the overexpression of circCacna1c leads to a significant improvement in cardiac function, accompanied by a notable reduction in cardiomyocyte necroptosis. This implies that targeting the FTO/circCacna1c/Hnrnpf/RIPK1 pathway could be a promising approach for mitigating necroptosis-induced loss of cardiomyocytes in ischemic cardiovascular diseases, particularly MI.

## Supplementary Information

Supplementary Material 1.

Supplementary Material 2.

## Data Availability

All data generated or analyzed during this study are included in this published article and its additional files. Further details were available from the corresponding author upon request.

## Abbreviations

circRNAs: Circular RNAs
MI: Myocardial infarction
CVDs: Cardiovascular diseases
RIPK1: Receptor-interacting protein kinase 1
RIPK1: Receptor-interacting protein kinase 3
FTO: Rat mass and obesity-associated protein
Hnrnpf: Heterogeneous nuclear ribonucleoprotein F
H_2_O_2_: Hydrogen peroxide
m^6^A: *N*^6^-Methyladenosine
ROS: Reactive oxygen species
AAV: Adeno-associated virus
LDH: Lactate dehydrogenase
RIP: RNA immunoprecipitation
MeRIP: Methylated RNA immunoprecipitation
METTL3: Methyltransferase-like protein 3
ALKBH5: Alkylated DNA repair protein alkB homolog 5
METTL14: Methyltransferase-like protein 14
WTAP: WT1-associated protein
YTHDF2: YTH Domain Family Protein 2
