# Supplementary material for: m6A-modified circCacna1c regulates necroptosis and ischemic myocardial injury by inhibiting Hnrnpf entry into the nucleus
Source: Cell Mol Biol Lett. 2024 Nov 12;29:140. doi: 10.1186/s11658-024-00649-8 (PMC11558890; doi:10.1186/s11658-024-00649-8)
Supplement: Supplementary file 1 — Supplementary Material 1. [file 11658_2024_649_MOESM1_ESM.docx]

**Supplementary Materials**

**Supplementary table 1 (Table S1). List of primers used in this study.**

| **Experiment** | **Name** | **Position or orientation** | **Sequence (5’-3’)** |
| --- | --- | --- | --- |
| PCR | circCacna1c (divergent, rat) | F | GTATGGGAAACCCAAGAAGCAG |
|  |  | R | AGTAGGGATGTGCTCGGGG |
|  | circCacna1c (convergent, rat) | F | CCCCGAGCACATCCCTACT |
|  |  | R | CTGCTTCTTGGGTTTCCCATAC |
| qPCR | GAPDH (rat) | F | ATGCCGCCTGGAGAAACC |
|  |  | R | GCATCAAAGGTGGAAGAATGG |
|  | U6 (rat) | F | CTCGCTTCGGCAGCACA |
|  |  | R | AACGCTTCACGAATTTGCGT |
|  | circCacna1c (rat) | F | ATCAGCATTGTTGAATGGAAATCA |
|  |  | R | GGCGTGGGCTCCCATAGT |
|  | circELF2 (rat) | F | GGTGCTGGTGTATGACGATGAG |
|  |  | R | CTAAGCATCCTTCACCGTTTCC |
|  | circSENP6 (rat) | F | GGCCTCCAGGATGAATGCTT |
|  |  | R | CTGCACGTGAAGGTTTTTGC |
|  | circNCX1 (rat) | F | CCTGTGGAGAGCTGGAATTCC |
|  |  | R | CCTCCTGTTTCTGCCTCTGTATC |
|  | circFndc3b (rat) | F | GCTATGGGAAGGGCCACAGT |
|  |  | R | TCCGGTGCTGTCTTCAATCA |
|  | Cacna1c (rat) | F | ACTGGATGCAAGACGCTATGG |
|  |  | R | CTCCTCGAGCTTTGGCTTTCT |
|  | RIPK1 (Rat) | F | ACGTGCTGAAGACCAAGGAAA |
|  |  | R | TGTGAAAGTCACGGTCAACGA |
|  | RIPK3 (Rat) | F | GACAGGCCATCCTTCCAAGA |
|  |  | R | GCTGGACTCTCTGGCAGACAA |
|  | GAPDH (mouse) | F | GCCACCCAGAAGACTGTGGAT |
|  |  | R | GGAAGGCCATGCCAGTGA |
|  | circCacna1c (mouse) | F | TGCATAAGCATTGTTGAATGGAA |
|  |  | R | GTGGGCTCCCATAGTTGGAA |
|  | RIPK1 (mouse) | F | GACTGTGTACCCTTACCTCCGA |
|  |  | R | CACTGCGATCATTCTCGTCCTG |

**Table S2. List of siRNAs for transient transfection.**

| **Name** | **siRNA** | **antisense** |
| --- | --- | --- |
| circCacna1c | si-circCacna1c | 5'- CCAUAGUUGGAACCAGGUUTT -3' |
| FTO | si-FTO | 5'- AUAUAAUCCAAGGUGCCUGTT -3' |
| YTHDF2 | si-YTHDF2 | 5'- AUAAGAAGUUAGAUAGGGCTT -3' |
| Hnrnpf | si-Hnrnpf | 5'- ACUGUAUUCUUCAUAGCCCTT -3' |
| Negative control siRNA | | 5'- ACGUGACACGUUCGGAGAATT -3' |

**Table S3.** **List of antibodies used in this study.**

| **Antibody** | **Vendor** | **Catalog No.** | **Application** |
| --- | --- | --- | --- |
| RIPK1 | Proteintech | 17519-1-AP | WB: 1:1000 |
| RIPK3 | Proteintech | 17563-1-AP | WB: 1:1000 |
| N6-methyladenosine | abcam | ab208577 | MeRIP:5 μg |
| Hnrnpf | Proteintech | 14974-1-AP | WB: 1:1000  RIP:5 μg |
| METTL3 | Proteintech | 15073-1-AP | WB: 1:1000 |
| METTL14 | Proteintech | 26158-1-AP | WB: 1:1000 |
| WTAP | Proteintech | 10200-1-AP | WB: 1:1000 |
| FTO | Proteintech | 27226-1-AP | WB: 1:1000 |
| ALKBH5 | Proteintech | 16837-1-AP | WB: 1:1000 |
| YTHDF2 | Proteintech | 24744-1-AP | WB: 1:1000  RIP:5 μg |
| GAPDH | Proteintech | 60004-1-Ig | WB: 1:30000 |
| Lamin B | Proteintech | 12987-1-AP | WB: 1:5000 |
| β-Tubulin | Proteintech | 10094-1-AP | WB: 1:2000 |
| Alpha Actinin | Proteintech | 11313-2-AP | IF: 1:50 |
| CoraLite488-conjugated Goat Anti-Rabbit IgG(H+L) | Proteintech | SA00013-2 | IF: 1:100 |
| HRP Goat Anti-Rabbit IgG (H+L) | abclonal | AS014 | RIP:5 μg |
| HRP Goat Anti-Mouse IgG (H+L) | abclonal | AS003 | RIP:5 μg |

**Table S4. Mass spectra obtained for circCacna1 pulldown proteins.**

| **Gene name** | **Accession** | **Description** | **Peptides** | **Score** | **Abundances** |
| --- | --- | --- | --- | --- | --- |
| Actg2 | P63269 | Actin, gamma-enteric smooth muscle | 25 | 5585 | 3463799 |
| Rplp0 | P19945 | 60S acidic ribosomal protein P0 | 7 | 222 | 23202996 |
| Hnrnpc | A0A0G2JXW4 | Heterogeneous nuclear ribonucleoprotein C, isoform CRA_a | 6 | 190 | 20052751 |
| Immt | Q3KR86 | MICOS complex subunit Mic60 (Fragment) | 4 | 150 | 4901241 |
| Hnrnpf | Q794E4 | Heterogeneous nuclear ribonucleoprotein F | 4 | 142 | 11812424 |
| Col1a1 | P02454 | Collagen alpha-1(I) chain | 5 | 126 | 12346916 |
| RGD1562451 | D3ZSR2 | Polyadenylate-binding protein | 2 | 118 | 1553643 |
| Rpl10a | P62907 | 60S ribosomal protein L10a | 3 | 117 | 10126092 |
| Nol5a | Q5RJN5 | Nol5a protein (Fragment) | 2 | 99 | 4293813 |
| Rbmx | Q4V898 | RNA-binding motif protein, X chromosome | 2 | 93 | 5779540 |
| Msi2 | F1LWE6 | Musashi RNA-binding protein 2 | 3 | 82 | 32987318 |
| - | P62890 | 60S ribosomal protein L30 | 2 | 80 | 7965997 |
| Fbl | P22509 | rRNA 2'-O-methyltransferase fibrillarin | 1 | 77 | 2815432 |
| Rpl3 | P21531 | 60S ribosomal protein L3 | 1 | 65 | 3446036 |
| Kif1c | F1M9C8 | Kinesin-like protein KIF1C | 1 | 64 | 2850821 |
| Sptbn1 | G3V6S0 | Spectrin beta chain | 4 | 64 | 3197688 |
| Slc25a11 | G3V6H5 | Mitochondrial 2-oxoglutarate/malate carrier protein | 1 | 56 | 1034193 |
| Matr3 | A0A0G2JSR7 | Matrin-3 | 3 | 55 | 5569412 |
| Rpl4 | Q6P3V9 | 60S ribosomal protein L4 | 2 | 54 | 3220087 |
| Pes1 | Q3B8N8 | Pescadillo homolog | 2 | 48 | 2371820 |
| Snu13 | P55770 | NHP2-like protein 1 | 1 | 47 | 2219791 |
| Aldoart2 | Q6AY07 | Fructose-bisphosphate aldolase | 2 | 46 | 3229127 |
| Pdia3 | A0A0H2UHM5 | Protein disulfide-isomerase | 1 | 42 | 2744418 |
| Arglu1 | Q5BJT0 | Arginine and glutamate-rich protein 1 | 1 | 41 | 2035711 |
| Rpl12 | P23358 | 60S ribosomal protein L12 | 2 | 41 | 11283085 |
| Nop58 | Q5PPK6 | Nol5 protein | 1 | 40 | 2347814 |
| Ncl | Q5U328 | Nucleolin | 2 | 40 | 2528853 |
| Eif4a3 | Q3B8Q2 | Eukaryotic initiation factor 4A-III | 1 | 39 | 4389153 |
| Sf3b2 | D3ZMS1 | Splicing factor 3b, subunit 2 | 1 | 38 | 1670120 |
| Tmem43 | Q5XIP9 | Transmembrane protein 43 | 1 | 38 | 3227486 |
| Krt222 | D4AC62 | Keratin 222 | 1 | 29 | 2672887 |
| Snrpd1 | B2RZB7 | Small nuclear ribonucleoprotein Sm D1 | 1 | 29 | 2251752 |
| Snrnp200 | F1LNJ2 | U5 small nuclear ribonucleoprotein 200 kDa helicase | 1 | 28 | 4870896 |
| Prpf8 | G3V6H2 | Pre-mRNA processing factor 8, isoform CRA_a | 1 | 28 | 218948.6 |
| Nom1 | M0R6J5 | Nucleolar protein with MIF4G domain 1 | 1 | 27 | 1695557 |
| Utp15 | A2RRU3 | U3 small nucleolar RNA-associated protein 15 homolog | 1 | 25 | 2488186 |
| Cavin4 | B1PRL5 | Caveolae-associated protein 4 | 1 | 25 | 2421650 |
| - | M0RCH8 | Ribosomal L1 domain-containing protein 1-like | 1 | 21 | 2872608 |
| Rnf20 | D3ZYQ9 | E3 ubiquitin protein ligase | 1 | 0 | 1972192 |
| Unc13b | A0A0G2K511 | Protein unc-13 homolog B | 1 | 0 | 1427324 |

**Supplementary** **figure and Supplementary** **figure legends**

**Supplementary figure 1 (Fig. S1)**


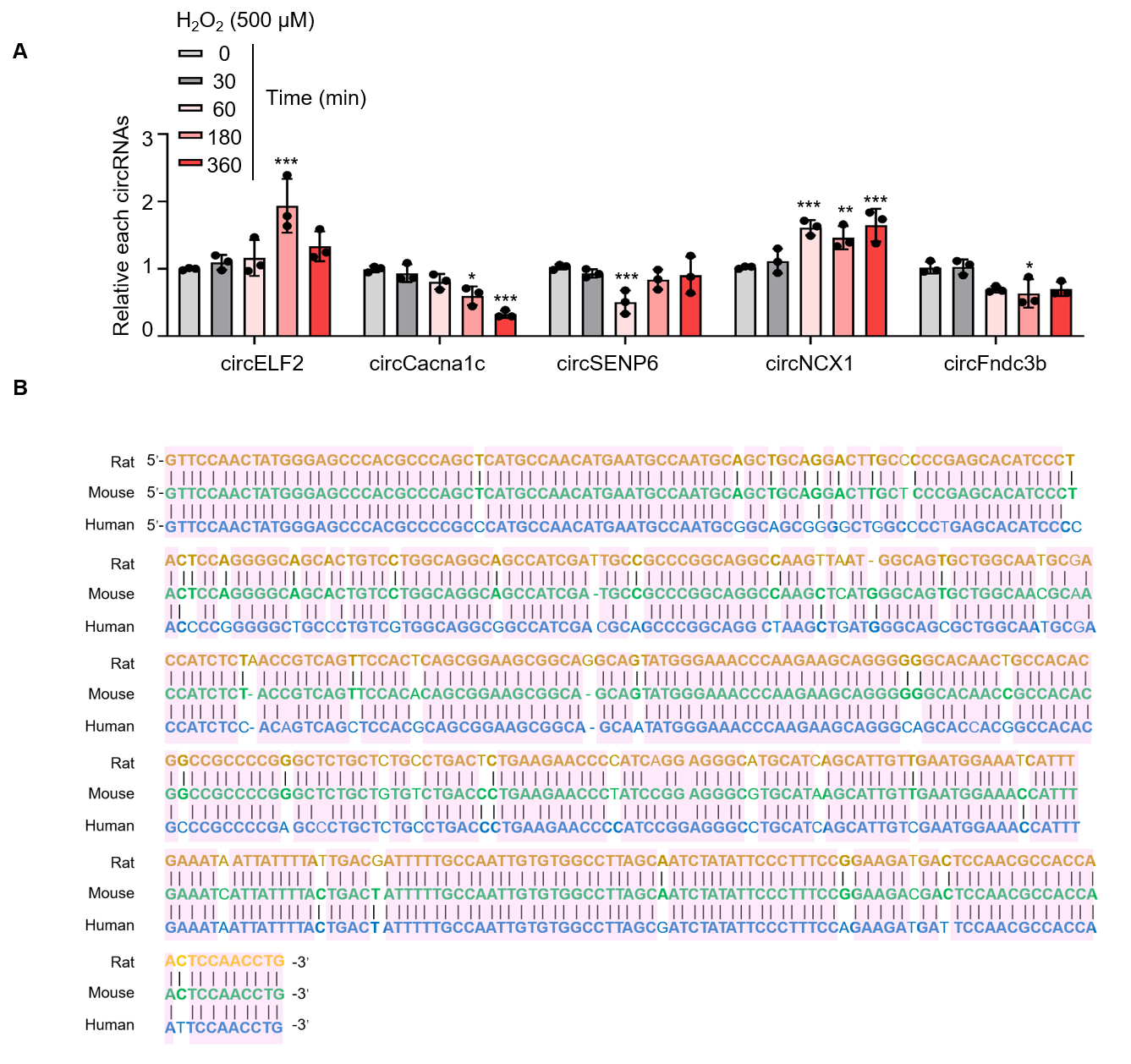


**Fig. S1. Verification of circCacna1c (Supplementary).** (A) H9c2 cells were treated with 500 μΜ H_2_O_2_ for 6 h. The expression levels of five circRNAs was analyzed by qRT-PCR. GAPDH was selected as reference. ^*^*P* < 0.05, ^**^*P* < 0.01, ^***^*P* < 0.001. n = 3. (B) Blast of circCacna1c sequence in rat, mouse and human.

**Fig. S2**


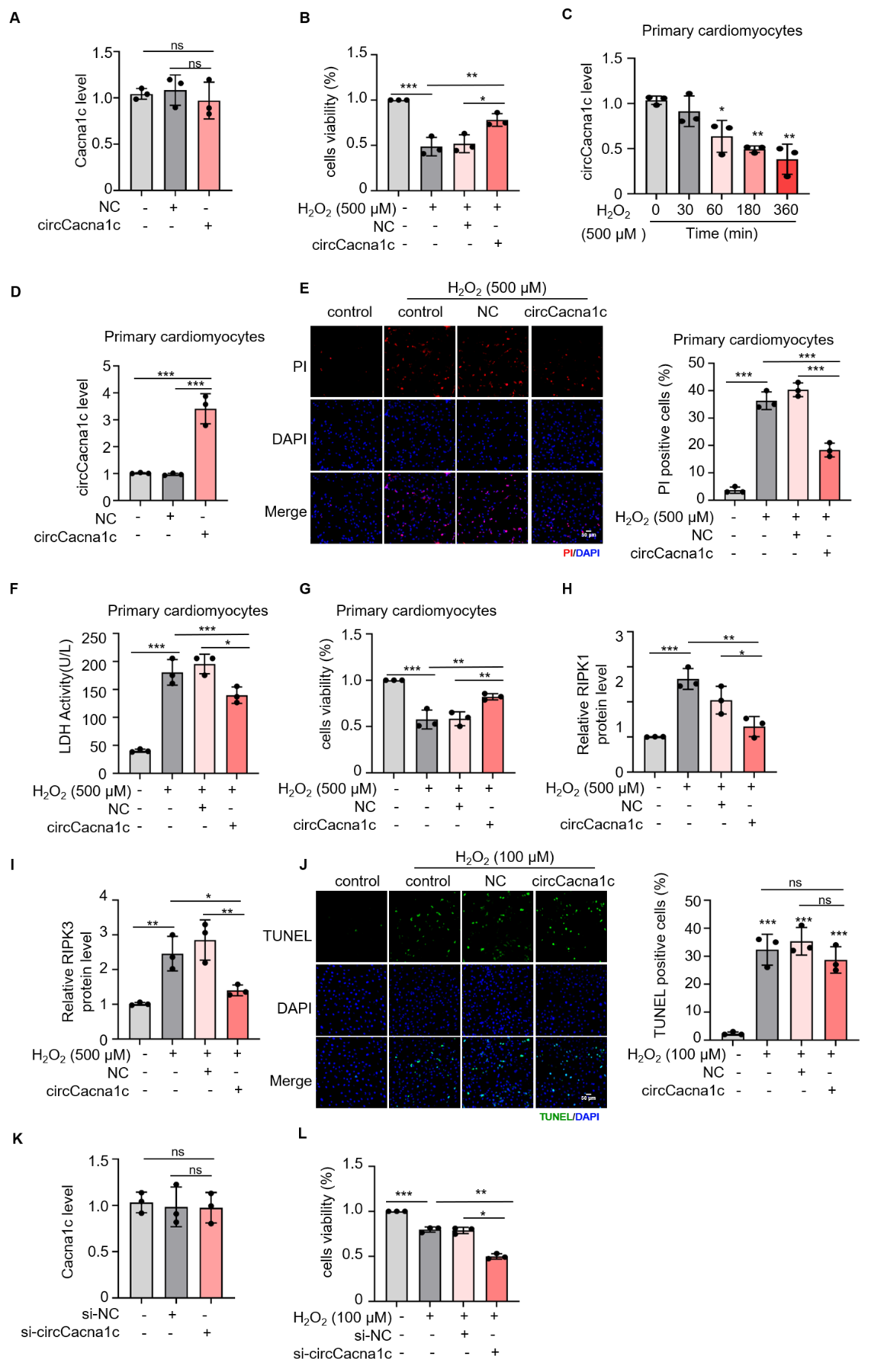


**Fig. S2. circCacna1c inhibits H_2_O_2_-induced necroptosis (Supplementary).** (A) H9c2 cells were transfected with the circCacna1c expression vector. The expression level of Cacna1c was analyzed by qRT-PCR. ns > 0.05. n = 3. (B) The impact of circCacna1c on H9c2 cell necroptosis was assessed through the CCK-8 cell activity assay. ^*^*P* < 0.05, ^**^*P* < 0.01, ^***^*P* < 0.001. n = 3. (C) Primary cardiomyocytes were treated with H_2_O_2_ (500 μΜ), and the expression level of circCacna1c was determined using qRT-PCR with GAPDH mRNA as the internal reference gene for normalization. ^*^*P* < 0.05, ^**^*P* < 0.01 versus 0 h. n = 3. (D) Primary cardiomyocytes were transfected with the circCacna1c expression vector, and the expression level of circCacna1c was analyzed by qRT-PCR. The empty vector was used as a NC (empty vector). ^***^*P* < 0.001. n = 3. (E-G) The impact of circCacna1c on necroptosis in primary cardiomyocytes was assessed through experiments detecting the rate of PI-positive cells, the activity of LDH, and the cells viability. (E) A representative image is displayed on the left side, while the calculated rates of necroptosis from three independent experiments are shown on the right side. Red indicates PI-positive nuclei, while blue represents DAPI stained nuclei. Scale bars, 50 μm.  ^***^*P* < 0.001. n = 3. (F) The level of LDH in the cell supernatant was measured. ^*^*P* < 0.05, ^***^*P* < 0.001. n = 3. (G) CCK-8 measures cell activity. ^**^*P* < 0.01, ^***^*P* < 0.001. n = 3. (H-I) The protein levels of RIPK1 and RIPK3 were quantified in H9c2 cells, with GAPDH selected as a reference. n = 3. ^*^*P* < 0.05, ^**^*P* < 0.01, ^***^*P* < 0.001. n = 3. (J) The impact of circCacna1c on the apoptosis of H9c2 cells was assessed by determining the rate of TUNEL-positive cells. Green indicates TUNEL-positive nuclei, while blue represents DAPI stained nuclei. Scale bars, 50 μm.  ^***^*P* < 0.001, ns > 0.05. n = 3. (K) The si- circCacna1c was transfected into H9c2 cells, and the expression level of Cacna1c was assessed using qRT-PCR. ns > 0.05. n = 3. (L) CCK-8 measures cell activity. ^*^*P* < 0.05, ^**^*P* < 0.01, ^***^*P* < 0.001. n = 3.

**Fig. S3**


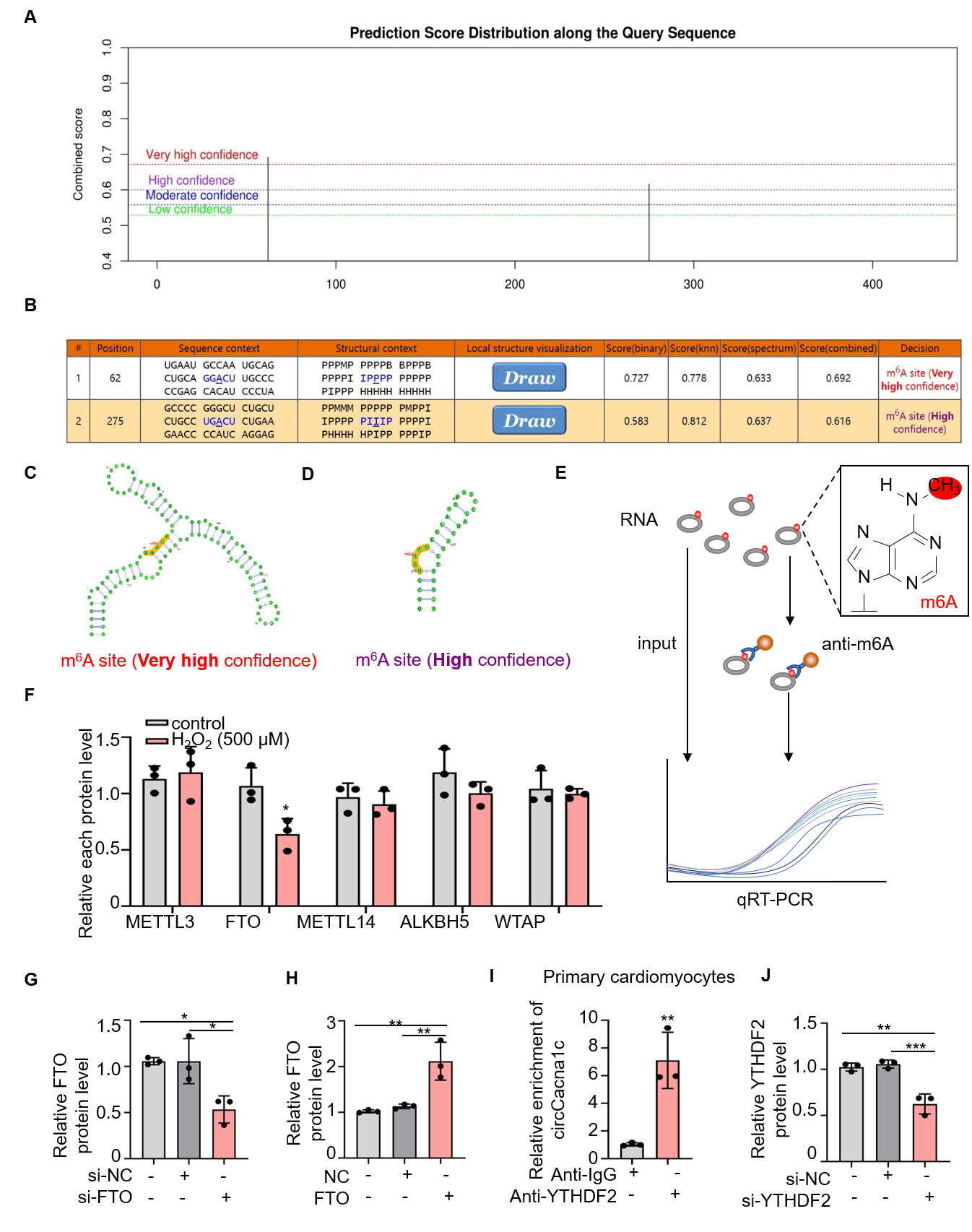


**Fig. S3. circCacna1c is regulated by FTO-mediated m6A methylation (Supplementary).** (A-D) Specific primers directed at the predicted m6A sites were designed for MeRIP detection. (E) Schematic diagram of MeRIP-qPCR detection scheme. (F) After exposing H9c2 cells to a concentration of 500 μM H_2_O_2_ for a duration of 6 hours, the cells were collected and subsequently analyzed for protein levels of METTL3, FTO, METTL14, ALKBH5 and WTAP. GAPDH was selected as a reference. ^*^*P* < 0.05. n = 3. (G) The FTO siRNA (si- FTO) was transfected into H9c2 cells, followed by the determination of FTO protein levels. GAPDH was selected as a reference. ^*^*P* < 0.05. n = 3. (H) The H9c2 cells were transfected with a plasmid that overexpressed FTO, followed by the determination of FTO protein levels. GAPDH was selected as a reference. ^**^*P* < 0.01. n = 3. (I) The YTHDF2 antibody was utilized in the RIP assay to determine its affinity for circCacna1c. The qRT-PCR was employed to assess the level of circCacna1c enrichment facilitated by the YTHDF2 antibody in primary cardiomyocytes. ^**^*P* < 0.01. n = 3. (J) The YTHDF2 siRNA (si- YTHDF2) was transfected into H9c2 cells, followed by the determination of YTHDF2 protein levels. ^**^*P* < 0.01, ^***^*P* < 0.001. n = 3.

**Fig. S4**


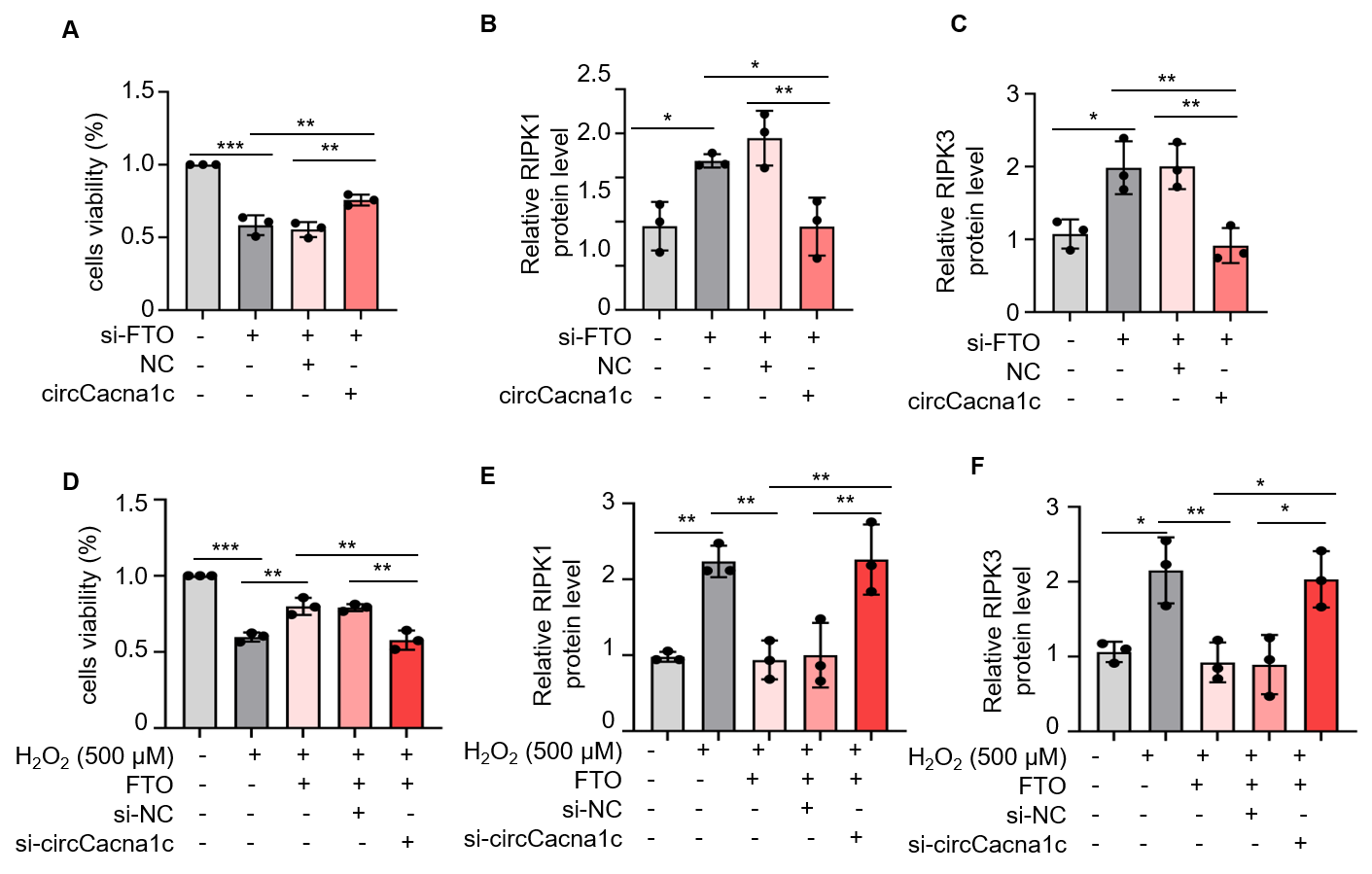


**Fig. S4. FTO is involved in the regulation of necroptosis by regulating the m6A modification of circCacna1c (Supplementary).** (A) CCK-8 measures cell activity. ^**^*P* < 0.01, ^***^*P* < 0.001. n = 3. (B-C) The protein levels of RIPK1 and RIPK3 were determined. GAPDH was selected as a reference. ^*^*P* < 0.05, ^**^*P* < 0.01. n = 3. (D) CCK-8 measures cell activity. ^**^*P* < 0.01, ^***^*P* < 0.001. n = 3. (E-F) The protein levels of RIPK1 and RIPK3 were determined. GAPDH was selected as a reference. ^*^*P* < 0.05, ^**^*P* < 0.01. n = 3. (D) CCK-8 measures cell activity. ^*^*P* < 0.05, ^**^*P* < 0.01. n = 3.

**Fig. S5**


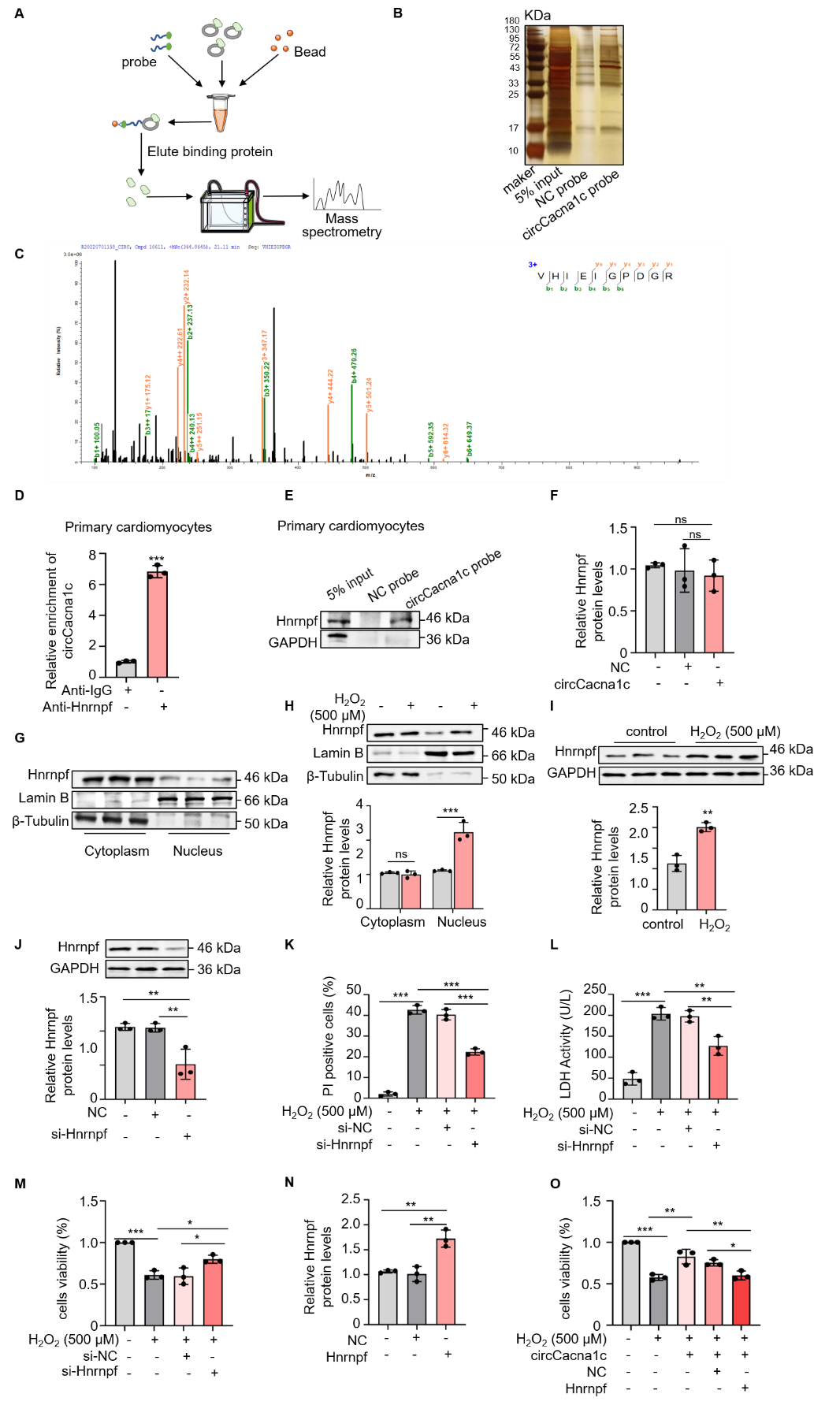


**Fig. S5. circCacna1c binds to Hnrnpf and inhibits nuclear translocation of Hnrnpf (Supplementary).** (A) Schematic diagram of RNA pull-down experiment and mass spectrometry analysis flow chart. (B) circCacna1c probe-captured proteins was analyzed by LC–MS/MS analysis. (C) LC-MS/MS analysis in circCacna1c probe immunopurified protein complex identified Hnrnpf. Fixed modification: Carbamidomethyl, Dynamical modifications: oxidation (M), acetyl (K) (protein N-term). (D) The Hnrnpf antibody was utilized in the RIP assay to determine its affinity for circCacna1c. The qRT-PCR was employed to assess the level of circCacna1c enrichment facilitated by the Hnrnpf antibody in primary cardiomyocytes. ^***^*P* < 0.001. n = 3. (E) RNA pulldown was used to detect the binding of Hnrnpf to circCacna1c in primary cardiomyocytes. n = 3. (F) H9c2 cells were transfected with the circCacna1c expression vector, followed by the determination of Hnrnpf protein levels. ns > 0.05. n = 3. (G) The cytoplasmic and nuclear components of H9c2 cells were isolated, and the Hnrnpf protein was determined. Lamin B and β-Tubulin were respectively utilized as the control for the nuclear and cytoplasmic compartments. n = 3. (H) H9c2 cells were exposed to 500 μM H_2_O_2_ for 6 h, the cytoplasmic and nuclear fractions were separated and the protein quantity of Hnrnpf was measured. Lamin B and β-Tubulin acted as controls for nuclear and cytoplasmic compartments respectively, with their relative protein levels being determined. ^***^*P* < 0.001. n = 3. (I) H9c2 cells were exposed to 500 μM H_2_O_2_ for 6 h, and the protein quantity of Hnrnpf was measured. GAPDH was selected as a reference. ^**^*P* < 0.01. n = 3. (J) The Hnrnpf siRNA (si- Hnrnpf) was transfected into H9c2 cells, followed by the determination of Hnrnpf protein levels. ^**^*P* < 0.01. n = 3. (K-M) The impact of Hnrnpf on necroptosis in H9c2 cells was assessed through experiments detecting the rate of PI-positive cells, the activity of LDH and cell activity. ^*^*P* < 0.05, ^**^*P* < 0.01, ^***^*P* < 0.001. n = 3. (N) The H9c2 cells were transfected with a plasmid that overexpressed Hnrnpf, followed by the determination of Hnrnpf protein levels. GAPDH was selected as a reference. ^**^*P* < 0.01. n = 3. (O) CCK-8 measures cell activity. ^*^*P* < 0.05, ^**^*P* < 0.01, ^***^*P* < 0.001. n = 3.

**Fig. S6**


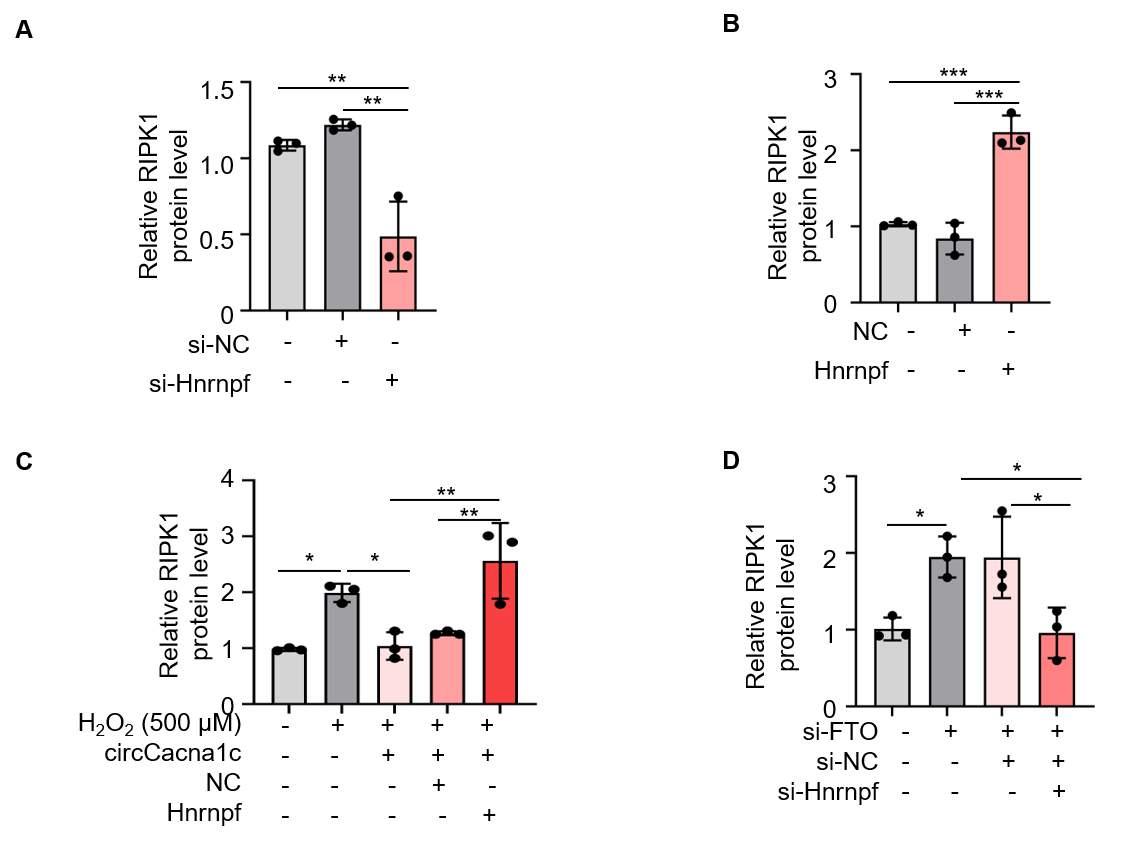


**Fig. S6. circCacna1c inhibits RIPK1 expression by interacting with Hnrnpf (Supplementary).** (A) The Hnrnpf siRNA (si-Hnrnpf) was transfected into H9c2 cells. The purpose of cell collection was to assess the protein levels of RIPK1. GAPDH was selected as a reference. ^**^*P* < 0.01. n = 3. (B) The H9c2 cells were transfected with a plasmid that overexpressed Hnrnpf. The purpose of cell collection was to assess the protein levels of RIPK1. GAPDH was selected as a reference. ^***^*P* <0.001. n = 3. (C) H9c2 cells were transfected with circCacna1c expression vectors and Hnrnpf expression vectors, then exposed to 500 μM H_2_O_2_ for a period of 6 h. The purpose of cell collection was to assess the protein levels of RIPK1. GAPDH was selected as a reference. ^*^*P* < 0.05, ^**^*P* < 0.01. n = 3. (D) H9c2 cells were transfected with si-FTO and si-Hnrnpf. The purpose of cell collection was to assess the protein levels of RIPK1. GAPDH was selected as a reference. ^*^*P* < 0.05. n = 3.

**Fig. S7**


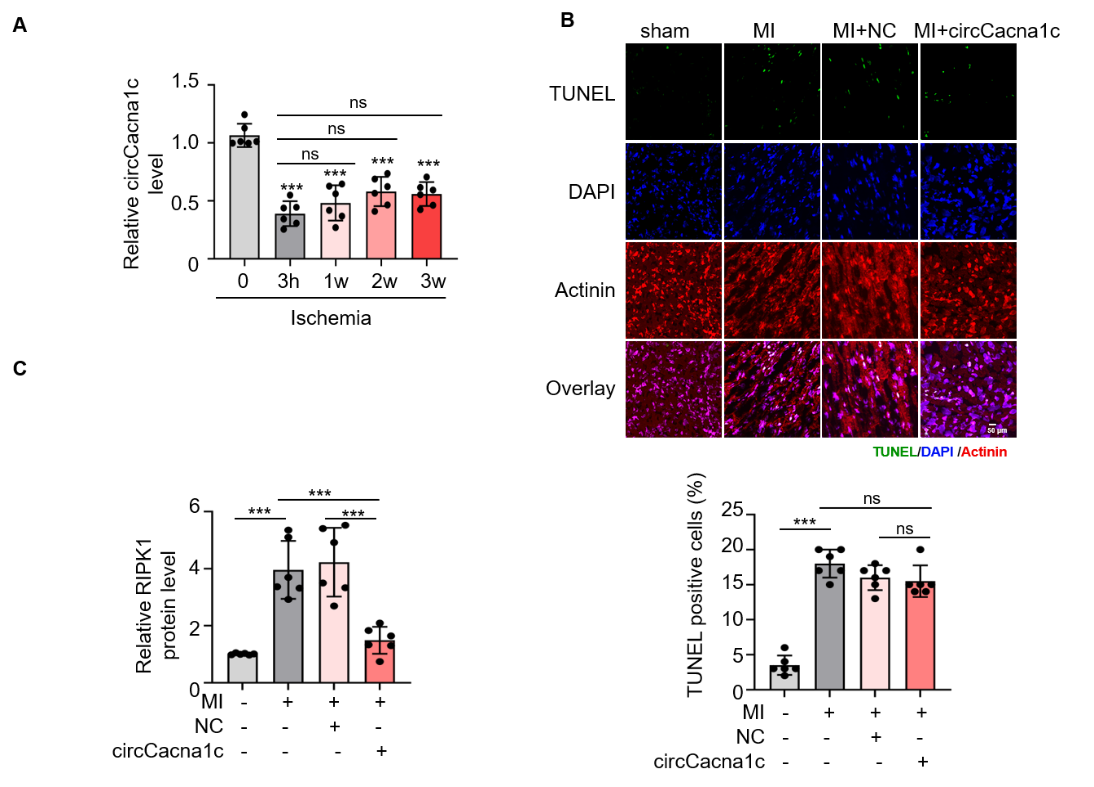


**Fig. S7. circCacna1c reduced cardiomyocytes necroptosis caused by MI injury and improved the long-term function of the heart after MI injury (Supplementary).** (A) qRT-PCR was employed for the assessment of circCacna1c expression in mouse ischemic cardiac tissue. ^***^*P* < 0.001, ns > 0.05. n=6. (B) The impact of circCacna1c on the apoptosis of ischemic heart tissue in mouse cardiac tissue was assessed by determining the rate of TUNEL-positive cells. A representative image is displayed above, while the calculated rates of apoptosis from six independent experiments are shown are shown below. Green indicates TUNEL positive nuclei, while blue represents DAPI stained nuclei. Red, cardiomyocytes labeled with antibody to Actinin. Scale bar, 50 μm. ^***^*P* < 0.001, ns > 0.05. n=6. (C) The level of RIPK1 protein was analyzed by western blotting. ^***^*P* < 0.001. n=6.
