# Supplementary material for: m6A-modified circCacna1c regulates necroptosis and ischemic myocardial injury by inhibiting Hnrnpf entry into the nucleus
Source: Cell Mol Biol Lett. 2024 Nov 12;29:140. doi: 10.1186/s11658-024-00649-8 (PMC11558890; doi:10.1186/s11658-024-00649-8)
Supplement: Supplementary file 2 — Supplementary Material 2. [file 11658_2024_649_MOESM2_ESM.pdf]

Supplementary file- original figures of gel and blots

Figure 1D:

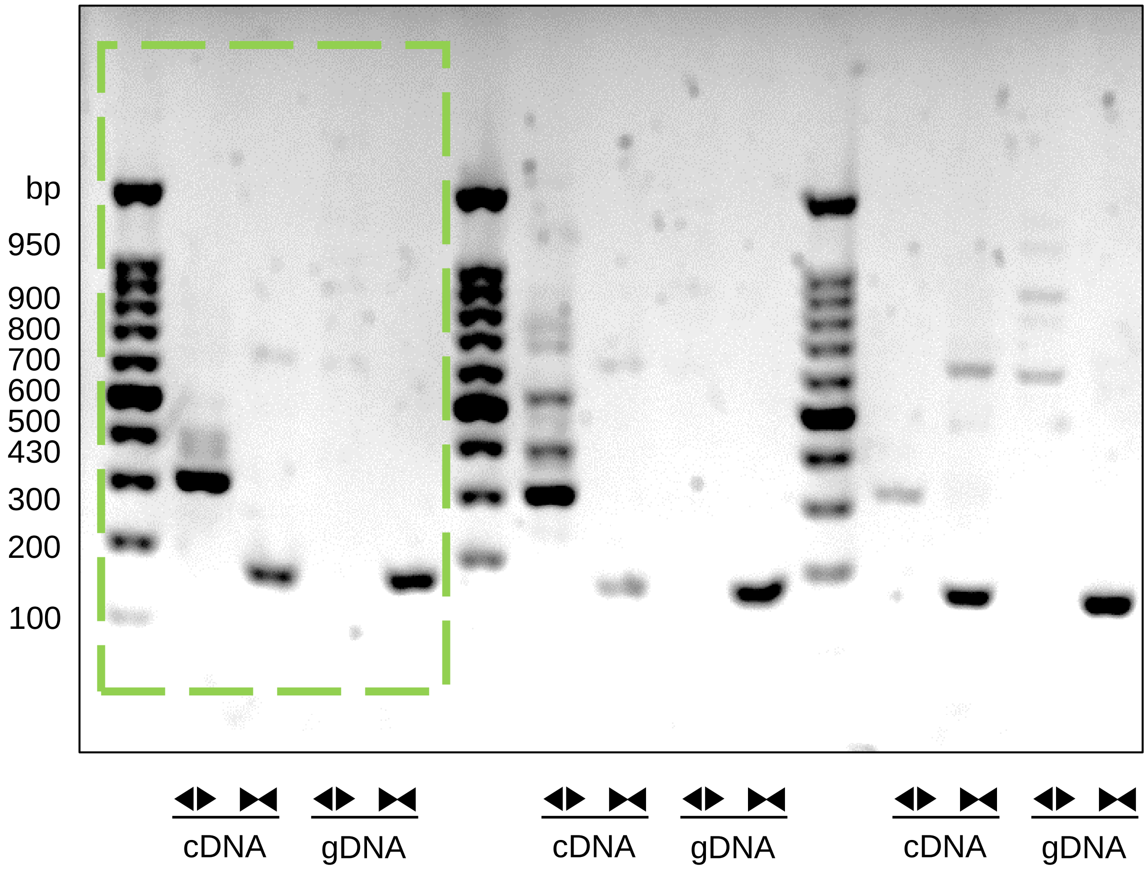

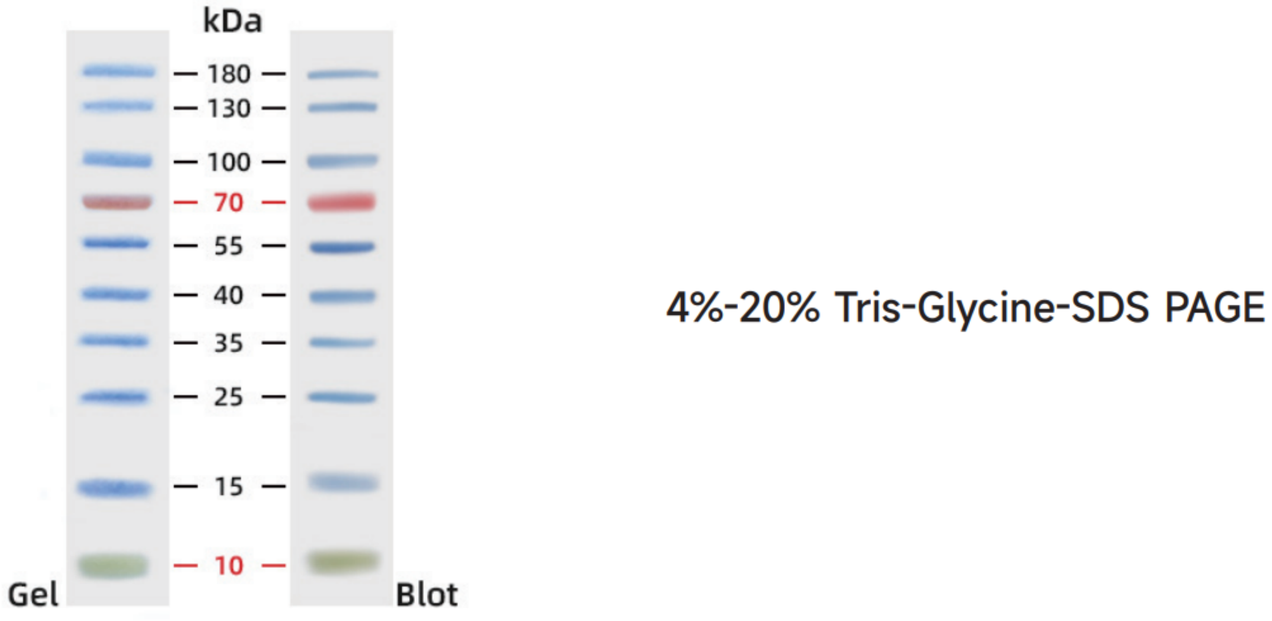

In all gel blotting experiments, the Tricolor Prestained Protein Ladder (Shanghai Epizyme Biomedical Technology Co., Ltd) was employed to indicate the size presented on the blot.

Figure 2D:

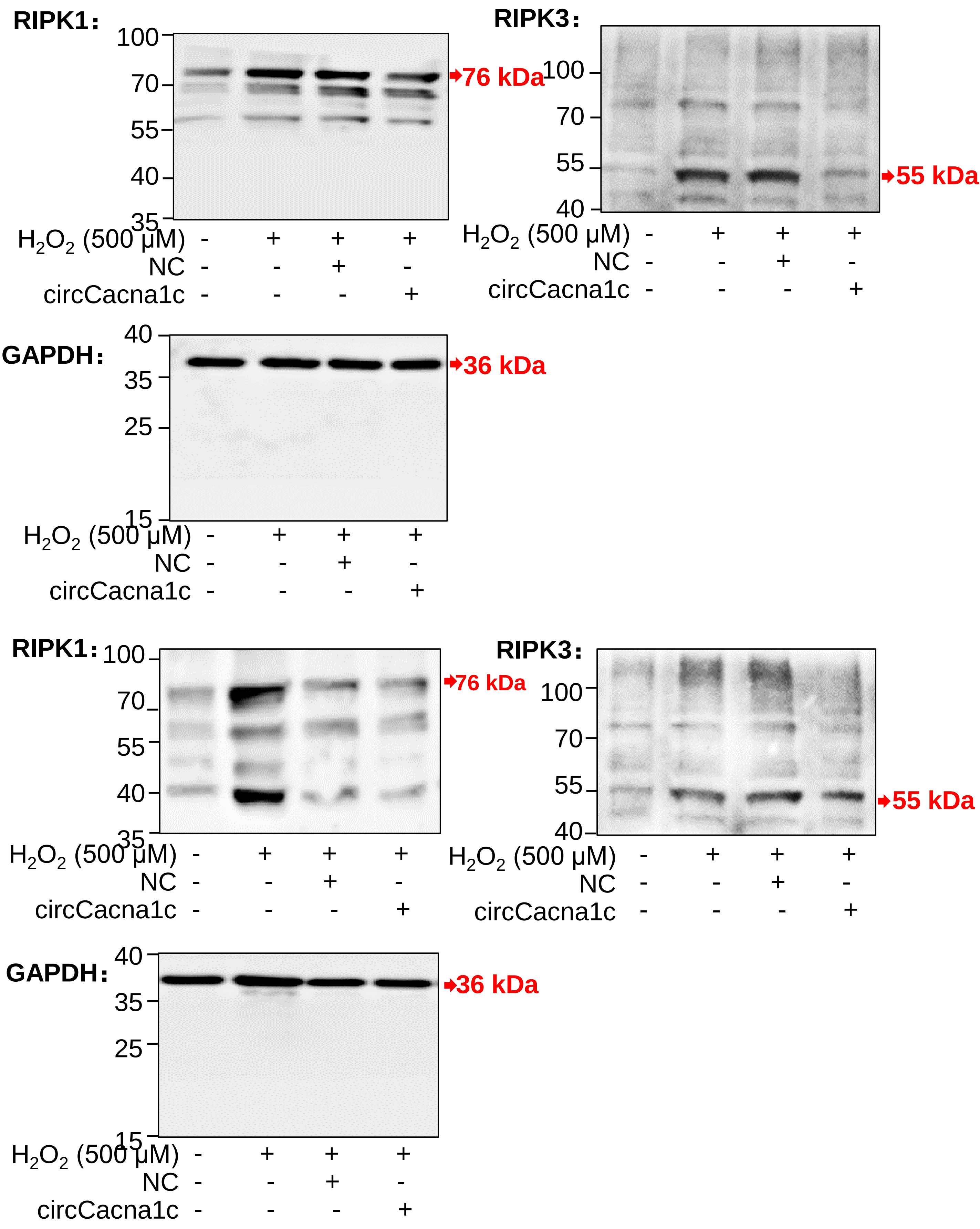
RIPK1:
100
70
55
40
35
76 kDa
H2O2 (500 μM) - + + +
NC - - + -
circCacna1c - - - +
RIPK3:
100
70
55
40
55 kDa
H2O2 (500 μM) - + + +
NC - - + -
circCacna1c - - - +
GAPDH:
40
35
25
15
36 kDa
H2O2 (500 μM) - + + +
NC - - + -
circCacna1c - - - +
RIPK1:
100
70
55
40
35
76 kDa
H2O2 (500 μM) - + + +
NC - - + -
circCacna1c - - - +
RIPK3:
100
70
55
40
55 kDa
H2O2 (500 μM) - + + +
NC - - + -
circCacna1c - - - +
GAPDH:
40
35
25
15
36 kDa
H2O2 (500 μM) - + + +
NC - - + -
circCacna1c - - - +

Figure 2D:

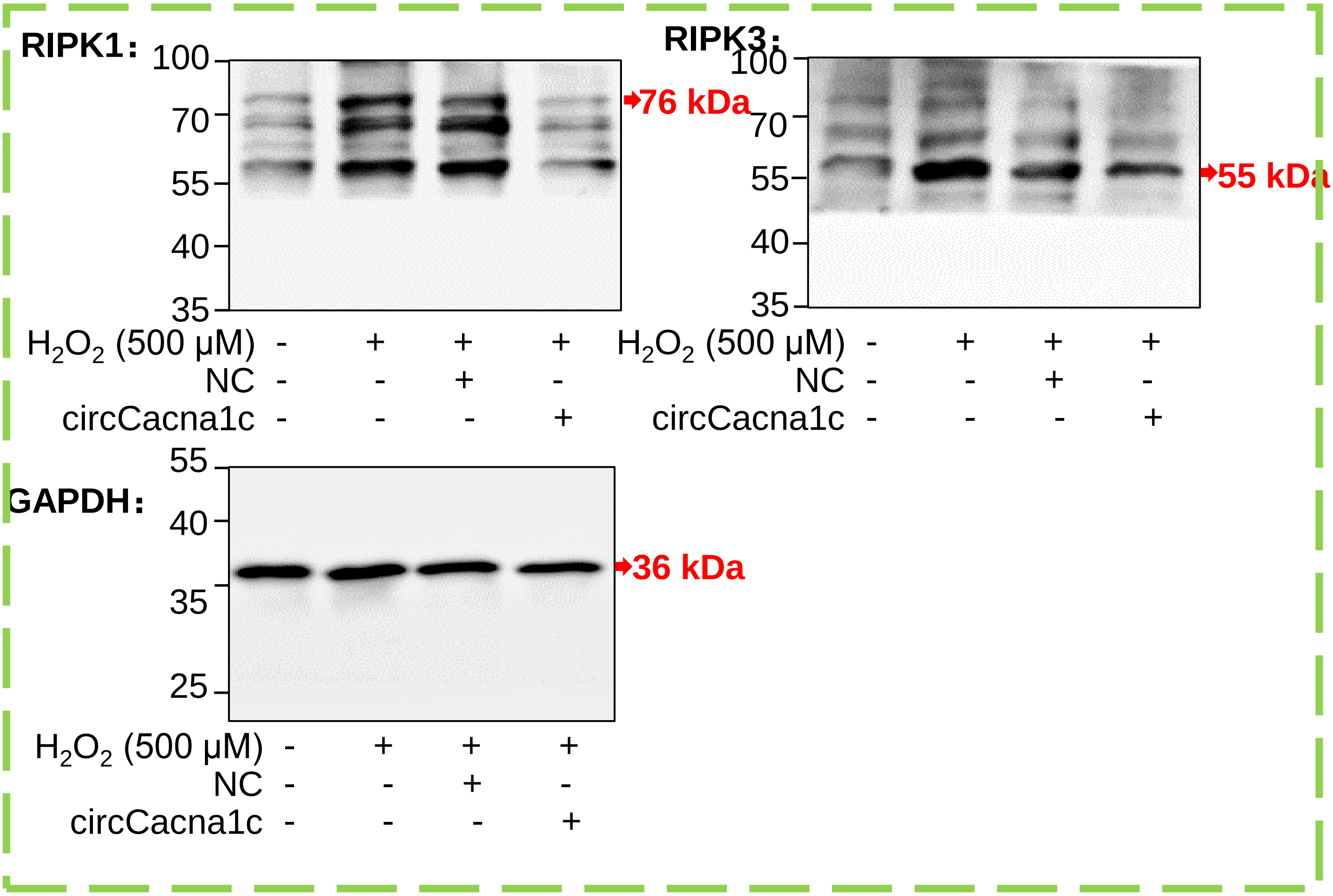

RIPK1:
100
70
55
40
35
76 kDa
H_2O_2 (500 μM) - + + +
NC - - + -
circCacna1c - - - +
RIPK3:
100
70
55
40
35
55 kDa
H_2O_2 (500 μM) - + + +
NC - - + -
circCacna1c - - - +
GAPDH:
55
40
35
25
36 kDa
H_2O_2 (500 μM) - + + +
NC - - + -
circCacna1c - - - +

Figure 3G:

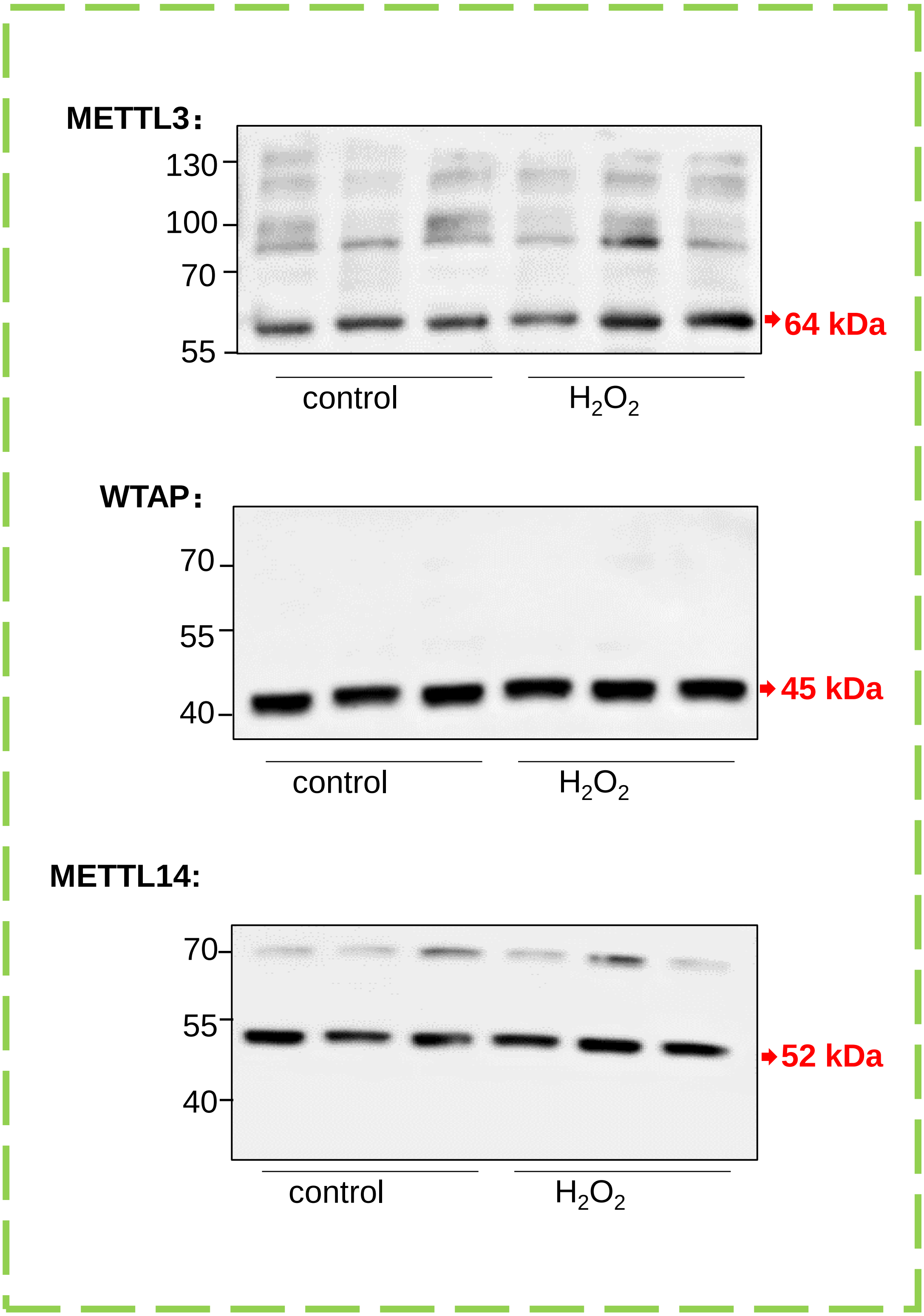
METTL3:
130
100
70
55
64 kDa
control
H2O2
WTAP:
70
55
40
45 kDa
control
H2O2
METTL14:
70
55
40
52 kDa
control
H2O2

Figure 3G:

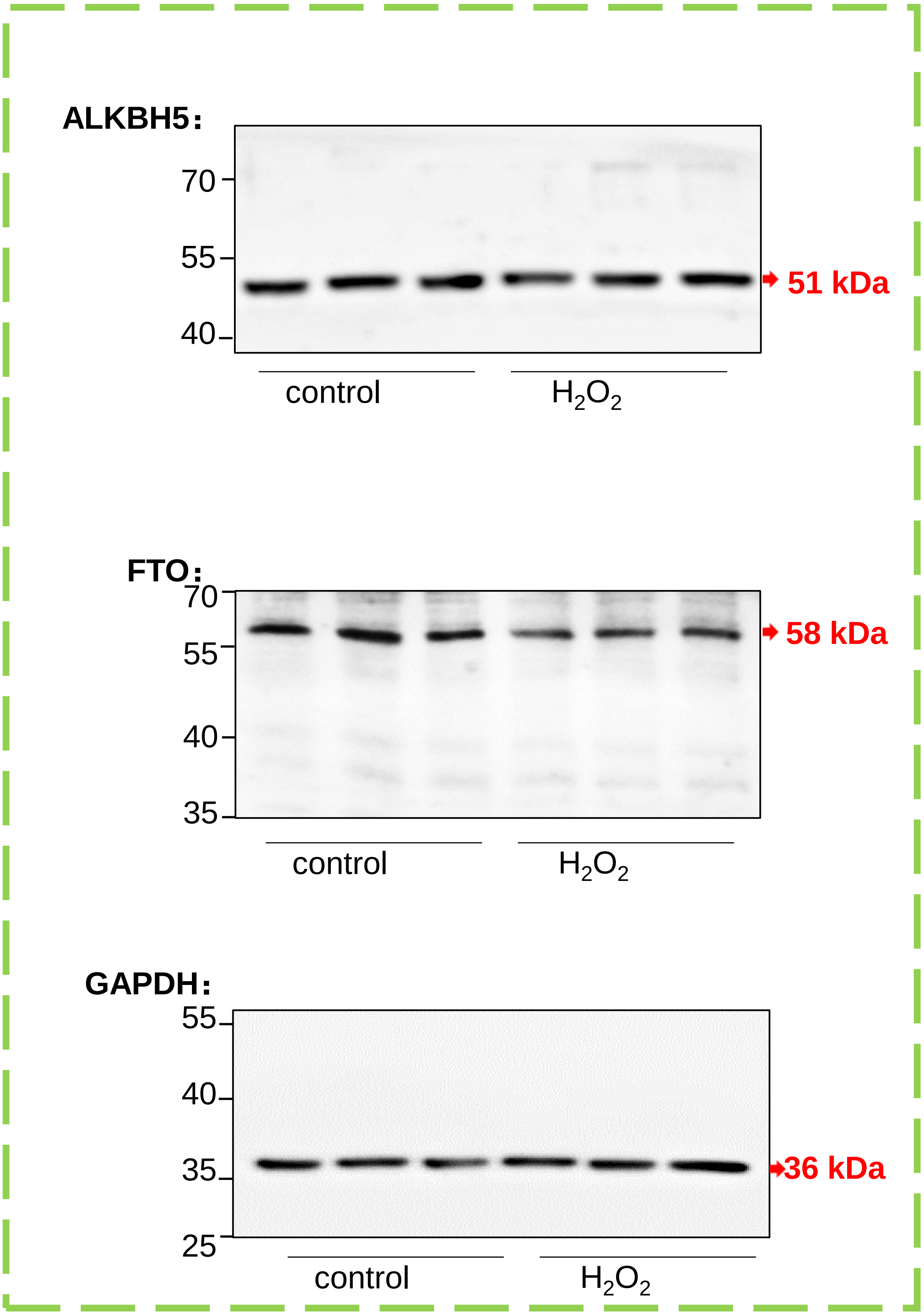
ALKBH5:
70
55
40
51 kDa
control
H2O2
FTO:
70
55
40
35
58 kDa
control
H2O2
GAPDH:
55
40
35
25
36 kDa
control
H2O2

Figure 3H:

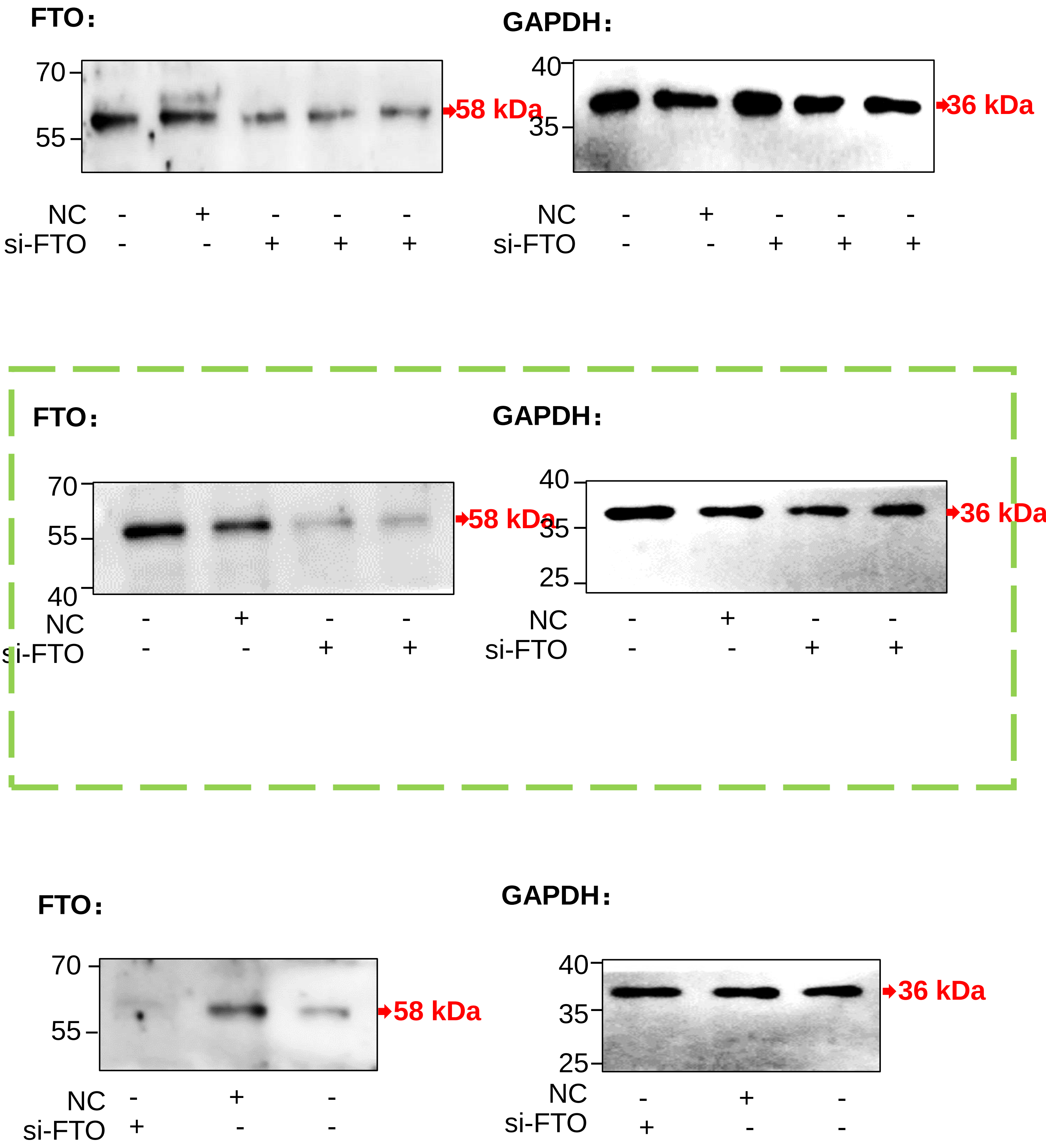
FTO:
70
55
58 kDa
NC - + - - -
si-FTO - - + + +
GAPDH:
40
35
36 kDa
NC - + - - -
si-FTO - - + + +
FTO:
70
55
40
58 kDa
NC - + - -
si-FTO - - + +
GAPDH:
40
35
25
36 kDa
NC - + - -
si-FTO - - + +
FTO:
70
55
58 kDa
NC - + -
si-FTO + - -
GAPDH:
40
35
25
36 kDa
NC - + -
si-FTO + - -

Figure 3M:

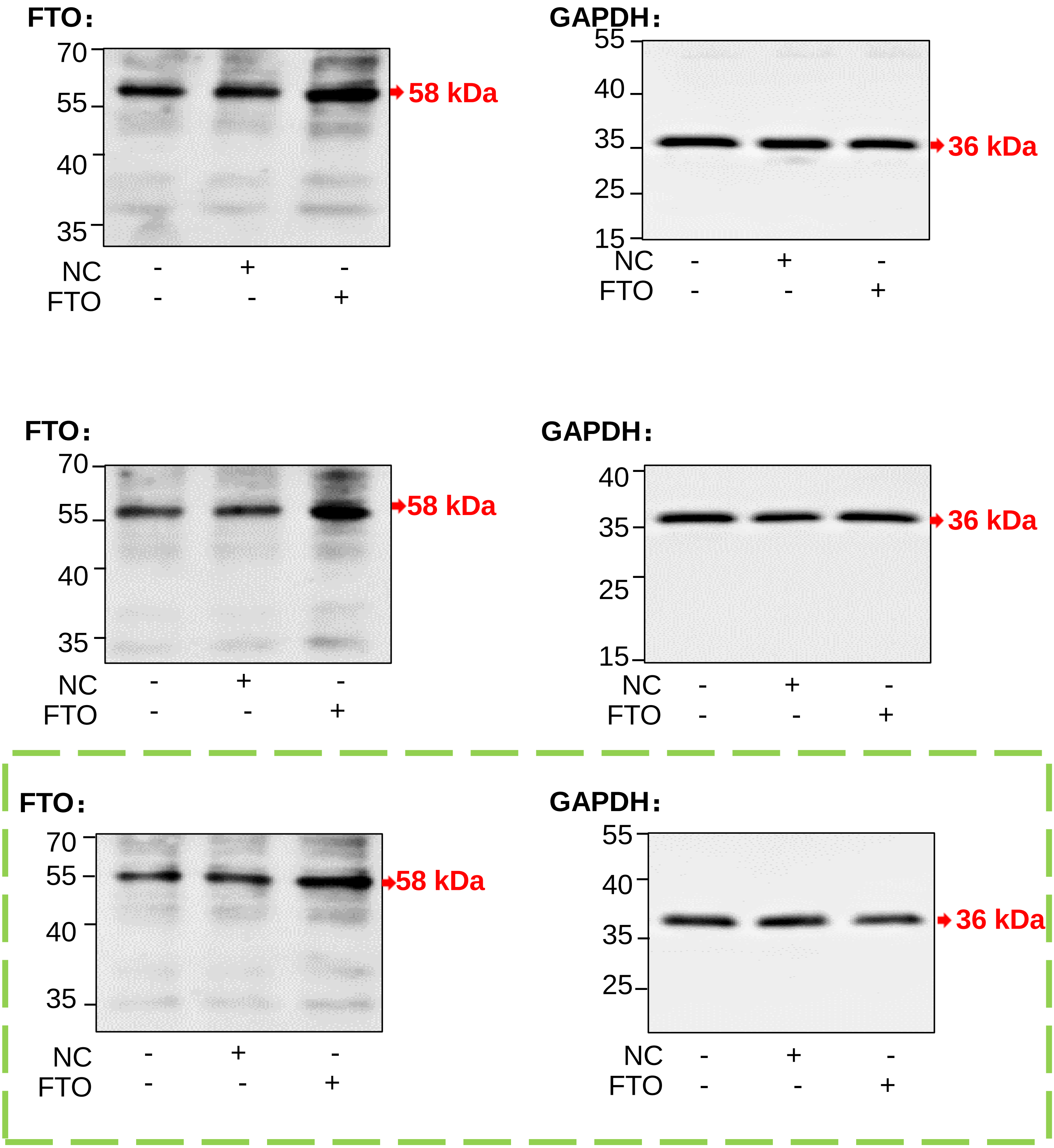
FTO:
70
55
40
35
58 kDa
NC - + -
FTO - - +
GAPDH:
55
40
35
25
15
36 kDa
NC - + -
FTO - - +
FTO:
70
55
40
35
58 kDa
NC - + -
FTO - - +
GAPDH:
40
35
25
15
36 kDa
NC - + -
FTO - - +
FTO:
70
55
40
35
58 kDa
NC - + -
FTO - - +
GAPDH:
55
40
35
25
36 kDa
NC - + -
FTO - - +

Figure 3P：

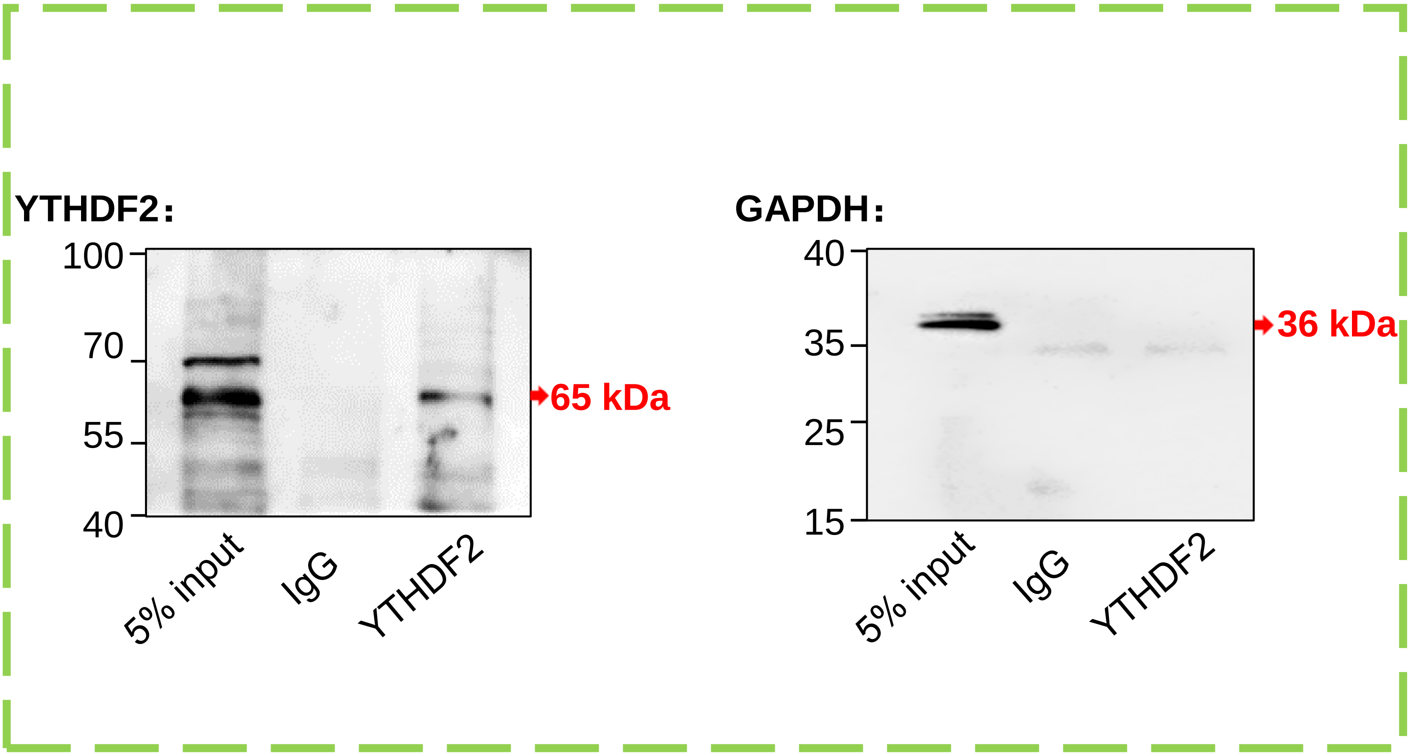

YTHDF2：
100
70
55
40
65 kDa
5% input
IgG
YTHDF2
GAPDH：
40
35
25
15
36 kDa
5% input
IgG
YTHDF2

Figure 3Q:

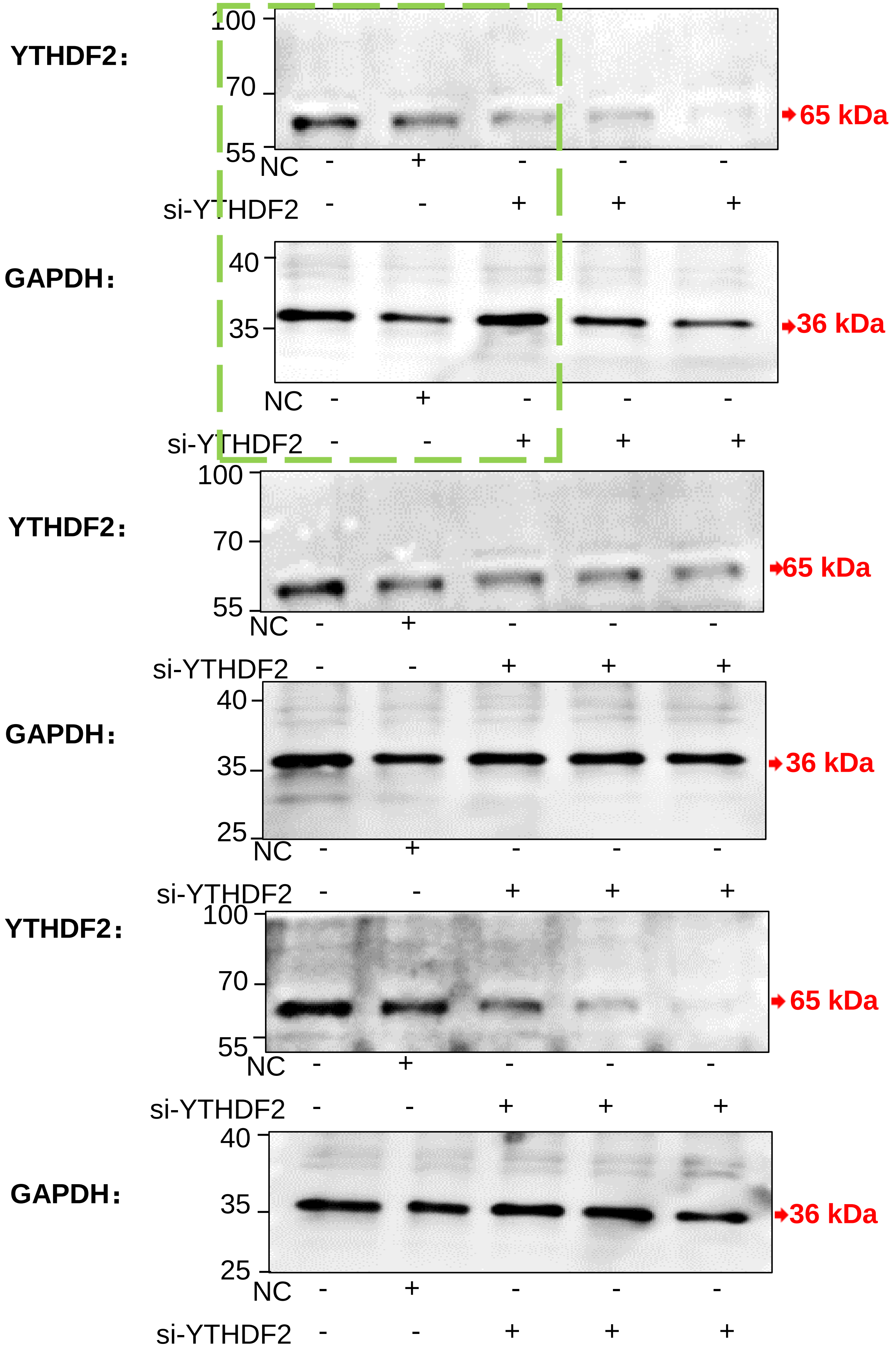
YTHDF2:
100
70
55
65 kDa
NC - + - - -
si-YTHDF2 - - + + +
GAPDH:
40
35
36 kDa
NC - + - - -
si-YTHDF2 - - + + +
YTHDF2:
100
70
55
65 kDa
NC - + - - -
si-YTHDF2 - - + + +
GAPDH:
40
35
25
36 kDa
NC - + - - -
si-YTHDF2 - - + + +
YTHDF2:
100
70
55
65 kDa
NC - + - - -
si-YTHDF2 - - + + +
GAPDH:
40
35
25
36 kDa
NC - + - - -
si-YTHDF2 - - + + +

Figure 4C：

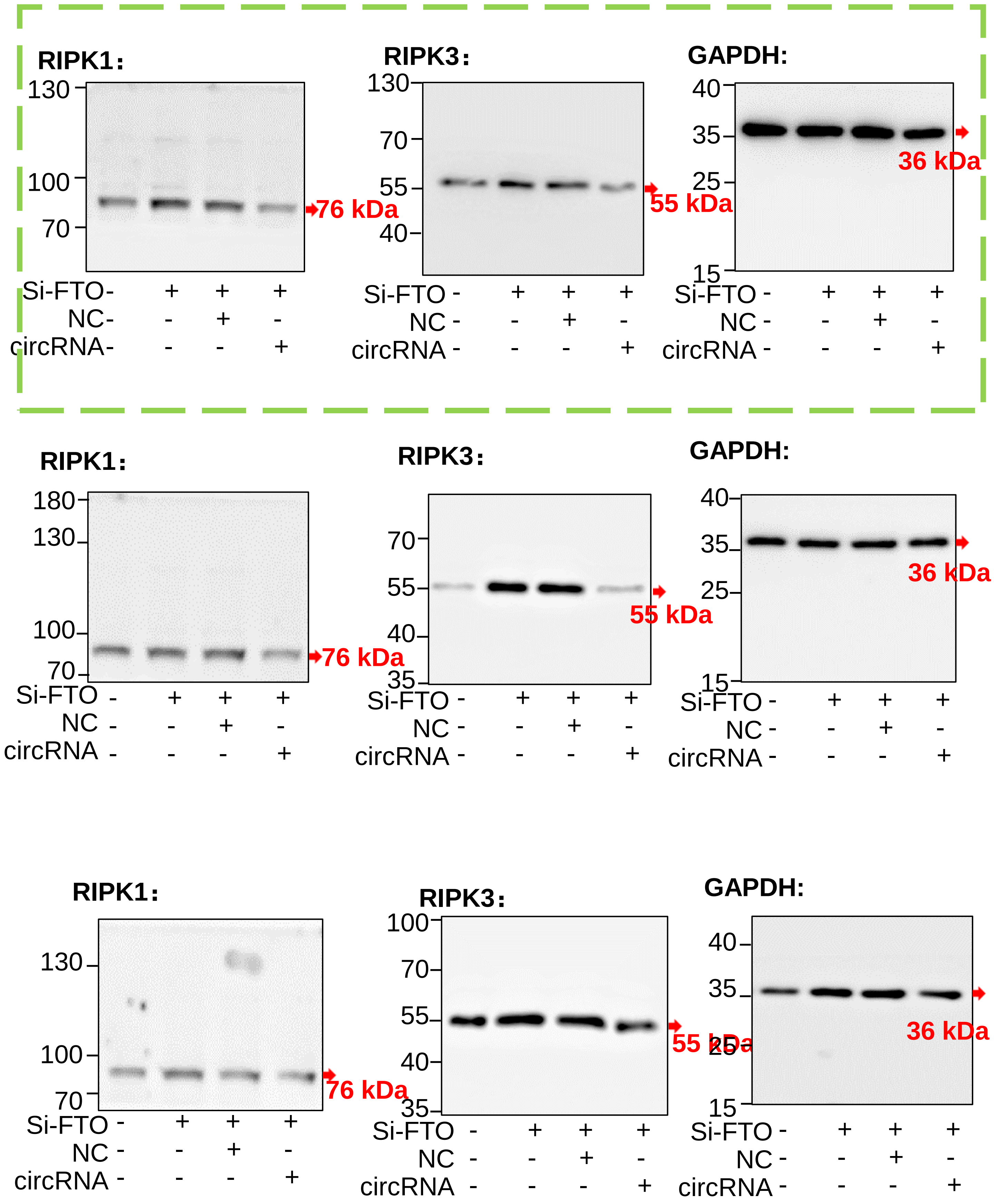

RIPK1：
130
100
70
76 kDa
Si-FTO - + + +
NC - - + -
circRNA - - - +
RIPK3：
130
70
55
40
55 kDa
Si-FTO - + + +
NC - - + -
circRNA - - - +
GAPDH:
40
35
25
15
36 kDa
Si-FTO - + + +
NC - - + -
circRNA - - - +
RIPK1：
180
130
100
70
76 kDa
Si-FTO - + + +
NC - - + -
circRNA - - - +
RIPK3：
70
55
40
35
55 kDa
Si-FTO - + + +
NC - - + -
circRNA - - - +
GAPDH:
40
35
25
15
36 kDa
Si-FTO - + + +
NC - - + -
circRNA - - - +
RIPK1：
130
100
70
76 kDa
Si-FTO - + + +
NC - - + -
circRNA - - - +
RIPK3：
100
70
55
40
35
55 kDa
Si-FTO - + + +
NC - - + -
circRNA - - - +
GAPDH:
40
35
25
15
36 kDa
Si-FTO - + + +
NC - - + -
circRNA - - - +

Figure 4F:

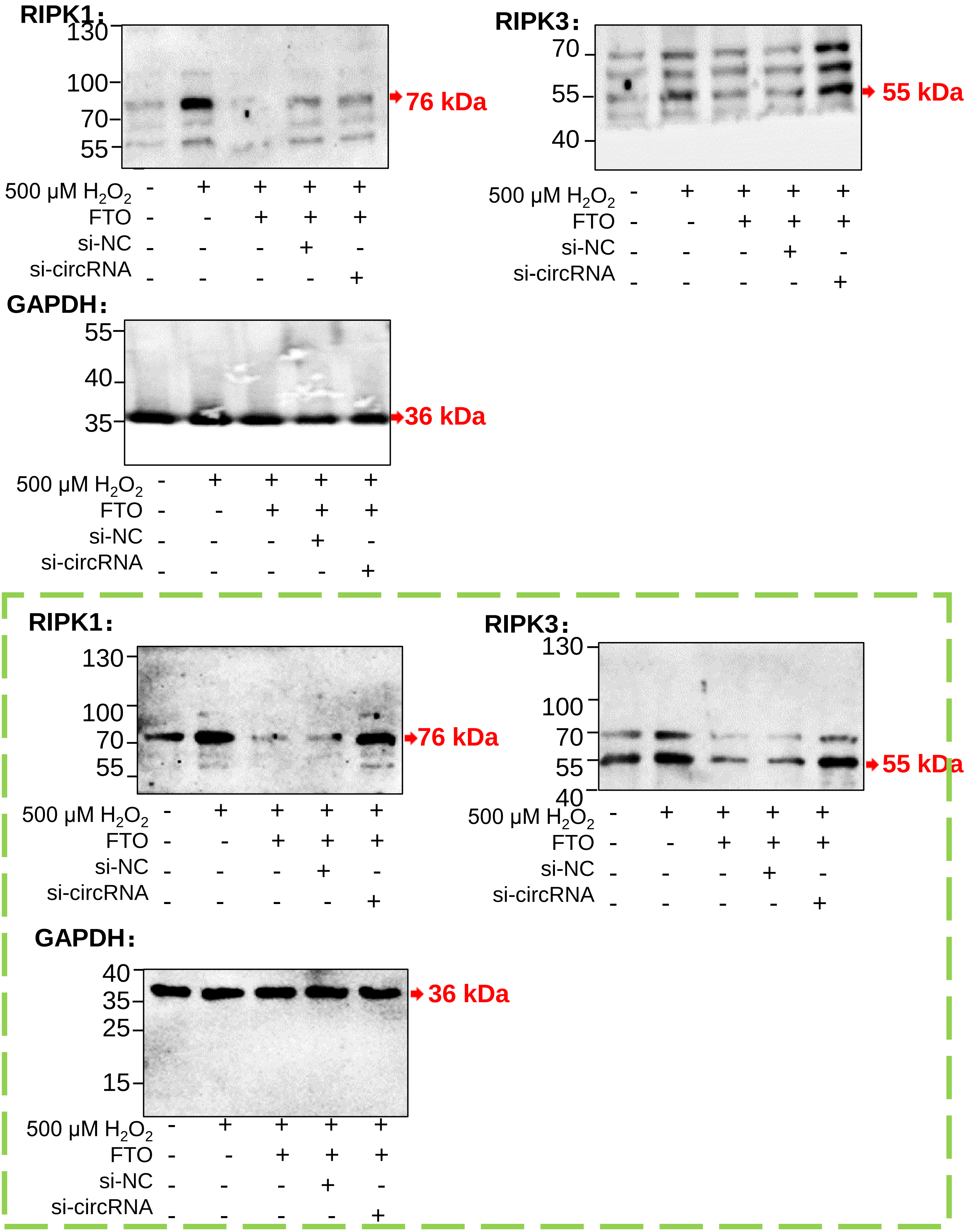

RIPK1:
130
100
70
55
76 kDa
RIPK3:
70
55
40
55 kDa
500 μM H2O2 - + + + +
FTO - - + + +
si-NC - - - + -
si-circRNA - - - - +
GAPDH:
55
40
35
36 kDa
RIPK1:
130
100
70
55
76 kDa
RIPK3:
130
100
70
55
40
55 kDa
GAPDH:
40
35
25
15
36 kDa

Figure 4F:

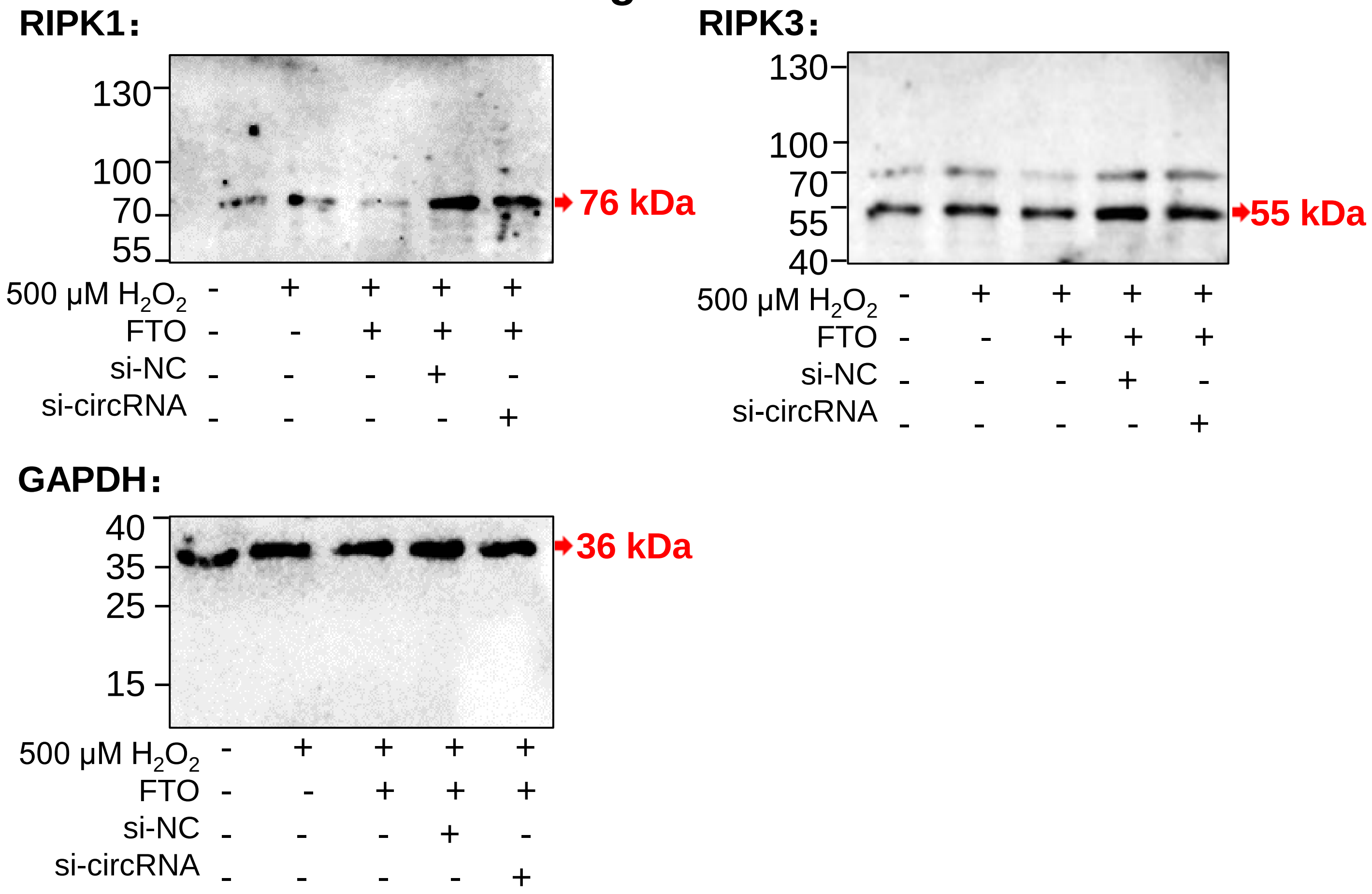
RIPK1:
130
100
70
55
76 kDa
500 μM H2O2 - + + + +
FTO - - + + +
si-NC - - - + -
si-circRNA - - - - +
RIPK3:
130
100
70
55
40
55 kDa
500 μM H2O2 - + + + +
FTO - - + + +
si-NC - - - + -
si-circRNA - - - - +
GAPDH:
40
35
25
15
36 kDa
500 μM H2O2 - + + + +
FTO - - + + +
si-NC - - - + -
si-circRNA - - - - +

Figure 5C：

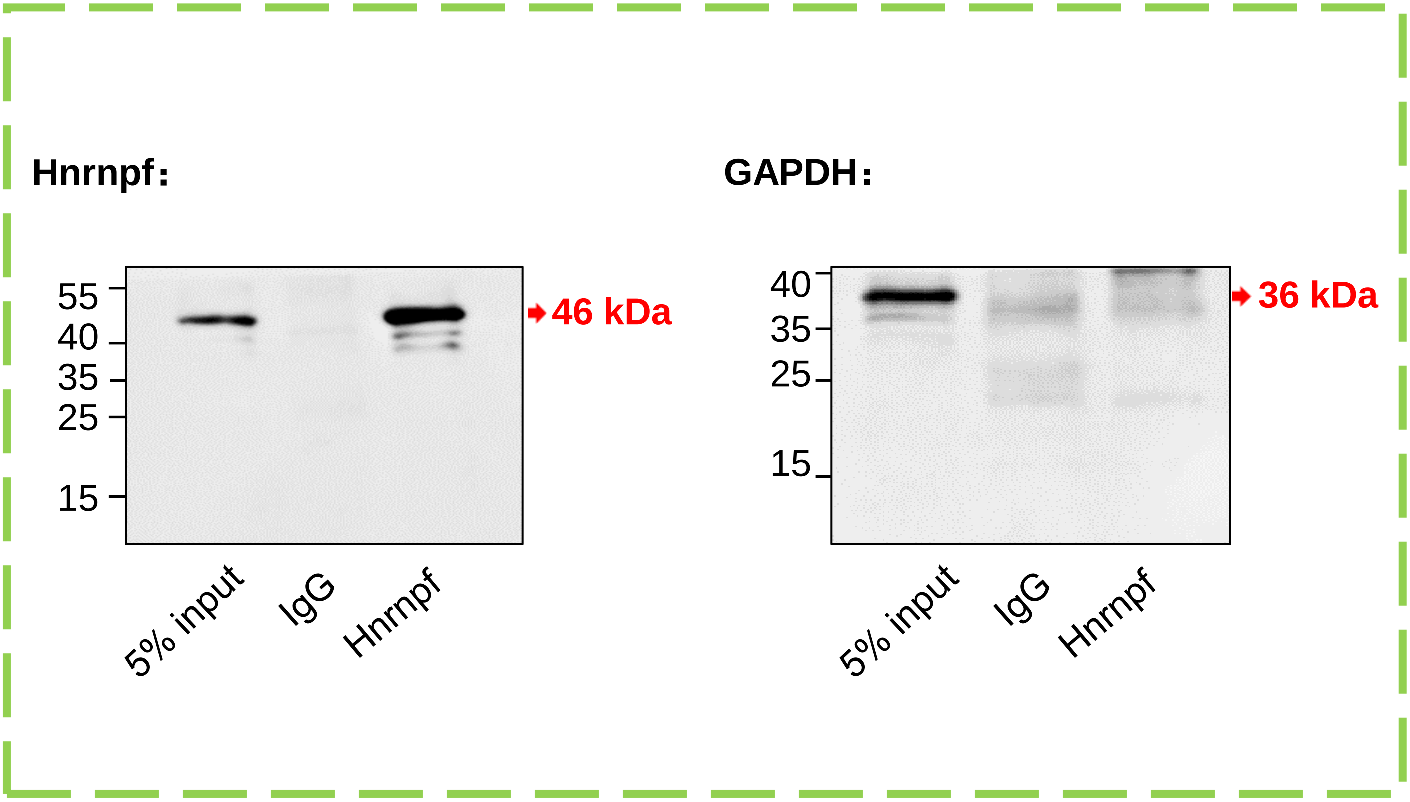
Hnrnpf：
55
40
35
25
15
46 kDa
5% input
IgG
Hnrnpf
GAPDH：
40
35
25
15
36 kDa
5% input
IgG
Hnrnpf

Figure 5D:

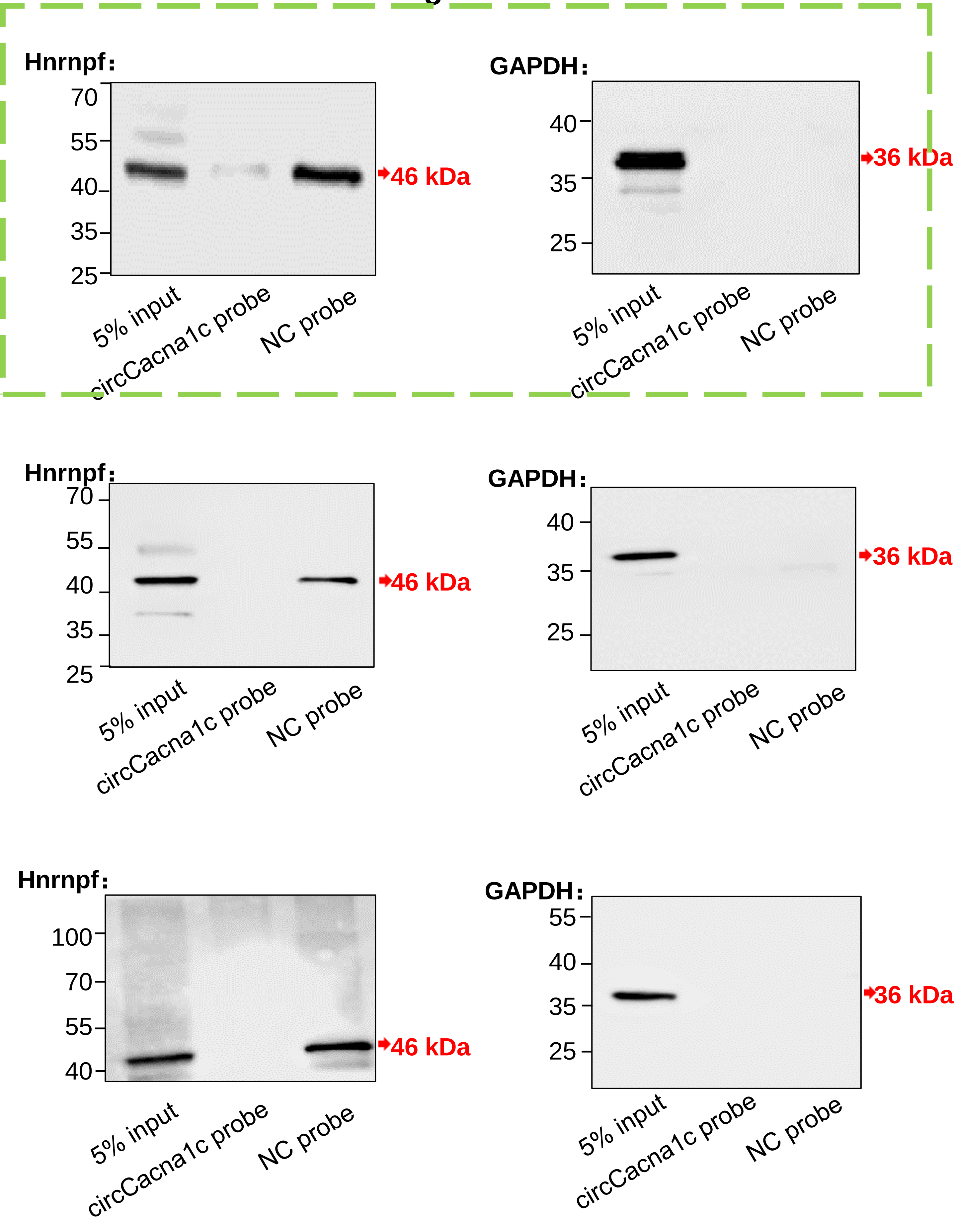
Hnrnpf:
70
55
40
35
25
46 kDa
5% input
circCacna1c probe
NC probe
GAPDH:
40
35
25
36 kDa
5% input
circCacna1c probe
NC probe
Hnrnpf:
70
55
40
35
25
46 kDa
5% input
circCacna1c probe
NC probe
GAPDH:
40
35
25
36 kDa
5% input
circCacna1c probe
NC probe
Hnrnpf:
100
70
55
40
46 kDa
5% input
circCacna1c probe
NC probe
GAPDH:
55
40
35
25
36 kDa
5% input
circCacna1c probe
NC probe

Figure 5E:

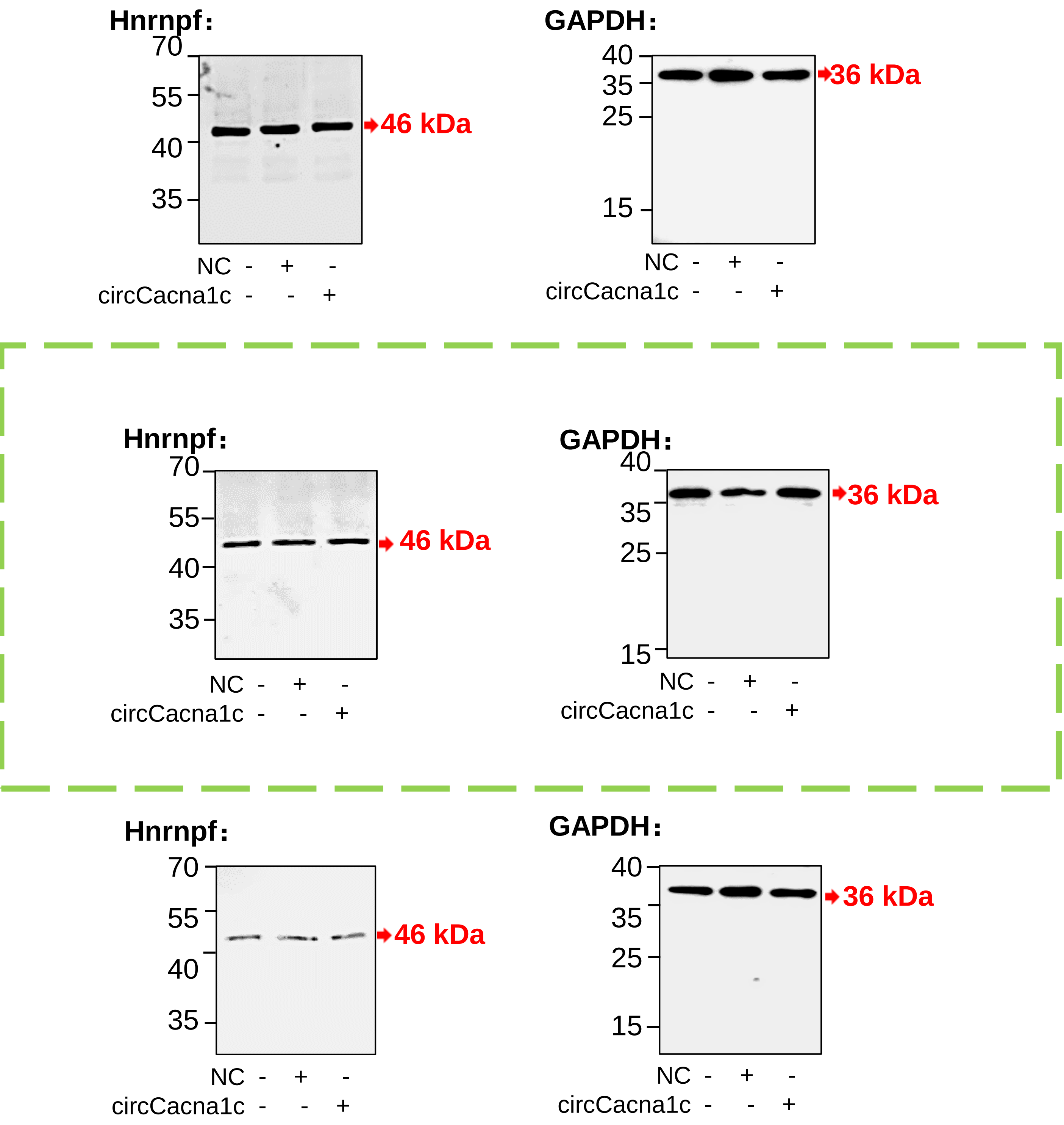
Hnrnpf:
70
55
40
35
46 kDa
NC - + -
circCacna1c - - +
GAPDH:
40
35
25
15
36 kDa
NC - + -
circCacna1c - - +
Hnrnpf:
70
55
40
35
46 kDa
NC - + -
circCacna1c - - +
GAPDH:
40
35
25
15
36 kDa
NC - + -
circCacna1c - - +
Hnrnpf:
70
55
40
35
46 kDa
NC - + -
circCacna1c - - +
GAPDH:
40
35
25
15
36 kDa
NC - + -
circCacna1c - - +

Figure 5F:

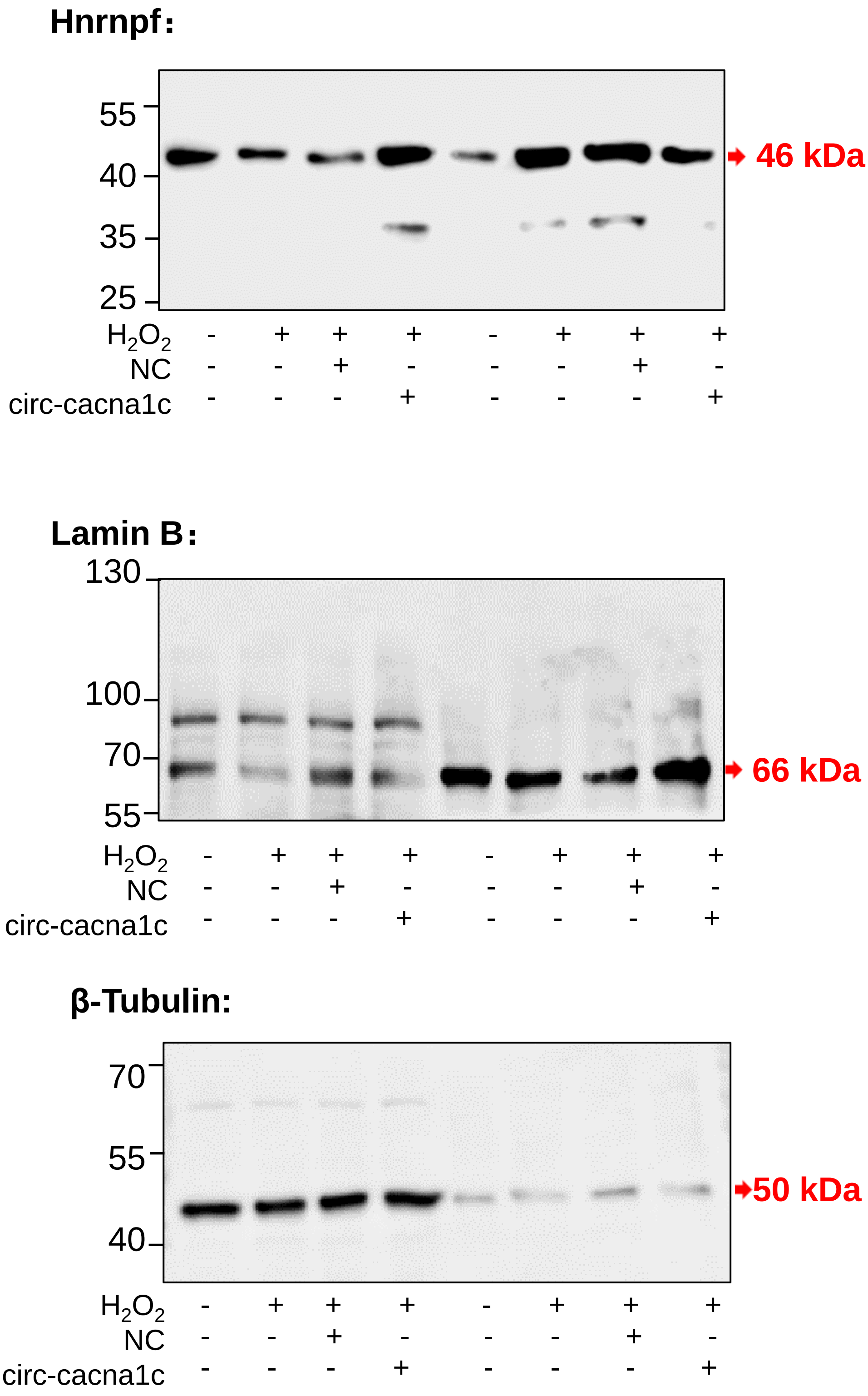

Hnrnpf:
55
40
35
25
46 kDa
H2O2 - + + + - + + +
NC - - + - - - + -
circc-acna1c - - - + - - - +
Lamin B:
130
100
70
55
66 kDa
H2O2 - + + + - + + +
NC - - + - - - + -
circ-cacna1c - - - + - - - +
β-Tubulin:
70
55
40
50 kDa
H2O2 - + + + - + + +
NC - - + - - - + -
circ-cacna1c - - - + - - - +

Figure 5F:

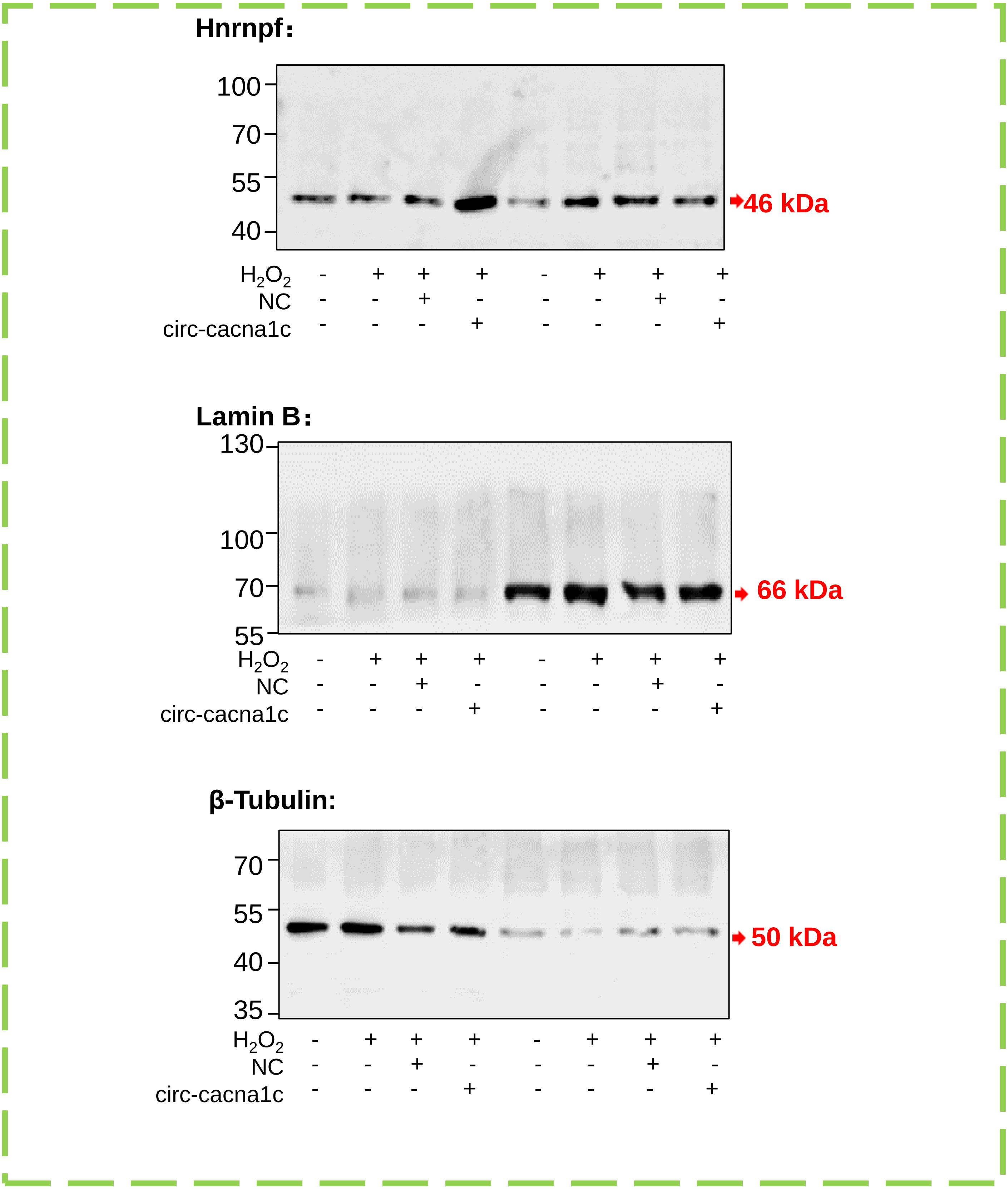
Hnrnpf:
100
70
55
40
46 kDa
H_2O_2 - + + + - + + +
NC - - + - - - + -
circ-cacna1c - - - + - - - +
Lamin B:
130
100
70
55
66 kDa
H_2O_2 - + + + - + + +
NC - - + - - - + -
circ-cacna1c - - - + - - - +
β-Tubulin:
70
55
40
35
50 kDa
H_2O_2 - + + + - + + +
NC - - + - - - + -
circ-cacna1c - - - + - - - +

Figure 5F:

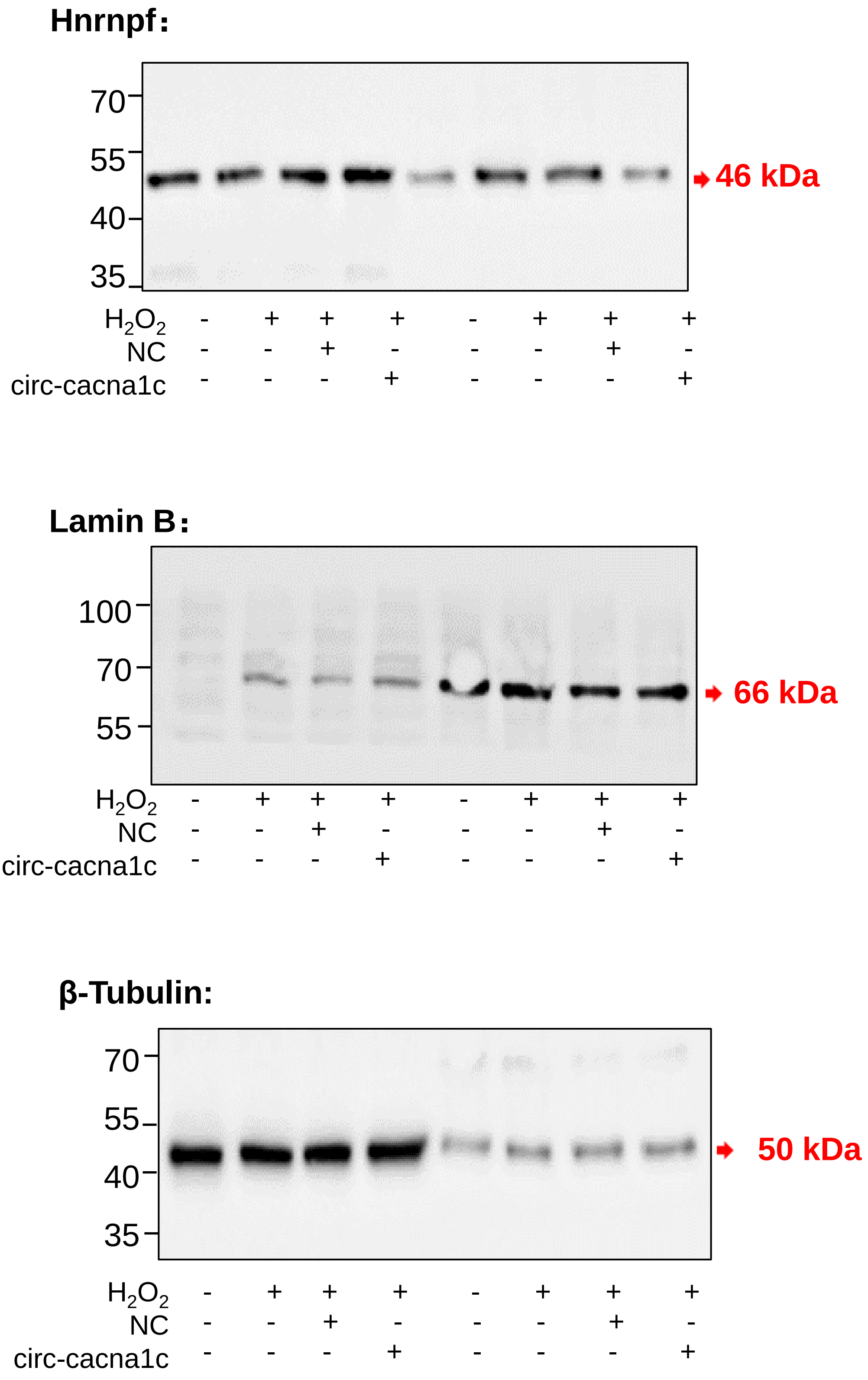
Hnrnpf:
70
55
40
35
46 kDa
H2O2 - + + + - + + +
NC - - + - - - + -
circ-cacna1c - - - + - - - +
Lamin B:
100
70
55
66 kDa
H2O2 - + + + - + + +
NC - - + - - - + -
circ-cacna1c - - - + - - - +
β-Tubulin:
70
55
40
35
50 kDa
H2O2 - + + + - + + +
NC - - + - - - + -
circ-cacna1c - - - + - - - +

Figure 5G:

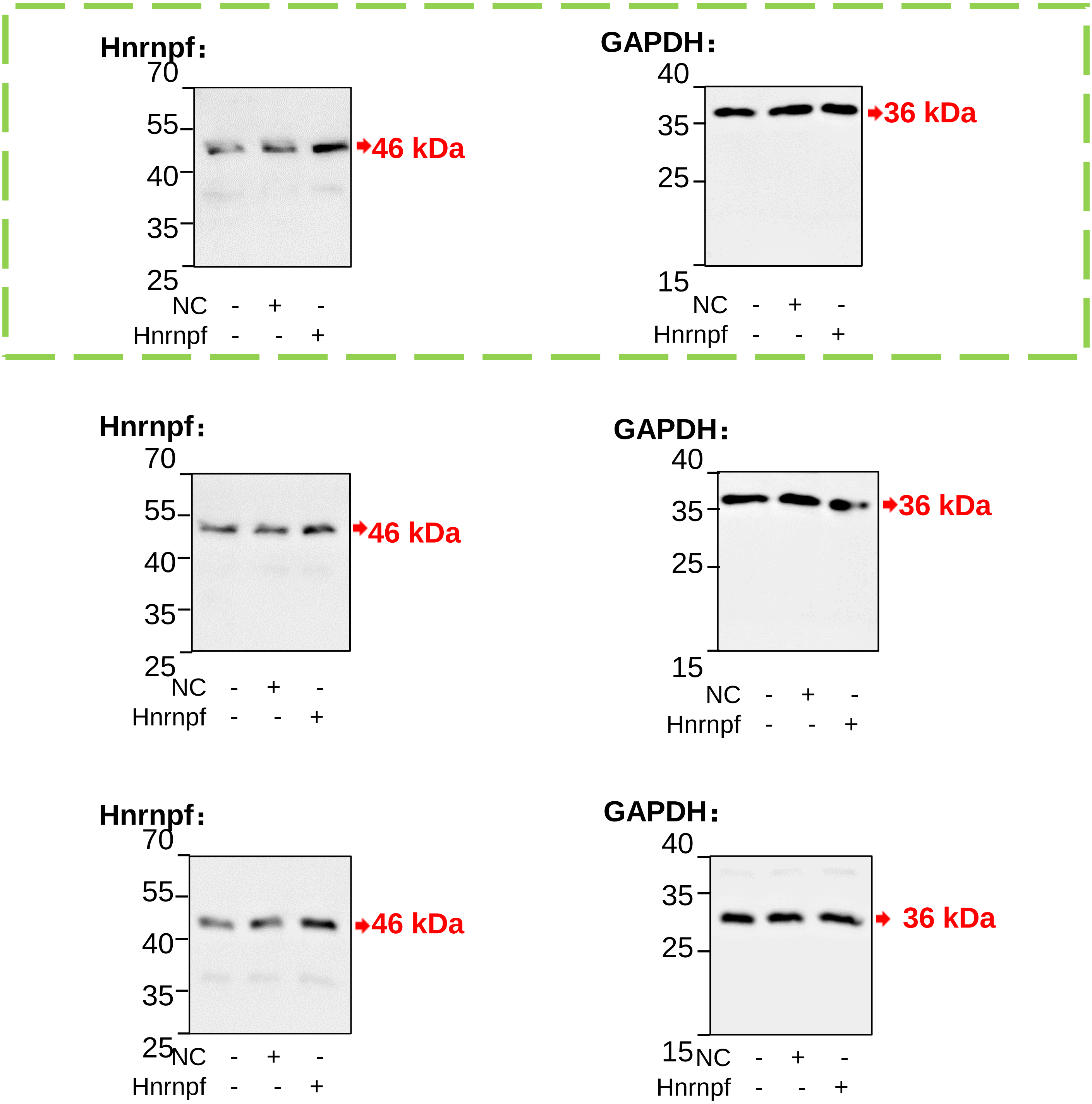

Hnrnpf:
70
55
40
35
25
46 kDa
NC - + -
Hnrnpf - - +
GAPDH:
40
35
25
15
36 kDa
NC - + -
Hnrnpf - - +
Hnrnpf:
70
55
40
35
25
46 kDa
NC - + -
Hnrnpf - - +
GAPDH:
40
35
25
15
36 kDa
NC - + -
Hnrnpf - - +
Hnrnpf:
70
55
40
35
25
46 kDa
NC - + -
Hnrnpf - - +
GAPDH:
40
35
25
15
36 kDa
NC - + -
Hnrnpf - - +

Figure 6E:

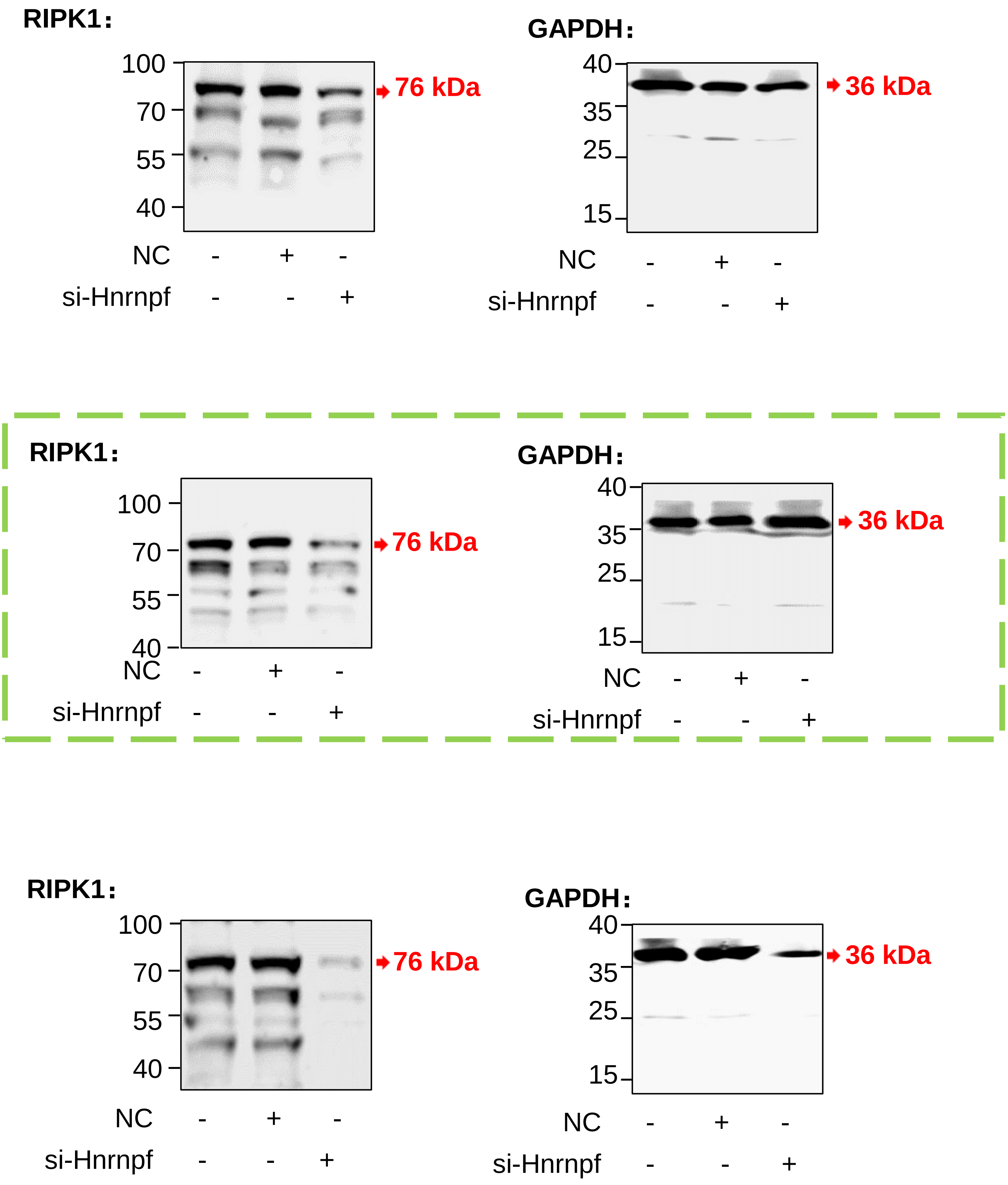
RIPK1:
100
70
55
40
76 kDa
NC - + -
si-Hnrnpf - - +
GAPDH:
40
35
25
15
36 kDa
NC - + -
si-Hnrnpf - - +
RIPK1:
100
70
55
40
76 kDa
NC - + -
si-Hnrnpf - - +
GAPDH:
40
35
25
15
36 kDa
NC - + -
si-Hnrnpf - - +
RIPK1:
100
70
55
40
76 kDa
NC - + -
si-Hnrnpf - - +
GAPDH:
40
35
25
15
36 kDa
NC - + -
si-Hnrnpf - - +

Figure 6H:

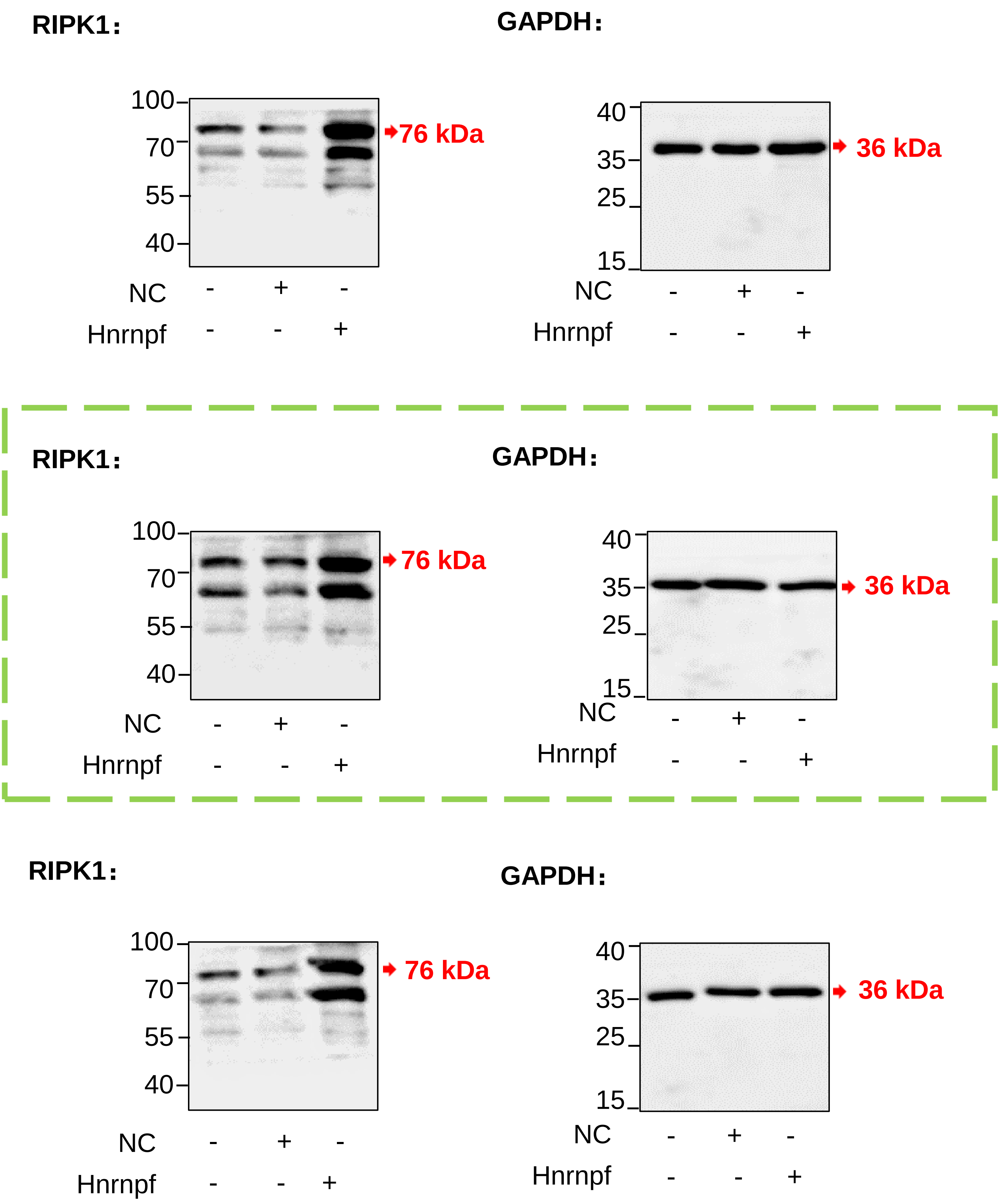
RIPK1:
GAPDH:
100
70
55
40
76 kDa
40
35
25
15
36 kDa
NC - + -
Hnrnpf - - +
RIPK1:
GAPDH:
100
70
55
40
76 kDa
40
35
25
15
36 kDa
NC - + -
Hnrnpf - - +
RIPK1:
GAPDH:
100
70
55
40
76 kDa
40
35
25
15
36 kDa
NC - + -
Hnrnpf - - +

Figure 6J:

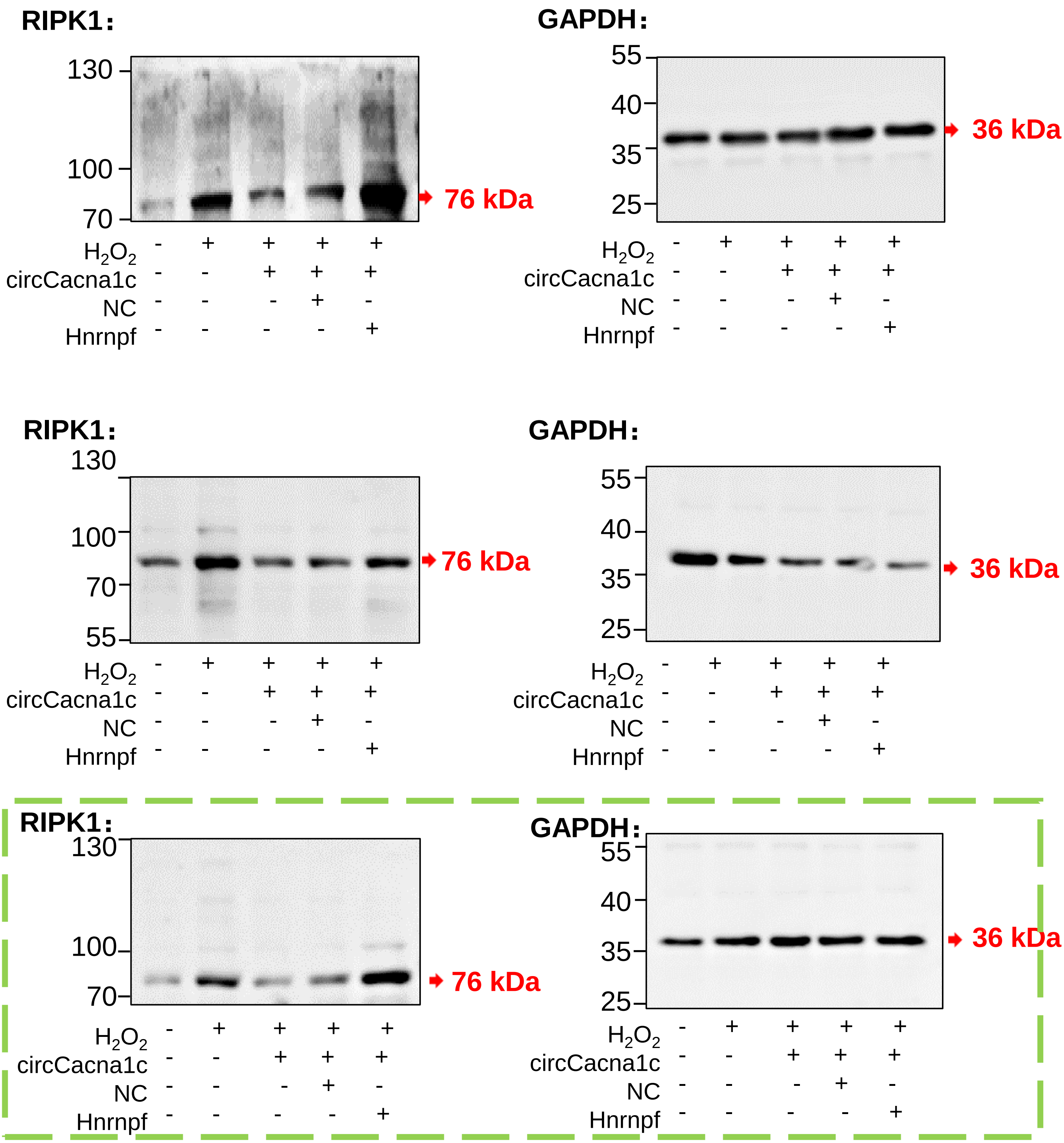
RIPK1:
130
100
70
76 kDa
H_2O_2 - + + + +
circCacna1c - - + + +
NC - - - + -
Hnrnpf - - - - +
GAPDH:
55
40
35
25
36 kDa
H_2O_2 - + + + +
circCacna1c - - + + +
NC - - - + -
Hnrnpf - - - - +
RIPK1:
130
100
70
55
76 kDa
H_2O_2 - + + + +
circCacna1c - - + + +
NC - - - + -
Hnrnpf - - - - +
GAPDH:
55
40
35
25
36 kDa
H_2O_2 - + + + +
circCacna1c - - + + +
NC - - - + -
Hnrnpf - - - - +
RIPK1:
130
100
70
76 kDa
H_2O_2 - + + + +
circCacna1c - - + + +
NC - - - + -
Hnrnpf - - - - +
GAPDH:
55
40
35
25
36 kDa
H_2O_2 - + + + +
circCacna1c - - + + +
NC - - - + -
Hnrnpf - - - - +

Figure 6L:

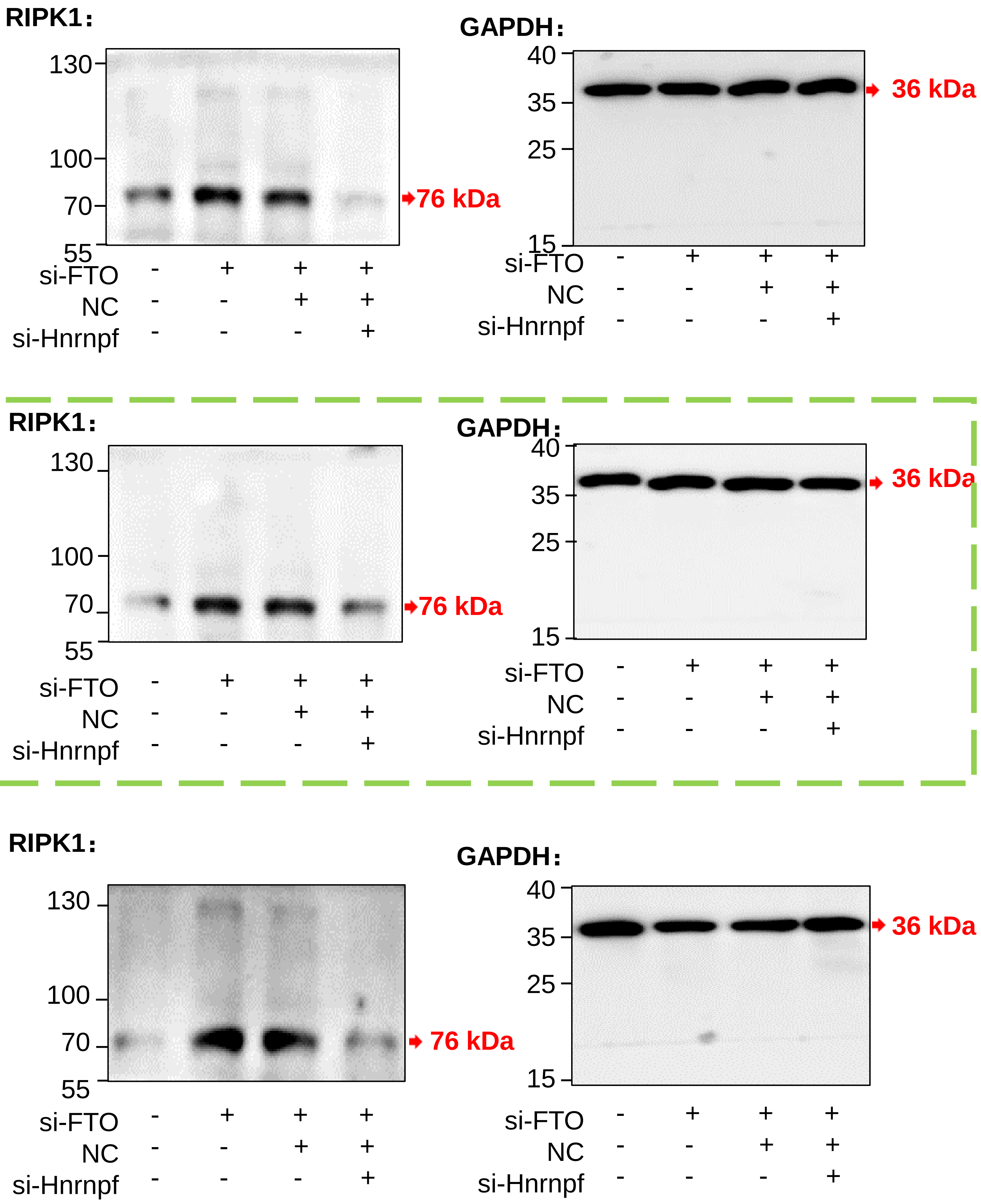
RIPK1:
130
100
70
55
76 kDa
si-FTO - + + +
NC - - + +
si-Hnrnpf - - - +
GAPDH:
40
35
25
15
36 kDa
si-FTO - + + +
NC - - + +
si-Hnrnpf - - - +
RIPK1:
130
100
70
55
76 kDa
si-FTO - + + +
NC - - + +
si-Hnrnpf - - - +
GAPDH:
40
35
25
15
36 kDa
si-FTO - + + +
NC - - + +
si-Hnrnpf - - - +
RIPK1:
130
100
70
55
76 kDa
si-FTO - + + +
NC - - + +
si-Hnrnpf - - - +
GAPDH:
40
35
25
15
36 kDa
si-FTO - + + +
NC - - + +
si-Hnrnpf - - - +

Figure 7C:

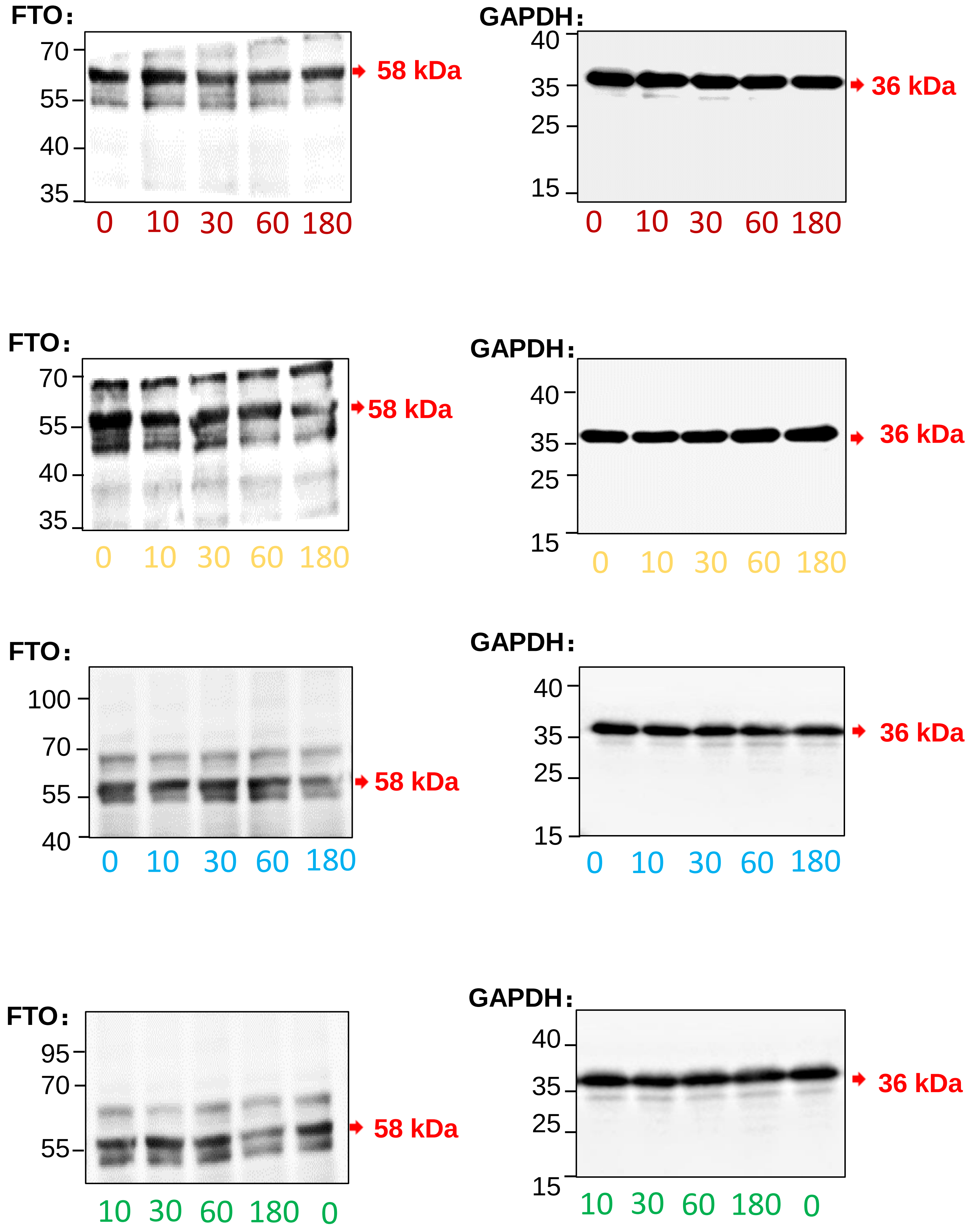
FTO:
70
55
40
35
58 kDa
0 10 30 60 180
GAPDH:
40
35
25
15
36 kDa
0 10 30 60 180
FTO:
70
55
40
35
58 kDa
0 10 30 60 180
GAPDH:
40
35
25
15
36 kDa
0 10 30 60 180
FTO:
100
70
55
40
58 kDa
0 10 30 60 180
GAPDH:
40
35
25
15
36 kDa
0 10 30 60 180
FTO:
95
70
55
58 kDa
10 30 60 180 0
GAPDH:
40
35
25
15
36 kDa
10 30 60 180 0

Figure 7C:

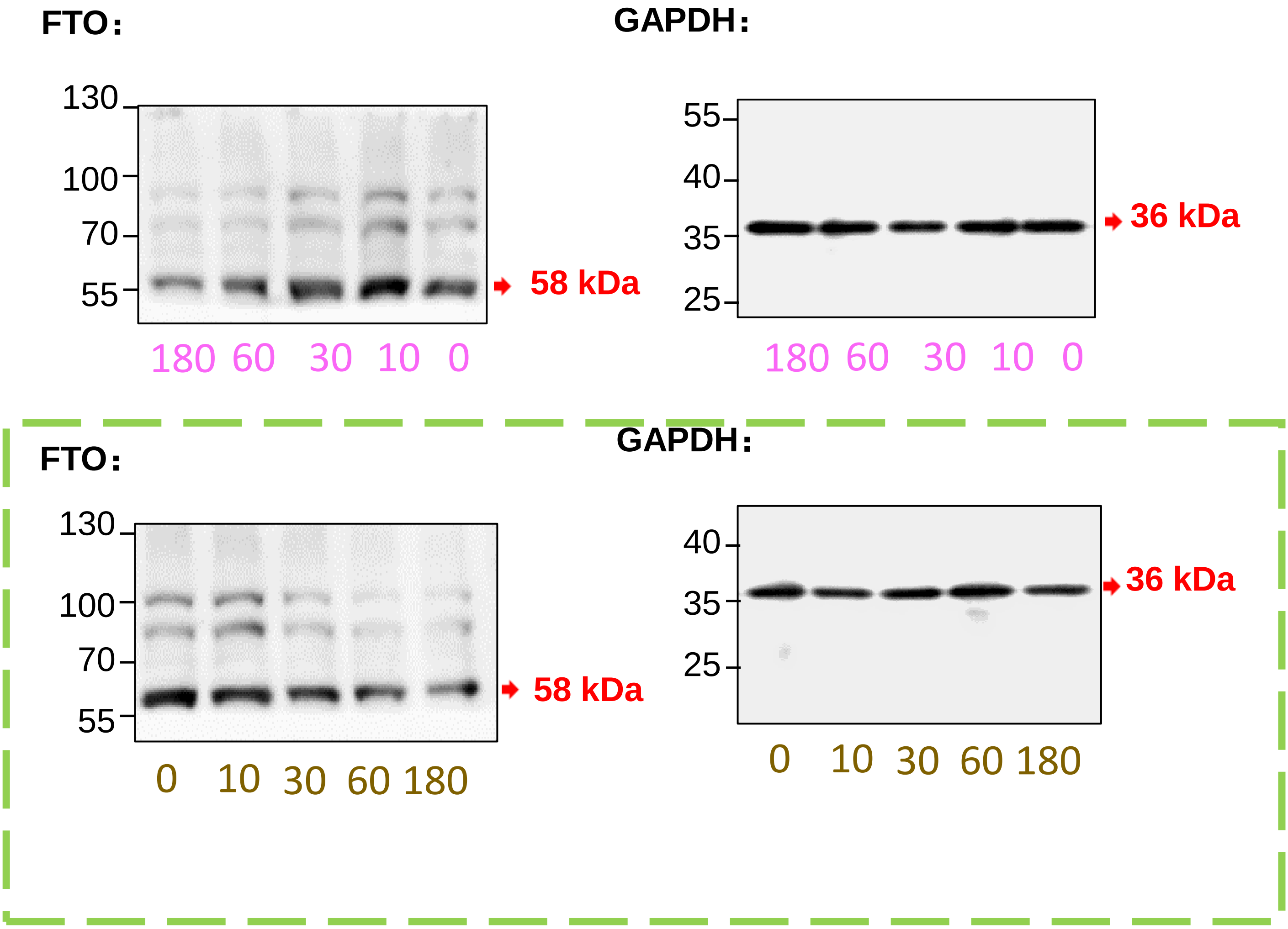
FTO:
GAPDH:
130
100
70
55
58 kDa
55
40
35
25
36 kDa
180 60 30 10 0
180 60 30 10 0
FTO:
GAPDH:
130
100
70
55
58 kDa
40
35
25
36 kDa
0 10 30 60 180
0 10 30 60 180

Figure 7H:

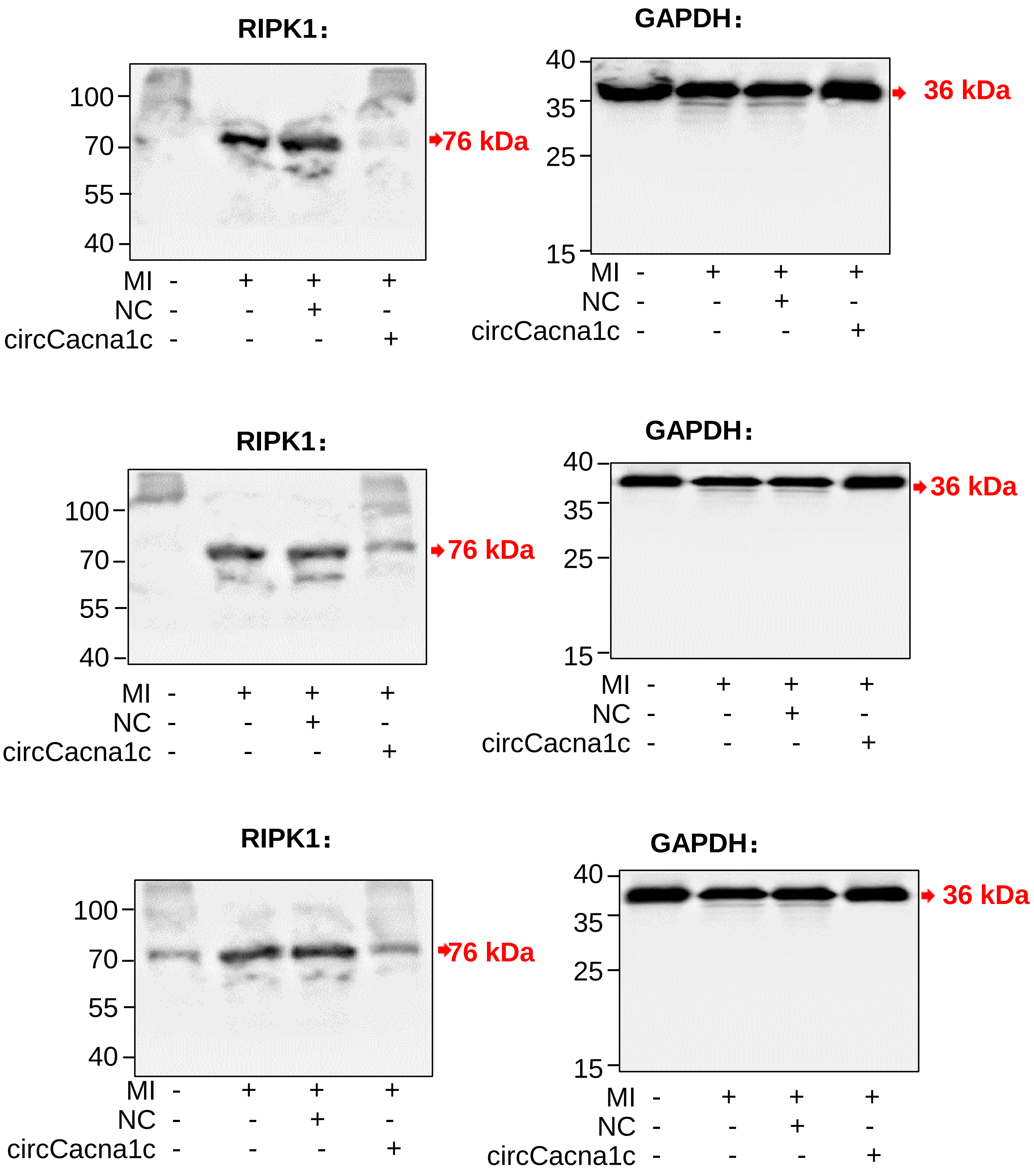
RIPK1:
100
70
55
40
76 kDa
MI - + + +
NC - - + -
circCacna1c - - - +
GAPDH:
40
35
25
15
36 kDa
MI - + + +
NC - - + -
circCacna1c - - - +
RIPK1:
100
70
55
40
76 kDa
MI - + + +
NC - - + -
circCacna1c - - - +
GAPDH:
40
35
25
15
36 kDa
MI - + + +
NC - - + -
circCacna1c - - - +
RIPK1:
100
70
55
40
76 kDa
MI - + + +
NC - - + -
circCacna1c - - - +
GAPDH:
40
35
25
15
36 kDa
MI - + + +
NC - - + -
circCacna1c - - - +

Figure 7H:

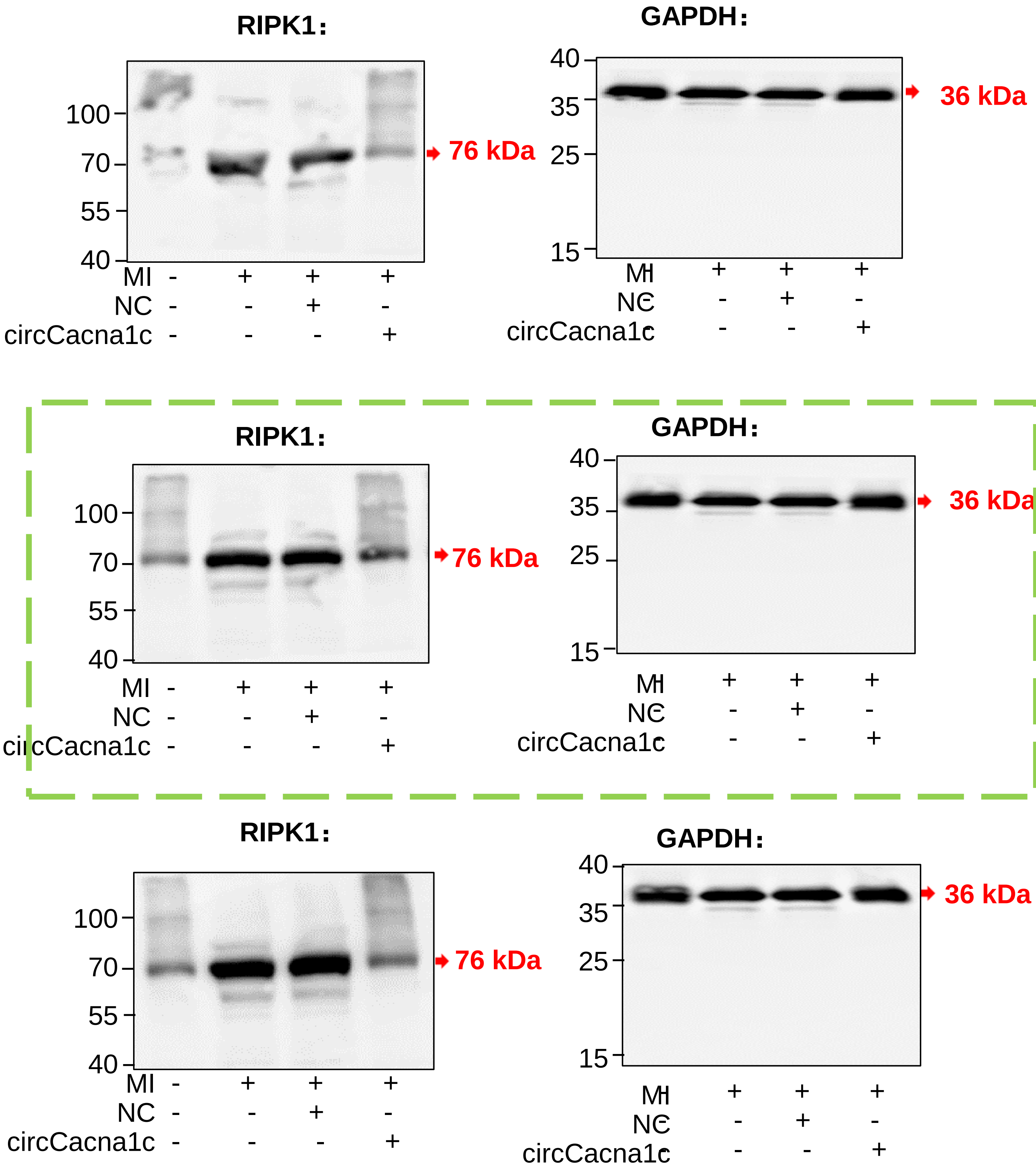
RIPK1:
100
70
55
40
76 kDa
MI - + + +
NC - - + -
circCacna1c - - - +
GAPDH:
40
35
25
15
36 kDa
MI + + +
NC - + -
circCacna1c - - +
RIPK1:
100
70
55
40
76 kDa
MI - + + +
NC - - + -
circCacna1c - - - +
GAPDH:
40
35
25
15
36 kDa
MI + + +
NC - + -
circCacna1c - - +
RIPK1:
100
70
55
40
76 kDa
MI - + + +
NC - - + -
circCacna1c - - - +
GAPDH:
40
35
25
15
36 kDa
MI + + +
NC - + -
circCacna1c - - +

figure S5B：

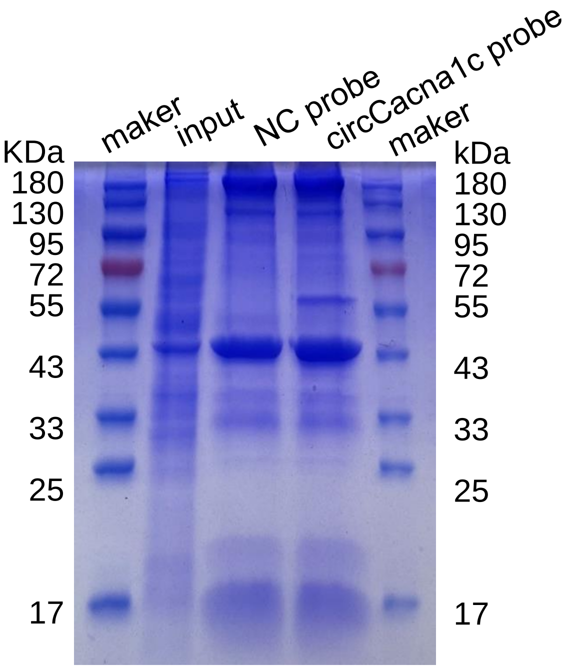

KDa
maker
input
NC probe
circCacna1c probe
maker
kDa
180
130
95
72
55
43
33
25
17

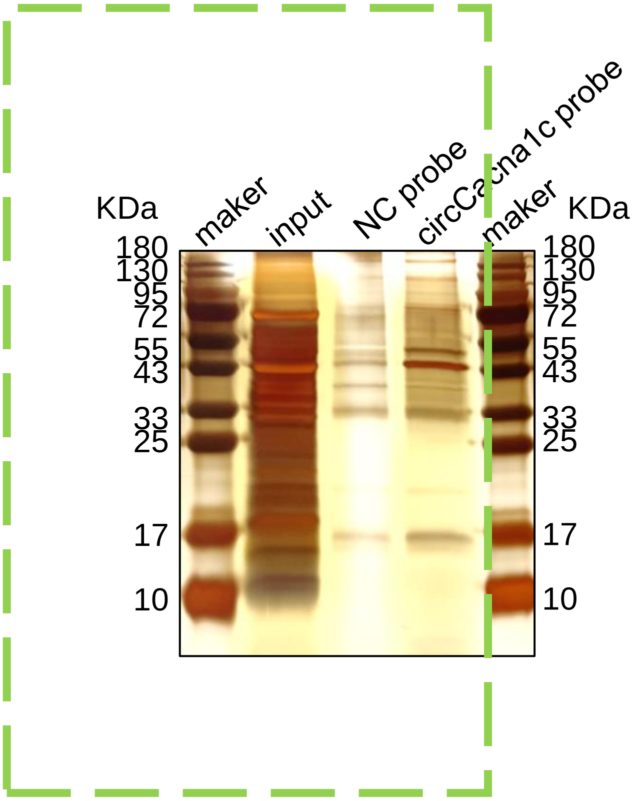

KDa
maker
input
NC probe
circCacna1c probe
maker
KDa
180
130
95
72
55
43
33
25
17
10

figure S5E :

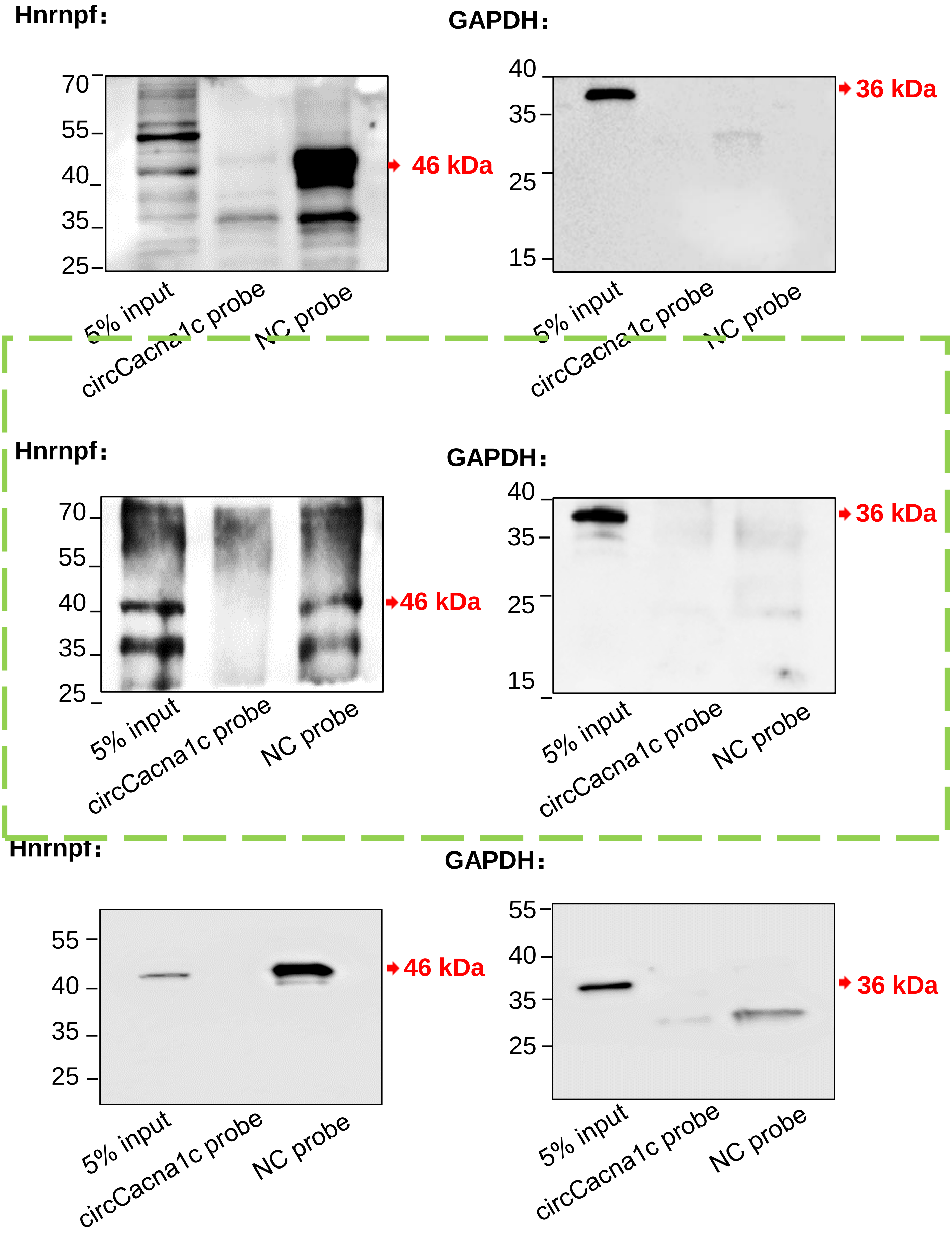

Hnrnpf:
70
55
40
35
25
46 kDa
5% input
circCacna1c probe
NC probe
GAPDH:
40
35
25
15
36 kDa
5% input
circCacna1c probe
NC probe
Hnrnpf:
70
55
40
35
25
46 kDa
5% input
circCacna1c probe
NC probe
GAPDH:
40
35
25
15
36 kDa
5% input
circCacna1c probe
NC probe
Hnrnpf:
55
40
35
25
46 kDa
5% input
circCacna1c probe
NC probe
GAPDH:
55
40
35
25
36 kDa
5% input
circCacna1c probe
NC probe

figure S5G:

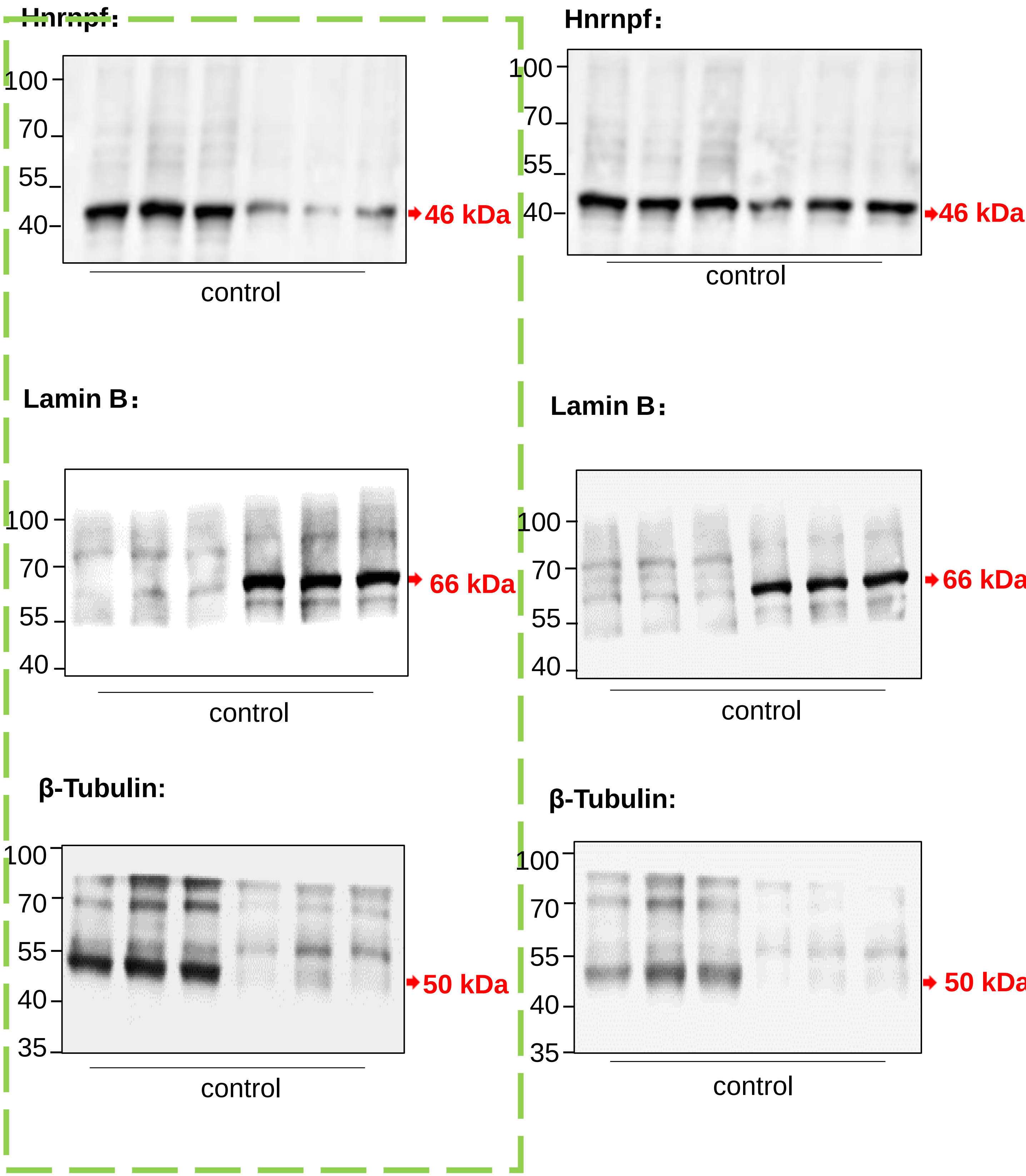

Hnrnpf:
100
70
55
40
46 kDa
control
Hnrnpf:
100
70
55
40
46 kDa
control
Lamin B:
100
70
55
40
66 kDa
control
Lamin B:
100
70
55
40
66 kDa
control
β-Tubulin:
100
70
55
40
35
50 kDa
control
β-Tubulin:
100
70
55
40
35
50 kDa
control

figure S5H:

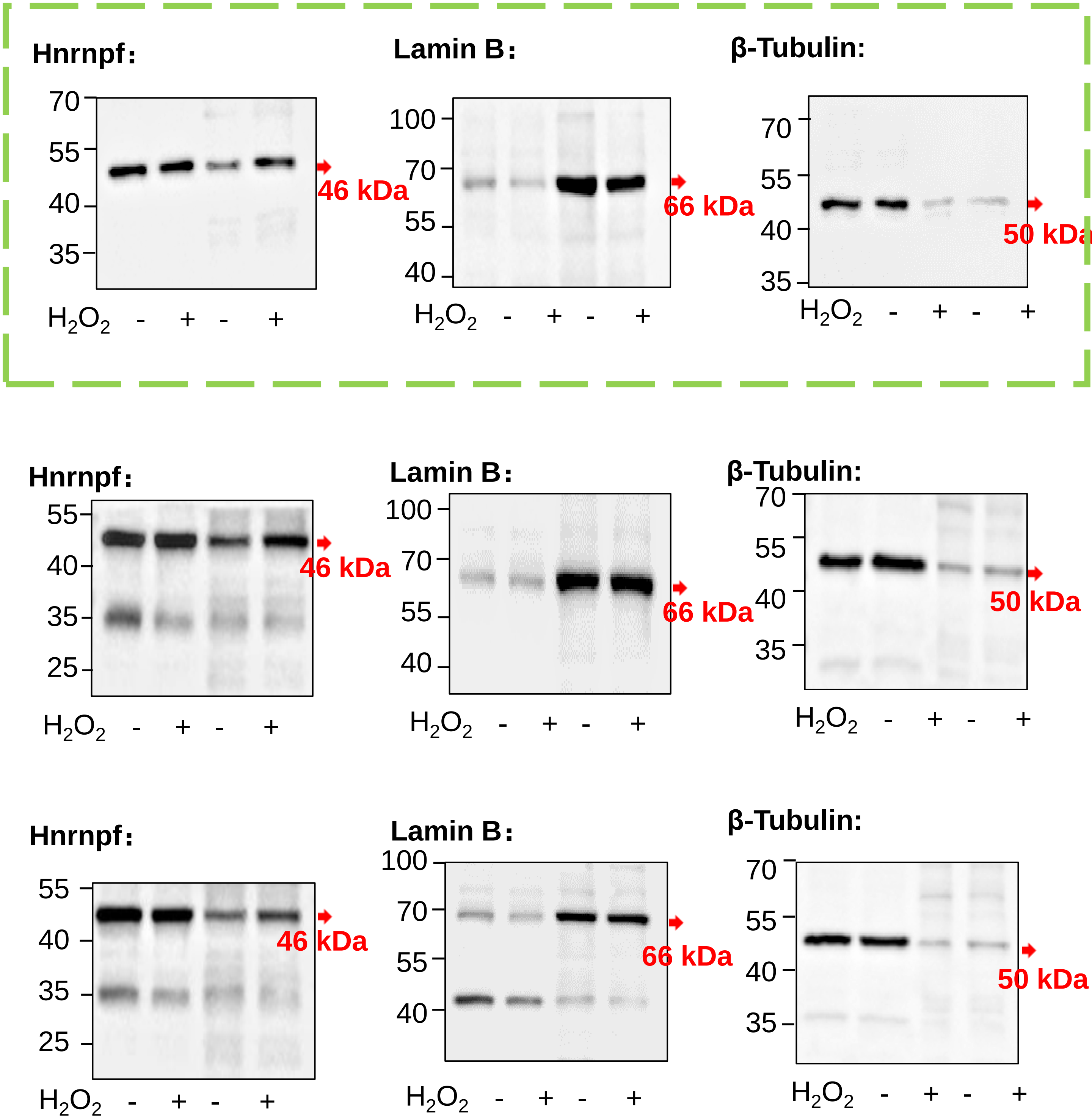
Hnrnpf:
Lamin B:
β-Tubulin:
70
55
40
35
25
100
46 kDa
66 kDa
50 kDa
H_2O_2 - + - +

figure S5I：

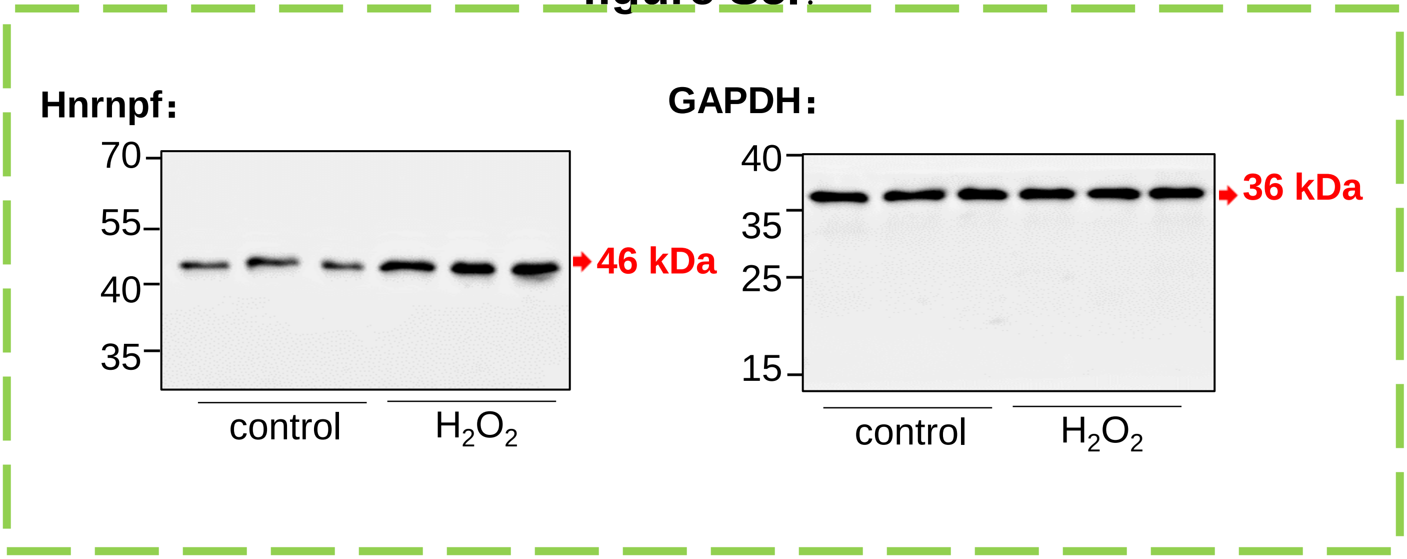
Hnrnpf：
70
55
40
35
46 kDa
control
H2O2
GAPDH：
40
35
25
15
36 kDa
control
H2O2

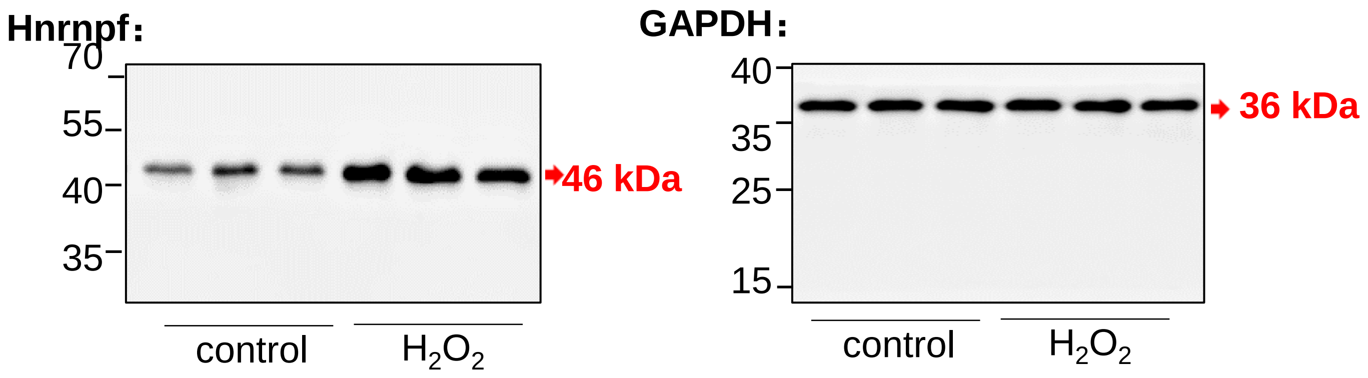
Hnrnpf：
70
55
40
35
46 kDa
control
H2O2
GAPDH：
40
35
25
15
36 kDa
control
H2O2

figure S5J:

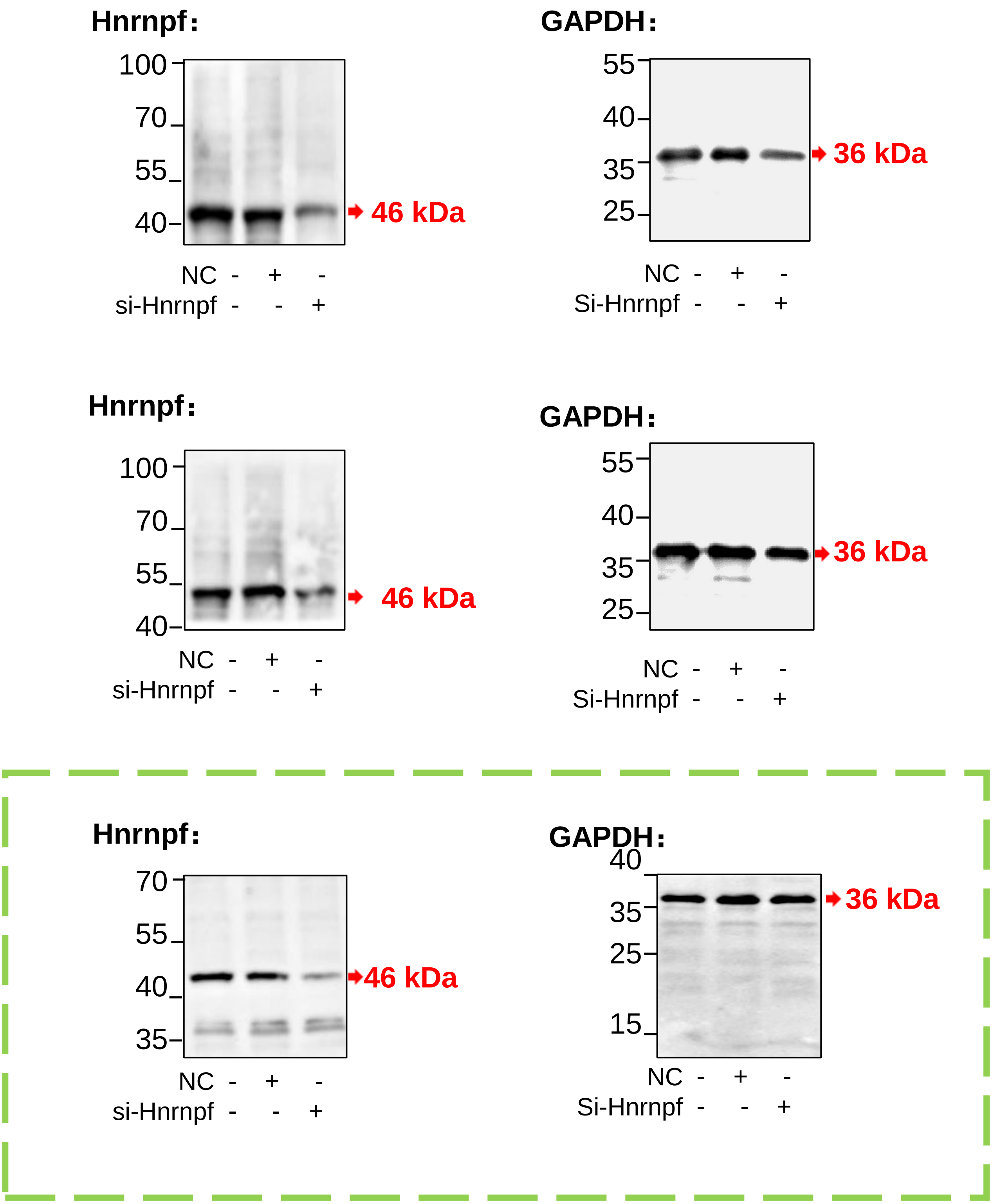
Hnrnpf:
100
70
55
40
46 kDa
NC - + -
si-Hnrnpf - - +
GAPDH:
55
40
35
25
36 kDa
NC - + -
Si-Hnrnpf - - +
Hnrnpf:
100
70
55
40
46 kDa
NC - + -
si-Hnrnpf - - +
GAPDH:
55
40
35
25
36 kDa
NC - + -
Si-Hnrnpf - - +
Hnrnpf:
70
55
40
35
46 kDa
NC - + -
si-Hnrnpf - - +
GAPDH:
40
35
25
15
36 kDa
NC - + -
Si-Hnrnpf - - +
